# Redox chemistry and H-atom abstraction reactivity of a terminal zirconium(iv) oxo compound mediated by an appended cobalt(i) center†

**DOI:** 10.1039/d0sc04229c

**Published:** 2020-09-04

**Authors:** Hongtu Zhang, Gillian P. Hatzis, Diane A. Dickie, Curtis E. Moore, Christine M. Thomas

**Affiliations:** Department of Chemistry and Biochemistry, The Ohio State University 100 W. 18th Ave Columbus OH 43210 USA thomasc@chemistry.ohio-state.edu; Department of Chemistry, University of Virginia 409 McCormick Road, PO Box 400319 Charlottesville VA 22904 USA

## Abstract

The reactivity of the terminal zirconium(iv) oxo complex, O≡Zr(MesNP^i^Pr_2_)_3_CoCN^*t*^Bu (**2**), is explored, revealing unique redox activity imparted by the pendent redox active cobalt(i) center. Oxo complex **2** can be chemically reduced using Na/Hg or Ph_3_C^•^ to afford the Zr^IV^/Co^0^ complexes [(μ-Na)OZr(MesNP^i^Pr_2_)_3_CoCN^*t*^Bu]_2_ (**3**) and Ph_3_COZr(MesNP^i^Pr_2_)_3_CoCN^*t*^Bu (**4**), respectively. Based on the cyclic voltammogram of **2**, Ph_3_˙ should not be sufficiently reducing to achieve the chemical reduction of **2**, but sufficient driving force for the reaction is provided by the nucleophilicity of the terminal oxo fragment and its affinity to bind Ph_3_C^+^. Accordingly, **2** reacts readily with [Ph_3_C][BPh_4_] and Ph_3_CCl to afford [Ph_3_COZr(MesNP^i^Pr_2_)_3_CoCN^*t*^Bu][BPh_4_] (**[5][BPh4]**) and Ph_3_COZr(MesNP^i^Pr_2_)_3_CoCl (**6**), respectively. The chemical oxidation of **2** is also investigated, revealing that oxidation of **2** is accompanied by immediate hydrogen atom abstraction to afford the hydroxide complex [HOZr(MesNP^i^Pr_2_)_3_CoCN^*t*^Bu]^+^ (**[9]+**). Thus it is posited that the transient [OZr(MesNP^i^Pr_2_)_3_CoCN^*t*^Bu]^+^ [**2**]^+^ cation generated upon oxidation combines the basicity of a nucleophilic early metal oxo fragment with the oxidizing power of the appended cobalt center to facilitate H-atom abstraction.

## Abstract 2

Bimetallic cooperativity is demonstrated with a Co/Zr complex featuring both nucleophilic Zr(iv) oxo and redox active Co sites.

## Graphical abstract

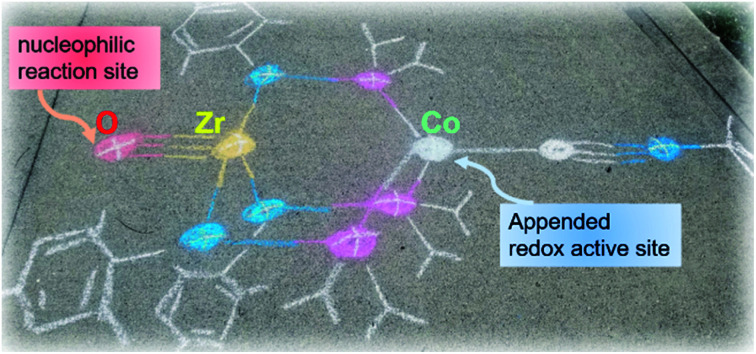

## Introduction

Metal oxo compounds are proposed to play key roles in many essential chemical transformations such as oxidation reactions,^1–8^ oxygen transfer reactions,^9,10^ C–H activations,^11^ and oxygen evolution reactions (OER).^12^ In order to understand these important oxygenation/oxidation reactions, great efforts have been made to isolate and study the electronic structure and reactivity of metal oxo compounds.

Despite the high oxophilicity^13^ and low d electron counts that should favor metal-oxo multiple bond formation, examples of terminal oxo complexes of group 4 metals (Ti, Zr, Hf) are relatively scarce, especially for Zr and Hf. Instead, group 4 metals have a strong tendency to dimerize and form oxo-bridged clusters with bridging oxides.^14–16^ The “hard” Lewis acid nature of group 4 metals enhances the basicity of their oxo ligands, which therefore exhibit a strong preference to bridge two or more metal centers.^17^ As a strategy to synthesize terminal oxo compounds with group 4 metals, sterically bulky ligands have been shown to effectively inhibit dimerization through the oxo ligand. In the limited examples of Ti oxo compounds, macrocyclic tetraaza ligand frameworks^18–20^ and coordinatively saturated octahedral geometries^21,22^ have been adopted to stabilize reactive Ti oxo moieties. Ti or Zr oxo species in (pseudo)tetrahedral geometries are often isolated in anionic form and stabilized by alkali cations,^23–25^ a strategy that also works for macrocyclic terminal oxo species.^26^ A notable example of a neutral titanocene terminal oxo complex, which was quite unstable and readily decomposed to bridging oxo species, was reported by Andersen and coworkers.^27^

**Fig. 1:**
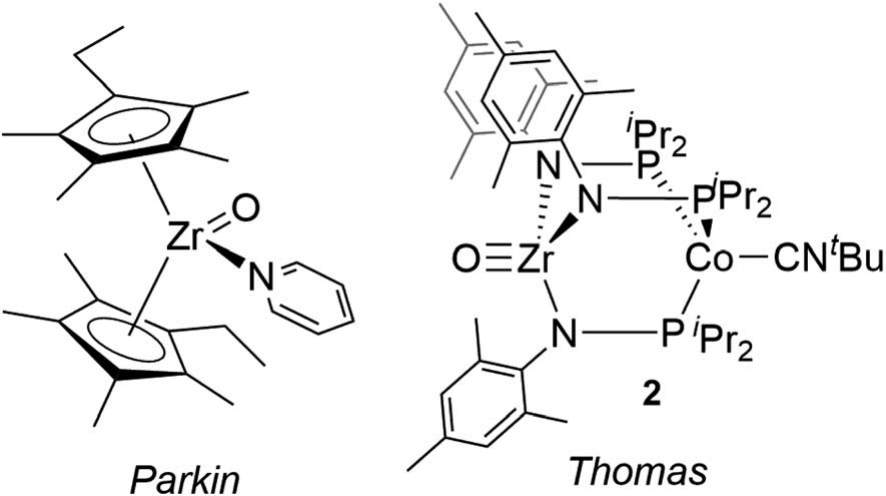
Terminal Zr oxo complexes reported by Parkin (left)^29^ and Thomas (right).^28^

Compared with Ti terminal oxo compounds, there are even fewer Zr terminal oxo species reported. Bergman and coworkers conducted detailed mechanistic and reactivity studies of a transient zirconocene terminal oxo species, generated *in situ* through heating the Zr hydroxide precursor Cp*_2_ZrPh(OH) (Cp* = *η*^5^-C_5_Me_5_).^30,31^ Subsequently, Parkin isolated the first neutral Zr terminal oxo compound, (*η*^5^-C_5_Me_4_Et)_2_Zr(O)(NC_5_H_5_) (Fig. 1), generated by 2e^−^ oxidation of Zr^II^ precursor Cp*′_2_Zr(CO)_2_ with N_2_O (Cp*′ = *η*^5^-C_5_Me_4_Et).^29,32^ This zirconocene oxo compound showed rich nucleophilic reactivity across a wide range of substrates such as water, iodomethane, silanes, and ketones. To our knowledge, Parkin's zirconocene oxo compound was the only example of a structurally characterized neutral Zr terminal oxo compound prior to our recent report of a Zr/Co heterobimetallic oxo compound (Fig. 1, *vide infra*).^28^

The synthesis of a bimetallic terminal Zr oxo compound adds to the growing body of literature on bimetallic complexes, which have received interest for applications in small molecule activation and catalysis.^33–36^ Recently, our group reported the early/late heterobimetallic compound (THF)Zr(MesNP^i^Pr_2_)_3_CoCN^*t*^Bu (**1**),^37^ derived from (THF)Zr(MesNP^i^Pr_2_)_3_CoN_2_ ^38^*via* replacement of N_2_ with CN^*t*^Bu. The tightly bound CN^*t*^Bu apical ligand sterically protects the Co site and considerably alters the reaction profile of the bimetallic compound. In studies of the reactivity of (THF)Zr(MesNP^i^Pr_2_)_3_CoN_2_, many bond activation processes involve simultaneous substrate binding to both metal centers facilitated by dissociation of the labile N_2_ ligand;^39–44^ however, the isoelectronic Zr^IV^/Co^−I^ complex **1** lacks an accessible coordination site on Co, requiring Zr to mediate substrate activation while the Co center acts as a redox active metalloligand providing electrons for substrate reduction. This redox-active metalloligand approach was first showcased in oxidation reactions with organic azides to afford terminal Zr imido species.^37^ More recently, complex **1** was shown to activate O_2_ to afford the two-electron oxidized peroxo species (O_2_)Zr(MesNP^i^Pr_2_)_3_CoCN^*t*^Bu and to react readily with O-atom transfer reagents to afford the terminal oxo compound O≡Zr(MesNP^i^Pr_2_)_3_CoCN^*t*^Bu (**2**), shown in Fig. 1.^28^ With the appended Co center, complex **2** has the potential for further redox chemistry in addition to nucleophilic reactivity, a marked difference compared to the reaction space available to the monometallic zirconocene terminal oxo compound. Herein, we describe the reactivity and redox chemistry of complex **2.**

## Results and discussion

### Cyclic voltammetry of O≡Zr(MesNP^i^Pr_2_)_3_CoCN^*t*^Bu (**2**)

**Fig. 2:**
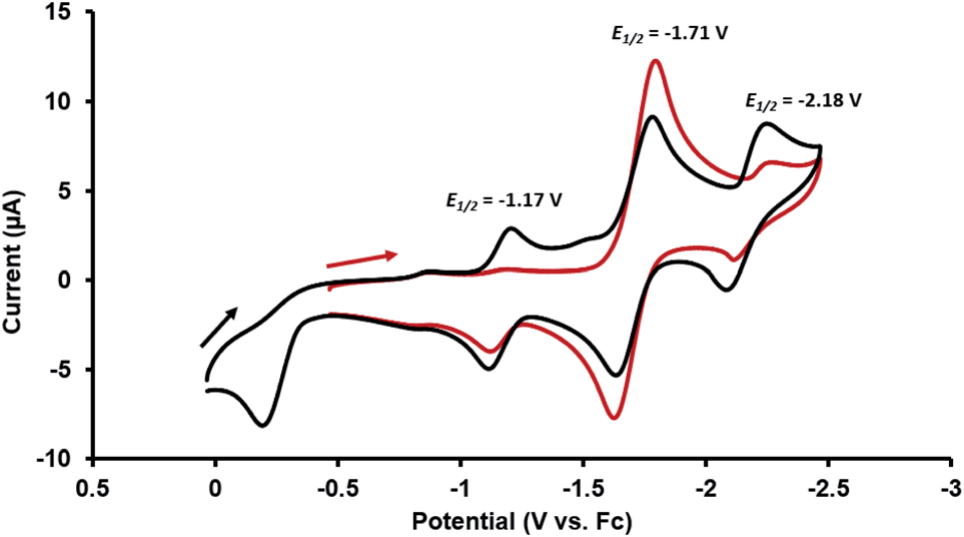
Cyclic voltammograms of **2** (in 0.3 M [^*n*^Bu_4_N][PF_6_] in THF; scan rate: 100 mV s^−1^) scanning cathodically starting from the open circuit potential (red) and starting from 0 V (black) *vs.* Fc/Fc^+^.

Given the presence of a redox active Co^I^ center in the heterobimetallic Zr^IV^/Co^I^ complex **2**, the redox behavior of this compound was examined using cyclic voltammetry. The cyclic voltammogram (CV) of **2** showed both oxidative and reductive features. When scanned cathodically starting from the open circuit potential (Fig. 2, red), the CV showed a reversible reduction at −1.71 V (*vs.* the ferrocene/ferrocenium redox couple, Fc/Fc^+^) assigned as the Co^I/0^ redox couple. If the cathodic scan was, instead, started at a more positive potential (0 V *vs.* Fc/Fc^+^, Fig. 2, black), an irreversible oxidation process was observed at −0.21 V. After scanning through potentials sufficiently oxidizing to carry out this oxidation process, two additional reversible waves appeared at −1.17 V and −2.18 V, revealing that upon oxidation of **2**, a subsequent chemical step occurred to generate a new stable Zr/Co complex that can readily access two reversible redox processes. The rich redox behavior of oxo compound **2** enabled by the ancillary Co center was further explored through chemical oxidation and reduction reactions and characterization of the resulting products.

### Reduction of O≡Zr(MesNP^i^Pr_2_)_3_CoCN^*t*^Bu (**2**)

**Scheme 1:**
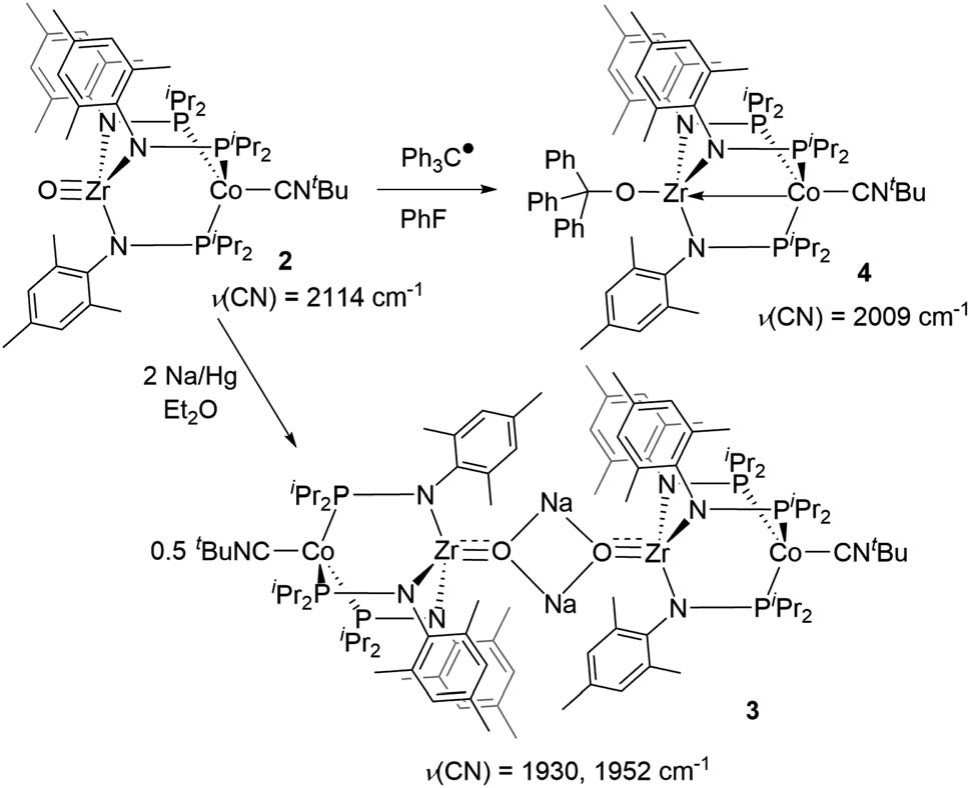
Chemical reduction of **2** using Na/Hg and Ph_3_C˙.

First, the chemical reduction of oxo complex **2** was investigated through treatment of **2** with Na/Hg amalgam. Treatment of **2** with excess Na/Hg in diethyl ether solution resulted in the formation of [(μ-Na)OZr(MesNP^i^Pr_2_)_3_CoCN^*t*^Bu]_2_ (**3**, Scheme 1). During the course of the reduction, the reaction mixture converted from a bright green suspension to a homogeneous orange solution, from which the light-yellow crystalline product **3** was isolated as the major product. The ^1^H NMR spectrum of **3** showed 6 paramagnetically shifted resonances in a pattern indicative of *C*_3_ symmetry and similar to that of previously reported *S* = 1/2 tris(phosphinoamide) Zr/Co species.^25,39,45^ The solid-state IR spectrum of **3** has two C≡N stretches at 1930 cm^−1^ and 1952 cm^−1^. The C≡N stretches are lower in frequency compared to the neutral oxo precursor **2** (*ν*(CN) = 2114 cm^−1^)^28^ as a result of stronger backbonding from the reduced Co center to the CN^*t*^Bu ligand.

**Fig. 3:**
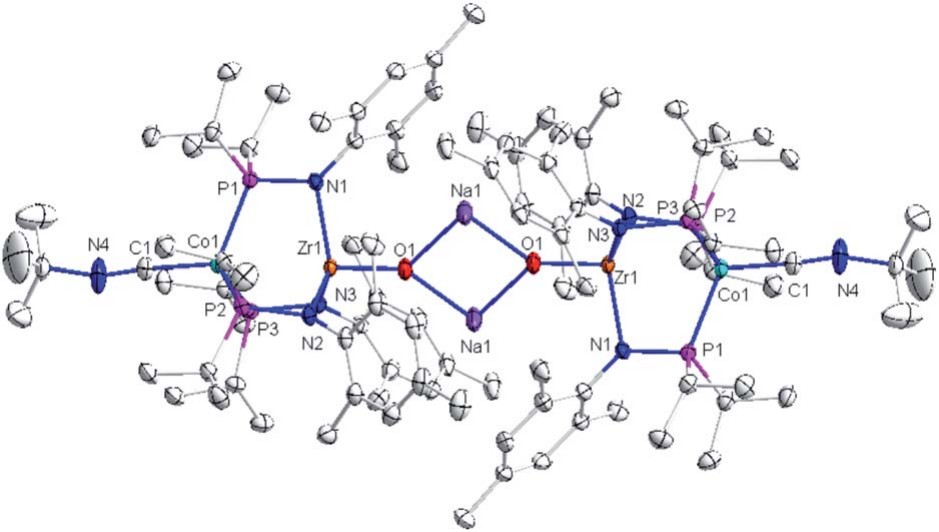
Displacement ellipsoid (50%) representation of anionic oxo compounds **3.** Hydrogen atoms and solvate molecules were omitted for clarity. Selected interatomic distances (Å): Zr1–Co1, 2.8174(3); Zr1–O1, 1.8589(15).

The solid-state structure of compound **3** revealed a tetrametallic dimeric structure in which the Zr-bound anionic oxo ligands were bridged by two sodium cations to connect the two Zr/Co fragments (Fig. 3). Compound **3** bears structural and spectroscopic similarities to the previously reported carbonyl derivatives of the general form [OZr(MesNP^i^Pr_2_)_3_CoCO]^−^.^25^ The Zr–Co distance in **3** is 2.8174(3) Å, approximately 0.2 Å shorter than that of the oxo compound **2** (3.0157(2) Å)^28^ owing to both the more reduced Co center and the weakened Zr–O bonding due to the competing interaction between the oxo ligand and the alkali cation. The Zr–O bond distance in compound **3** is, indeed, elongated compared to the oxo compound (1.8589(15) Å in **3***vs.* 1.7956(9) Å in **2**), indicative of disruption of the Zr–O multiple bonding. The Zr–O distance in **3** is comparable to that of [(12-crown-4)Li][OZr(MesNP^i^Pr_2_)_3_CoCO] (1.8328(11) Å), which was assigned a Zr≡O triple bond.^25^ Although one σ and two π bonds remain possible owing to the local *C*_3_ symmetry of **3**, the interaction of the oxo ligand with the Na^+^ cation as well as the trans influence of the appended Co center weakens the Zr–O bonding in **3** compared to **2**.

**Fig. 4:**
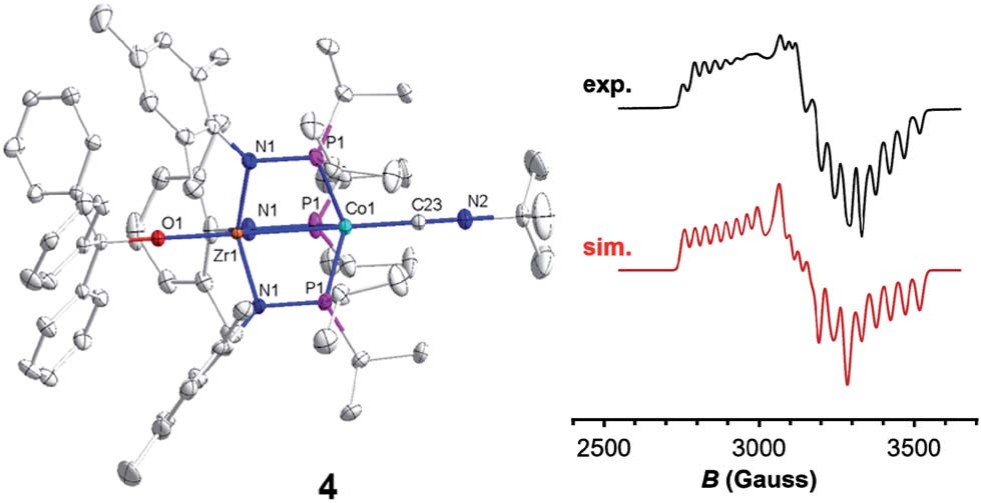
Displacement ellipsoid (50%) representations (left) and X-band EPR spectra (right, experimental: black, top and simulated: red, bottom) of **4** in fluorobenzene at 30 K (power attenuation = 30 dB, modulation amplitude = 10 G, modulation frequency = 100 kHz). Hydrogen atoms and solvate molecules have been omitted for clarity. Selected interatomic distances (Å) of **4**: Zr1–Co1, 2.7223(5); Zr1–O1, 1.987(2). EPR simulation parameters: *g* = 2.00, 2.11, and 2.33; *A* = 130, 109, and 82 MHz.

Trityl radical also served as an effective inner sphere reductant for oxo compound **2**. A reaction between **2** and half an equivalent of Gomberg's dimer (3-triphenylmethyl-6-diphenylmethylidene-1,4-cyclohexadiene), which dissociates into Ph_3_C˙ in solution,^46,47^ afforded Ph_3_COZr(MesNP^i^Pr_2_)_3_CoCN^*t*^Bu (**4**) after stirring for two hours in fluorobenzene (Scheme 1). The solid-state structure of **4** confirmed addition of the trityl radical to the Zr-bound oxo ligand to afford a new triphenyl methoxide functionality (Fig. 4). The paramagnetically shifted ^1^H chemical shift pattern of **4** is similar to the isoelectronic *S* = 1/2 compound **3**, in support of an overall one-electron reduction process from **2** to **4**. The C≡N stretch (2009 cm^−1^) in the solid-state ATR IR spectrum of **4** is at higher frequency than isoelectronic compound **3**, which can be attributed to the difference in overall charge between the two Zr^IV^/Co^0^ molecules; however, the *ν*(CN) of **4** remains significantly lower compared to Zr^IV^/Co^I^ oxo compound **2** (2114 cm^−1^).^28^

Since the poor solubility of compound **4** precluded measurement of its magnetic moment in solution, EPR spectroscopy was used to confirm its *S* = 1/2 spin state. The X-band EPR spectrum of **4** exhibited a rhombic signal with simulated *g* values of 2.00, 2.11, and 2.33 with an average *g* = 2.15 (Fig. 4), consistent with an *S* = 1/2 system. The eight-line hyperfine splitting pattern associated with each *g* tensor is attributed to coupling to the ^59^Co nucleus (*I* = 7/2, *A* = 130, 109, 82 MHz), and therefore spin localization on the Co center. The combined spectral evidence confirms that the reduction of **2** is cobalt-centered with the unpaired electron in both **3** and **4** residing on the d^9^ Co^0^ center.

**Scheme 2:**
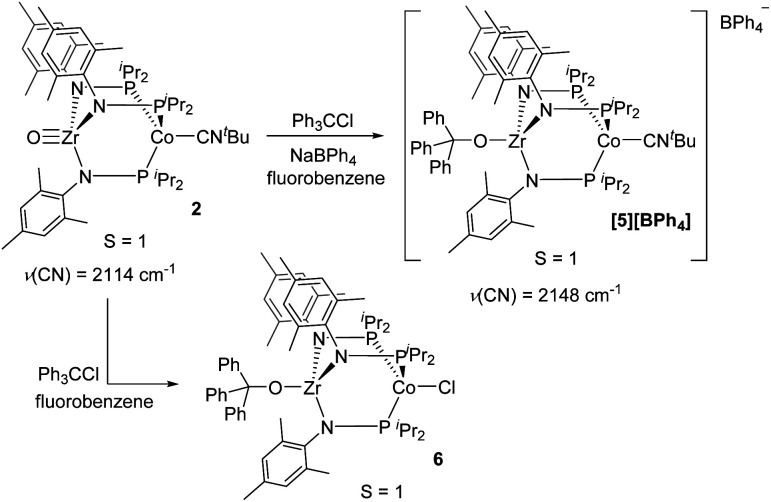
The reactivity of **2** with Ph_3_C^+^ reagents.

Although the potential of the Ph_3_C˙/Ph_3_C^+^ redox couple suggests that Ph_3_C˙ is not a sufficiently strong reductant to reduce **2**,^48^ the reaction is likely driven to completion by the strong affinity of the electrophilic Ph_3_C^+^ cation for the oxo fragment. To test this hypothesis, complex **2** was treated directly with trityl cation reagents. The reaction between **2** and [Ph_3_C][BPh_4_] (generated *in situ* from NaBPh_4_ and Ph_3_CCl) afforded the bright green compound [Ph_3_COZr(MesNP^i^Pr_2_)_3_CoCN^*t*^Bu][BPh_4_] (**[5][BPh4]**, Scheme 2). Compound **[5][BPh4]** is the *S* = 1 (*μ*_eff_ = 2.95 B.M.), one-electron oxidized, isostructural analogue of **4** with BPh_4_^−^ as the counteranion. The paramagnetically shifted ^1^H NMR chemical shift pattern of **[5][BPh4]** is consistent with other *C*_3_-symmetric Zr^IV^Co^I^ tris(phosphinoamide) species such as oxo precursor **2**.^28^ The infrared C≡N stretching frequency of **[5][BPh4]** (2148 cm^−1^) is significantly higher than the Zr^IV^/Co^0^ analogue **4** (2009 cm^−1^) owing to the higher oxidation state of the Co center. The *ν*(CN) is also considerably higher than that of isoelectronic **2** (2114 cm^−1^), which can be attributed to the overall cationic charge of **[5][BPh4]**.

**Fig. 5:**
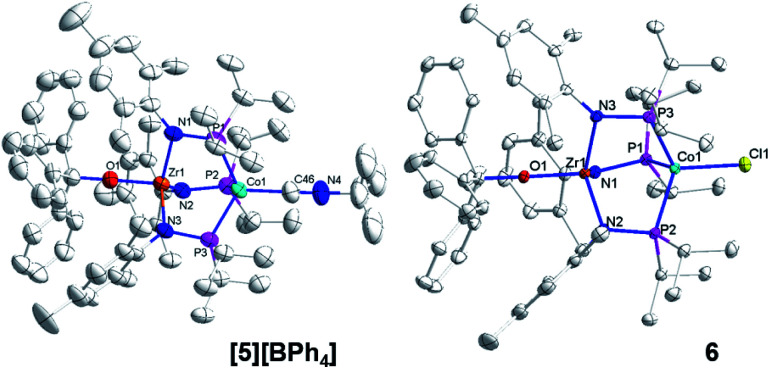
Displacement ellipsoid (50%) representations of **[5][BPh4]** and **6**. Hydrogen atoms, solvate molecules, and the BPh_4_^−^ counteranion of **[5][BPh4]** were omitted for clarity. Selected interatomic distances (Å): **[5][BPh4]**: Zr1–Co1, 2.9212(4); Zr1–O1, 1.9301(15). **6**: Zr1–Co1, 2.9005(3); Zr1–O1, 1.9558(8).

The addition of trityl cation to oxo compound **2** proceeds differently in the presence of a coordinating counteranion. A stoichiometric reaction between **2** and Ph_3_CCl led to formation of the neutral blue product Ph_3_COZr(MesNP^i^Pr_2_)_3_CoCl (**6**, Scheme 2). Under these conditions, the tightly bound isocyanide ligand was displaced by the chloride ligand (Fig. 5). The solution magnetic moment of **6** (*μ*_eff_ = 2.81 B.M.) is consistent with an *S* = 1 species isoelectronic to compounds **2** and **[5][BPh4]**.

Compounds **4**, **[5][BPh4]**, and **6** are structurally similar but with systematic changes in the intermetallic Zr–Co distances as a result of differences in the oxidation state of the cobalt center (Fig. 4 and 5). The Zr–Co distance (2.7223(5) Å) in the Zr^IV^/Co^0^ compound **4** is the shortest in the **4–6** series, and is in good agreement with the Zr–Co distances reported for similar RO-Zr(MesNP^i^Pr_2_)_3_Co–L species (R = H, alkyl, or aryl; L = CO, N_2_; range = 2.6324(3)–2.7616(6) Å).^25,39,45^ The Zr–Co distances in Zr^IV^/Co^I^ complexes **[5][BPh4]** and **6** (2.9212(4) Å and 2.9005(3) Å, respectively) are elongated by approximately 0.2 Å compared to **4** as a result of diminished dative Co → Zr interactions from the less electron-rich oxidized Co^I^ center. The Zr–O distance in **4** (1.987(2) Å) is slightly elongated compared to **[5][BPh4]** and **6** (1.9301(15) Å and 1.9558(8) Å, respectively), which may be reflective of a stronger *trans* influence from the more reduced Co center in **4**.

### Oxidation of O≡Zr(MesNP^i^Pr_2_)_3_CoCN^*t*^Bu (**2**)

**Scheme 3:**
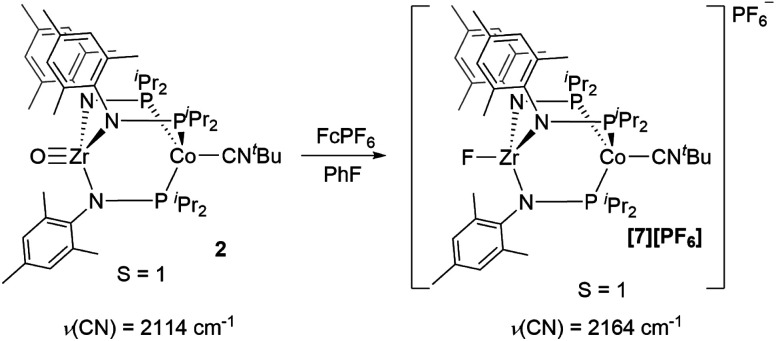
Chemical oxidation of **2** with FcPF_6_.

Next, we explored the chemical oxidation of **2**, which we anticipated to be immediately followed by another chemical step based on the irreversibility observed in its CV. The chemical oxidation of **2** by FcPF_6_ in fluorobenzene afforded the Zr-fluoride compound, [FZr(MesNP^i^Pr_2_)_3_CoCN^*t*^Bu][PF_6_] (**[7][PF6]**), as the major product as a result of fluoride abstraction from the PF_6_^−^ anion (Scheme 3). **[7][PF6]** is also generated when this reaction is performed THF, ruling out the fluorobenzene solvent as the source of the Zr-bound fluoride ion. The characterization data for **[7][PF6]**, including the *ν*(CN) stretch (2164 cm^−1^), the solution magnetic moment (*μ*_eff_ = 2.95 B.M.), and the range and pattern of chemical shifts in the ^1^H NMR spectrum, allowed its formulation as a *C*_3_-symmetric *S* = 1 Zr^IV^Co^I^ species. While the signals for the PF_6_^−^ counterion were easily observed in the ^31^P NMR (−145.7 ppm, septet) and ^19^F NMR (74.5 ppm, doublet) spectra, the ^19^F NMR signal for the Zr–F ligand was not observed due to its proximity to the paramagnetic Co center.

**Fig. 6:**
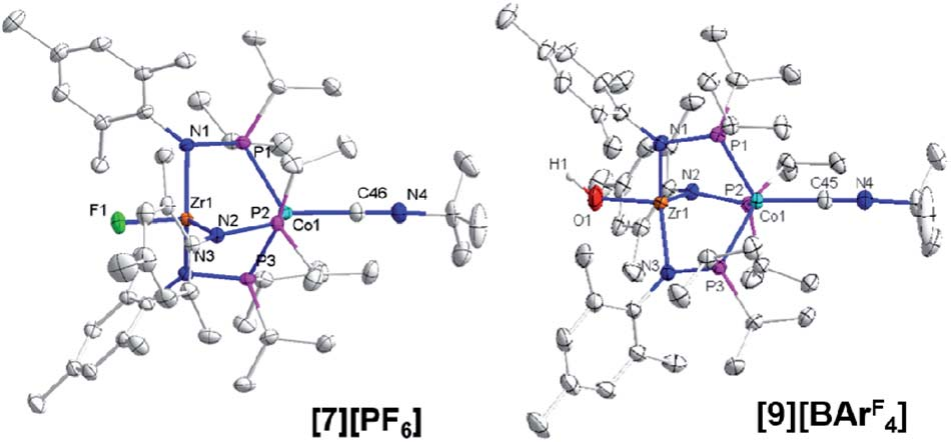
Displacement ellipsoid (50%) representations of (**[7][PF6]** and **[9][BArF4]**). Counteranions, solvate molecules, and hydrogen atoms except for the OH^−^ group on **[9][BArF4]** were omitted for clarity. Selected interatomic distances (Å): **[7][PF6]**: Zr1–Co1, 2.7498(2); Zr1–F1, 1.9319(8). **[9][BArF4]**: Zr1–Co1, 2.8396(3); Zr1–O1, 1.9209(13).

Single crystal X-ray diffraction confirmed the connectivity of **[7][PF6]** and revealed a Zr–Co interatomic distance of 2.7498(2) Å (Fig. 6), which is somewhat shorter than is typically observed for isoelectronic Zr^IV^/Co^I^ compounds with oxygen-based ligands bound to Zr (*e.g.* Ph_3_BOZr(MesNP^i^Pr_2_)_3_CoCN^*t*^Bu, Zr–Co: 2.9793(6) Å).^28^ This phenomenon is likely reflective of the more electronegative nature of the fluoride anion promoting a stronger Co → Zr dative interaction. On the other hand, the Zr–Co distance 2.7498(2) Å is longer compared to that of the isoelectronic precursor ClZr(MesNP^i^Pr_2_)_3_CoI (2.6280(5) Å),^49^ likely due to the Co → CN^*t*^Bu back-donation to weaken dative Co → Zr interactions. Oxidation of **2** with ferrocenium salts containing less reactive tetraarylborate anions (*e.g.* FcBPh_4_, FcBAr^F^_4_; BAr^F^_4_^−^ = tetrakis(3,5-bis(trifluoromethyl)phenyl)borate) led to mixtures of *S* = 1 products including both H-atom abstraction products (**[9][BR4]**, *vide infra*) and borane adducts R_3_BOZr(MesNP^i^Pr_2_)_3_CoCN^*t*^Bu^28^ that were identified based on comparison of crude ^1^H NMR spectra to previously characterized compounds.

**Fig. 7:**
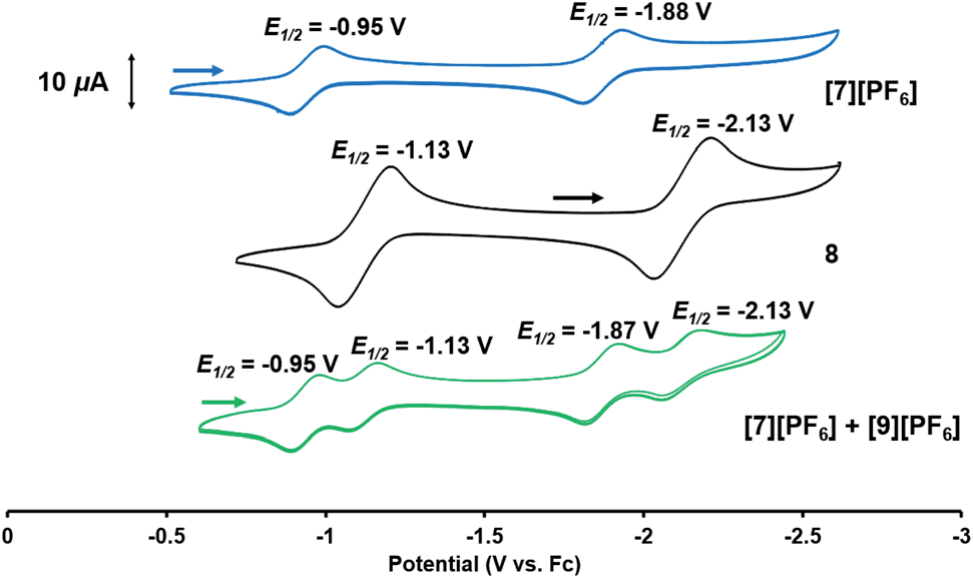
Cyclic voltammograms (in 0.3 M [^*n*^Bu_4_N][PF_6_] in THF; scan rate: 100 mV s^−1^) of compounds **[7][PF6]** (blue), **8** (black), and a mixture of **[7][PF6]** and **[9][PF6]** obtained from the reaction of **8** with FcPF_6_ (green).

Based on the formation of fluoride complex **[7][PF6]** upon oxidation with FcPF_6_, we initially hypothesized that the new reversible redox events that appeared at −1.17 V and −2.18 V in the CV of **2** after the irreversible oxidation step (Fig. 2) were attributed to rapid fluoride ion abstraction from the PF_6_^−^ ions in the electrolyte solution. To probe this hypothesis, the CV of **[7][PF6]** was collected (Fig. 7, blue). Two reversible cobalt-based reduction events were observed in the CV of **[7][PF6]** at −0.95 V and −1.88 V, which are significantly more positive and not consistent with the aforementioned features in the CV of **2**.

**Scheme 4:**
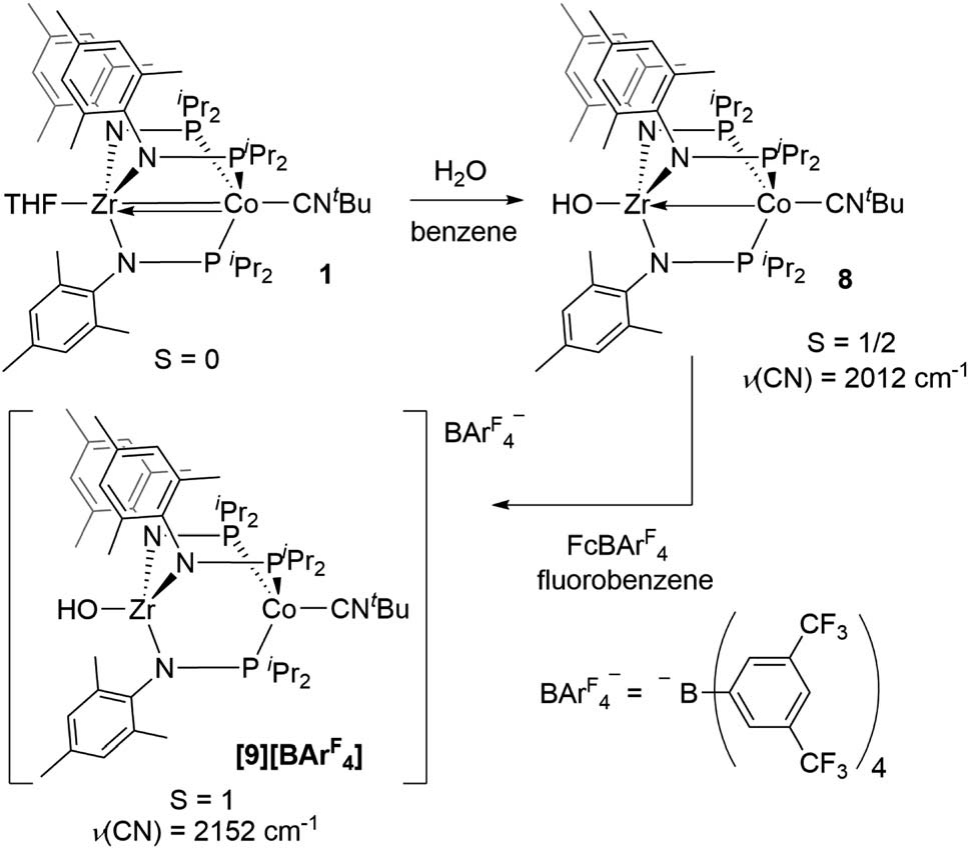
Synthesis of hydroxide compound **8** and its oxidation to form **9**.

An alternative hypothesis to explain the new redox features in the CV of **2** and identify the species generated upon electrochemical oxidation of **2** was therefore warranted. We posited that upon oxidation, the newly generated cation, [OZr(MesNP^i^Pr_2_)_3_CoCN^*t*^Bu]^+^**[2]+**, might have both nucleophilicity and oxidizing power, making it prone to H-atom abstraction from the THF solvent to form a hydroxide complex of general form [HOZr(MesNP^i^Pr_2_)_3_CoCN^*t*^Bu]^+^. To probe this hypothesis, the *S* = 1/2 hydroxide complex HOZr(MesNP^i^Pr_2_)_3_CoCN^*t*^Bu (**8**) was synthesized *via* treatment of **1** with stoichiometric water in a reaction analogous to those previously reported with N_2_- or CO-bound analogues of **1** (Scheme 4).^25,45^ The ^1^H NMR spectrum of **8** contains eight broad paramagnetic resonances, but also features a diagnostic Zr–O*H* signal at −45.7 ppm comparable to resonances observed for the isoelectronic hydroxide compounds HOZr(MesNP^i^Pr_2_)_3_CoN_2_ (−52.8 ppm) and HOZr(MesNP^i^Pr_2_)_3_CoCO (−40.6 ppm) (Fig. S21†).^25,45^ Single crystal X-ray diffraction confirmed the connectivity of **8**, including the hydroxide H atom, which was independently located in the difference map (Fig. S36†). The solid-state IR spectrum of **8** showed a *ν*(CN) at 2012 cm^−1^ and a *ν*(OH) at 3701 cm^−1^, and its solution magnetic moment was 1.64 B.M. Furthermore, the EPR spectrum of **8** features a rhombic signal centered at *g*_av_ = 2.14 (*g* = 2.00, 2.10, and 2.33) with a diagnostic eight line splitting pattern resulting from hyperfine coupling to the ^59^Co nucleus (^59^Co, *I* = 7/2, *A* = 132, 110, 100 MHz), further confirming the formulation of **8** as an *S* = 1/2 Zr^IV^/Co^0^ complex (Fig. S23†).

The redox properties of hydroxide complex **8** were then investigated for comparison with the unknown species generated upon the oxidation of **2**. As shown in Fig. 7 and S24,† the CV of **8** reveals two reversible redox events, namely a Co^0/−I^ reduction at −2.13 V and a Co^I/0^ oxidation at −1.13 V *vs.* Fc^+^/Fc. These features are therefore quite similar to those observed following oxidation of oxo complex **2** (−1.17 V and −2.18 V), consistent with the hypothesis that H-atom abstraction is the rapid chemical step that follows oxidation.

One-electron oxidation of complex **8** with FcBAr^F^_4_ afforded the cationic *S* = 1 hydroxide compound [HOZr(MesNP^i^Pr_2_)_3_CoCN^*t*^Bu][BAr^F^_4_] (**[9][BArF4]**). In addition to the two signals for BAr^F^_4_^−^ anion, the ^1^H NMR spectrum of **[9][BArF4]** contains eight broad and paramagnetically shifted resonances, including a signal at −60.0 ppm corresponding to the Zr-bound hydroxide proton. The measured solution magnetic moment of **[9][BArF4]** (*μ*_eff_ = 2.59 B.M.) is consistent with two unpaired electrons. The solid-state structure of **[9][BArF4]** confirmed its connectivity and revealed an elongated Zr–Co interatomic distance compared to **8** (2.8396(3) Å *vs.* 2.6902(18) Å, Fig. 6). As expected, the redox features in the CV of cation **[9]+** are superimposable with those of neutral complex **8** (Fig. S7†), but the open circuit potential of **[9]+** is more positive (−0.87 V *vs.* −1.27 V *vs.* Fc^+^/Fc).

**Scheme 5:**
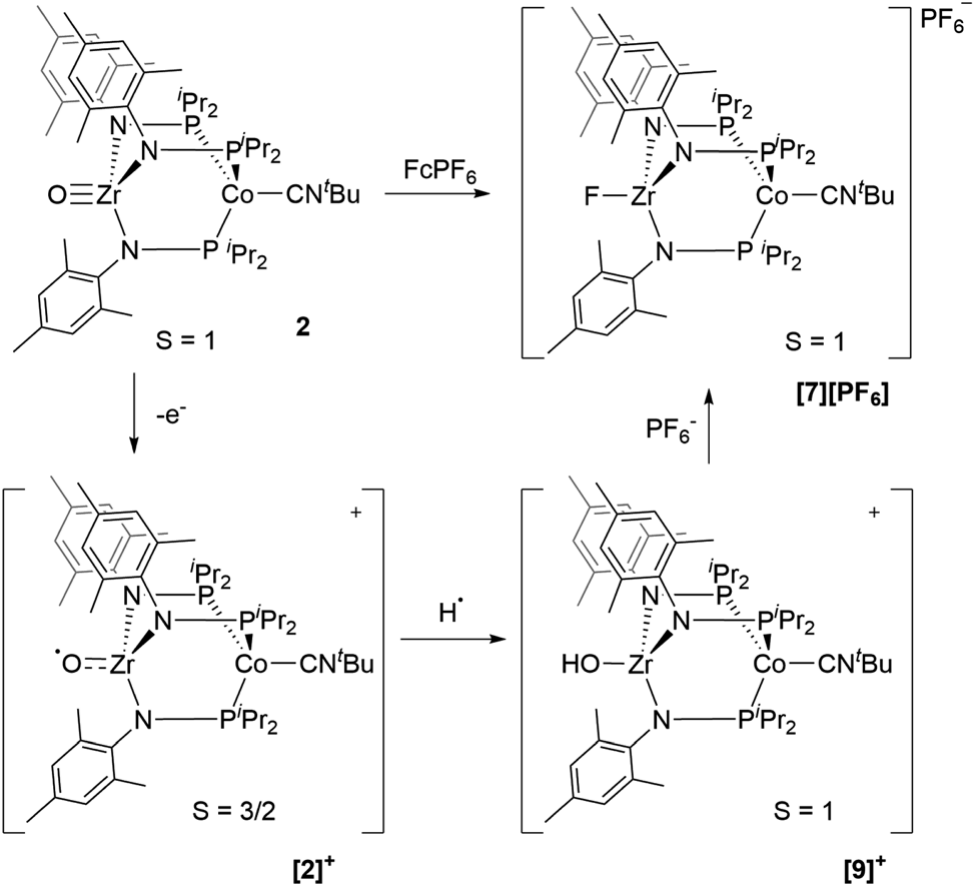
Proposed transformation from oxo compound **2** to fluoride compound **[7][PF6]** based on experimental and computational study.

Thus, we can definitively attribute the new signals observed in the CV of **2** following its oxidation to a cationic hydroxide species, **[9]+**, generated by an H-atom abstraction step by the transiently generated cationic oxo species [OZr(MesNP^i^Pr_2_)_3_CoCN^*t*^Bu]^+^ (**[2]+**). The proclivity of this oxidized compound to abstract H-atoms is unique to this heterobimetallic species, as a monometallic Zr oxo complex would not be sufficiently oxidizing to participate in such a proton-coupled electron transfer step. A remaining question, however, is why a cationic hydroxide complex such as **[9]+** is not isolated upon oxidation of **2** with FcPF_6_ or, conversely, why the fluoride cation **[7]+** is not generated under electrochemical oxidation conditions. Indeed, the cleavage of a strong Zr–O bond to form fluoride complex **[7]+** was initially quite surprising. In a control reaction, no reaction was observed between oxo complex **2** and [^*n*^Bu_4_N][PF_6_], revealing that fluoride ions are not abstracted from PF_6_^−^ under non-oxidative conditions. Furthermore, solvent effects could be ruled out as oxidation of **2** with FcPF_6_ in THF rather than fluorobenzene did not lead to detectable formation of **[9]+**. We therefore propose that one-electron oxidation to generate **[2]+** is immediately followed by rapid H-atom abstraction to form **[9]+** under both chemical and electrochemical oxidation conditions (Scheme 5). While the hydroxide cation **[9]+** persists long enough on the electrochemical time scale to be observed in the CV scans, the longer time scale of the chemical oxidation reaction with FcPF_6_ permits a fluoride ion from the PF_6_^−^ anion to displace the Zr-bound hydroxide ligand. Consistent with this hypothesis, the reaction of **[9]+** with FcPF_6_ results in quantitative conversion to the fluoride species **[7]+**. On the other hand, treating the *S* = 1/2 hydroxide compound **8** with stoichiometric [^*n*^Bu_4_N][PF_6_] did not lead to any reaction, suggesting the important role of oxidation in OH^−^/F^−^ exchange to generate the fluoride compound **[7]+**. Additionally, a stoichiometric reaction between the hydroxide compound **8** and FcPF_6_ affords a mixture of fluoride complex **[7]+** and hydroxide complex **[9]+**, and a CV of this mixture highlights the distinguishable differences between their redox potentials (Fig. 7, green voltammogram).

**Fig. 8:**
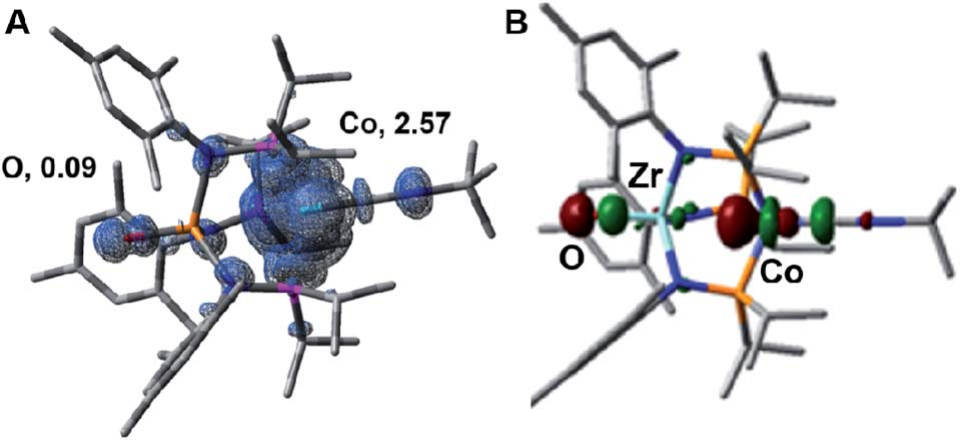
(A) Calculated unpaired spin density surface of **[2]+** in the predicted quartet state, with Mulliken atomic spin densities on the cobalt and oxygen atoms indicated. (B) Calculated highest doubly occupied molecular orbital of **2**.

The electronic structure of the oxidized species **[2]+** was investigated computationally using density functional theory (DFT) to better understand the instability of **[2]+** and the origin of its H-atom abstraction reactivity. DFT calculations were performed on two possible spin states of **[2]+**, revealing the quartet to be 7.1 kcal mol^−1^ more stable than the doublet spin state. In the quartet configuration, the unpaired electrons in **[2]+** were found to be predominately localized on the cobalt center, with a small but not negligible amount of spin density residing on the Zr-bound oxo ligand (Fig. 8A). The unpaired spin density on the oxo ligand results from a three-center four-electron bonding interaction involving the O, Zr, and Co atoms. As a result, the highest doubly occupied molecular orbital of **2** has both Co and O character (Fig. 8B), leading to unpaired electron density on the oxygen atom of **[2]+** upon oxidation of **2**. Since the Zr-bound oxygen atom is the least sterically protected site with significant spin density, the computational results provide an excellent explanation for the observed H-atom abstraction process that occurs upon oxidation of **2**.

## Conclusions

In summary, the reactivity of the terminal oxo heterobimetallic Zr/Co compound **2** was explored, taking advantage of the steric protection of the mesityl ligand substituents that prevent oxo ligand bridging modes. In contrast to a monometallic Zr^IV^ oxo compound, **2** exhibited redox chemistry that was enabled by the pendent Co center, leading to the synthesis and isolation of a series of bimetallic Zr/Co compounds **3–9**. Although no direct metal–metal bond is present in **2**, outer-sphere redox changes mediated by the Co center are shown to work in concert with the inner-sphere nucleophilic reactivity of the reactive Zr≡O moiety. Both experimental and computational studies suggested that one-electron oxidation of **2** affords a transient oxidized species, **[2]+**, containing a nucleophilic Zr oxo fragment and a tethered oxidizing cobalt center that work in concert to facilitate H-atom abstraction. This study represents another mechanism by which early/late heterobimetallic compounds can initiate unique reactivity *via* participation of two metals that play distinct roles. In this case, the late metal (Co) serves as a redox-active metalloligand to mediate redox reactions at a d^0^ Zr^IV^-oxo fragment.

## Conflicts of interest

There are no conflicts to declare.

## Supplementary Material

SC-011-D0SC04229C-s001

SC-011-D0SC04229C-s002
