# Supplementary material for: Redox chemistry and H-atom abstraction reactivity of a terminal zirconium(iv) oxo compound mediated by an appended cobalt(i) center
Source: Chem Sci. 2020 Sep 4;11(39):10729–36. doi: 10.1039/d0sc04229c (PMC8162367; doi:10.1039/d0sc04229c)
Supplement: SC-011-D0SC04229C-s001 [file SC-011-D0SC04229C-s001.pdf]

Electronic Supplementary Material (ESI) for Chemical Science.
This journal is © The Royal Society of Chemistry 2021

SUPPORTING INFORMATION

Redox chemistry and oxyl radical behavior of a terminal zirconium(IV) oxo compound mediated by an appended cobalt(I) center

Hongtu Zhang,[a] Gillian P. Hatzis,[a] Diane A. Dickie,[b] Curtis E. Moore,[a] and Christine M. Thomas*[a]

Department of Chemistry and Biochemistry, The Ohio State University, 100 W. 18th Ave, Columbus OH 43210 USA

Department of Chemistry, University of Virginia, 409 McCormick Road, PO Box 400319, Charlottesville, VA 22904

CONTENTS

General consideration 5

Synthesis of [(μ-Na)OZr(MesNP^{i}Pr$_2$)$_3$CoCN^{t}Bu]$_2$ (**3**) 6

Figure S1. ^{1}H NMR spectrum (400 MHz, C_6D_6) of [(μ-Na)OZr(MesNP^{i}Pr$_2$)$_3$CoCN^{t}Bu]$_2$ (**3**). Peaks corresponding to residual solvents, in this case C_6D_5H and Et_2O, are labelled. 7

Figure S2. Solid state (ATR) IR spectrum of [(μ-Na)OZr(MesNP^{i}Pr$_2$)$_3$CoCN^{t}Bu]$_2$ (**3**). 8

Synthesis of Ph$_3$COZr(MesNP^{i}Pr$_2$)$_3$CoCN^{t}Bu (**4**) 9

Figure S3. ^{1}H NMR spectrum (400 MHz, C_6D_6) of Ph$_3$COZr(MesNP^{i}Pr$_2$)$_3$CoCN^{t}Bu (**4**). Peaks corresponding to residual solvents, in this case C_6D_5H, THF, Et_2O, and pentane, are labelled....10

Figure S4. Solid state (ATR) IR spectrum of Ph$_3$COZr(MesNP^{i}Pr$_2$)$_3$CoCN^{t}Bu (**4**)....................10

Figure S5. Positive ion ESI-MS spectrum of **4** in PhF. One predominant Zr/Co species was observed in full spectrum: [Ph$_3$COZr(MesNP^{i}Pr$_2$)$_3$CoCN^{t}Bu]$^+$ (*m/z* = 1241.5404). Isotopic peaks are consistent between predicted (bottom left) and experimental (bottom right) spectra for Zr/Co species. 11

Figure S6. Experimental (black) and simulated (red) X-band EPR spectra of compound **4** showing spin localization on ^{59}Co (I = 7/2). Spectrum was obtained in frozen fluorobenzene at 30 K with two scans (power attenuation = 30 dB, modulation amplitude = 10 G, modulation frequency = 100 kHz). Simulation parameters: g = 2.00, 2.11, and 2.33; A = 130, 109, and 82 MHz. 12

Synthesis of [Ph$_3$COZr(MesNP^{i}Pr$_2$)$_3$CoCN^{t}Bu][BPh$_4$] (**[5][BPh$_4$]**). 13

Figure S7. ^{1}H NMR spectrum (400 MHz, C_6D_6) of [$Ph_3COZr(MesNP^{i}Pr_2)_3CoCN^{t}Bu$][$BPh_4$] (**[5][BPh$_4$]**). Peaks corresponding to residual solvents, in this case $CDHCl_2$ and Et_2O, are labelled. 14

Figure S8. ^{11}B NMR spectrum (192 MHz, C_6D_6) of [$Ph_3COZr(MesNP^{i}Pr_2)_3CoCN^{t}Bu$][$BPh_4$] (**[5][BPh$_4$]**). 14

Figure S9. Solid state (ATR) IR spectrum of [$Ph_3COZr(MesNP^{i}Pr_2)_3CoCN^{t}Bu$][$BPh_4$] (**[5][BPh$_4$]**). 15

Figure S10. Positive ion ESI-MS spectrum of **[5][BPh$_4$]** in PhF. Two predominant Zr/Co species were observed in full spectrum: [$Ph_3COZr(MesNP^{i}Pr_2)_3CoCN^{t}Bu$]$^{+}$ (m/z = 1241.5271) and [$Ph_3COZr(MesNP^{i}Pr_2)_3Co$]$^{+}$ (m/z = 1158.4552). Isotopic peaks are consistent between predicted (bottom left) and experimental (bottom right) spectra for Zr/Co species. 15

Figure S11. Negative ion ESI-MS spectrum of **[5][BPh$_4$]** in PhF. One predominant species was observed in full spectrum: [BPh_4]$^{-}$ (m/z = 319.1667). Isotopic peaks are consistent between predicted (bottom left) and experimental (bottom right) spectra. 16

Synthesis of $Ph_3COZr(MesNP^{i}Pr_2)_3CoCl$ (**6**) 17

Figure S12. ^{1}H NMR spectrum (400 MHz, C_6D_6) of $Ph_3COZr(MesNP^{i}Pr_2)_3CoCl$ (**6**). Peaks corresponding to residual solvents, in this case $CDHCl_2$, and Et_2O, are labelled. 18

Figure S13. Solid state (ATR) IR spectrum of $Ph_3COZr(MesNP^{i}Pr_2)_3CoCl$ (**6**). 18

Figure S14. Positive ion ESI-MS spectrum of **6** in PhF. One predominant Zr/Co species was observed in the full spectrum: [$Ph_3COZr(MesNP^{i}Pr_2)_3CoCl$]$^{+}$ (m/z = 1193.4315). Isotopic peaks are consistent between predicted (bottom left) and experimental (bottom right) spectra for Zr/Co species. 19

Synthesis of [$FZr(MesNP^{i}Pr_2)_3CoCN^{t}Bu$][$PF_6$] (**[7][PF$_6$]**). 20

Figure S15. ^{1}H NMR spectrum (400 MHz, CD_2Cl_2) of [$FZr(MesNP^{i}Pr_2)_3CoCN^{t}Bu$][$PF_6$] (**[7][PF$_6$]**). Peaks corresponding to residual solvents, in this case $CDHCl_2$, Et_2O, THF, and PhF, are labelled. 21

Figure S16. ^{31}P{^{1}H} NMR spectrum (162 MHz, CD_2Cl_2) of [$FZr(MesNP^{i}Pr_2)_3CoCN^{t}Bu$][$PF_6$] (**[7][PF$_6$]**). 21

Figure S17. ^{19}F NMR spectrum (376 MHz, CD_2Cl_2) of [$FZr(MesNP^{i}Pr_2)_3CoCN^{t}Bu$][$PF_6$] (**[7][PF$_6$]**). 22

Figure S18. Solid state (ATR) IR spectrum of [$FZr(MesNP^{i}Pr_2)_3CoCN^{t}Bu$][$PF_6$] (**[7][PF$_6$]**). 22

Figure S19. Positive ion ESI-MS spectrum of **[7][PF$_6$]** in PhF. Two predominant species containing Zr/Co were observed in the full spectrum: [FZr(MesNP^{i}Pr$_2$)$_3$CoCN^{t}Bu]$^+$ (*m/z* = 1001.4374) and [FZr(MesNP^{i}Pr$_2$)$_3$Co]$^+$ (*m/z* = 918.3540). Isotopic peaks are consistent between predicted (bottom left) and experimental (bottom right) spectra for Zr/Co species. 23

Figure S20. Full cyclic voltammogram (CV) of **[7][PF$_6$]** vs. Fc/Fc$^+$. CV was collected in 0.3 M [^{n}Bu$_4$N][PF$_6$] THF solution, scanning cathodically starting from the open circuit potential with a scan rate of 100 mV/s. 23

Synthesis of HOZr(MesNP^{i}Pr$_2$)$_3$CoCN^{t}Bu (**8**) 24

Figure S21. ^{1}H NMR spectrum (400 MHz, C_6D_6) of HOZr(MesNP^{i}Pr$_2$)$_3$CoCN^{t}Bu (**8**). Peaks corresponding to residual solvents, in this case C_6D_5H, THF, and pentane, are labelled. 25

Figure S22. Solid state (ATR) IR spectrum of HOZr(MesNP^{i}Pr$_2$)$_3$CoCN^{t}Bu (**8**). 25

Figure S23. Experimental (black) and simulated (red) X-band EPR spectra of compound **8** showing spin localization on ^{59}Co (I = 7/2). Spectrum was obtained in frozen fluorobenzene at 30 K with two scans (power attenuation = 30 dB, modulation amplitude = 10 G, modulation frequency = 100 kHz.). Simulation parameters: g = 2.00, 2.10, and 2.33; A = 132, 110, and 100 MHz. 26

Figure S24. Full cyclic voltammogram (CV) of **8** vs. Fc/Fc$^+$. CV was collected in 0.3 M [^{n}Bu$_4$N][PF$_6$] THF solution, scanning cathodically starting from open circuit potential with a scan rate of 100 mV/s. 27

Synthesis of [HOZr(MesNP^{i}Pr$_2$)$_3$CoCN^{t}Bu][BAr$^F_4$] (**[9][BAr$^F_4$]**) 28

Figure S25. ^{1}H NMR spectrum (400 MHz, CD_2Cl_2) of [HOZr(MesNP^{i}Pr$_2$)$_3$CoCN^{t}Bu][BAr$^F_4$] (**[9][BAr$^F_4$]**). Peaks corresponding to residual solvents, in this case $CDHCl_2$ and pentane, are labelled. 29

Figure S26. ^{11}B NMR spectrum (192 MHz, CD_2Cl_2) of [HOZr(MesNP^{i}Pr$_2$)$_3$CoCN^{t}Bu][BAr$^F_4$] (**[9][BAr$^F_4$]**). 29

Figure S27. ^{19}F NMR spectrum (565 MHz, CD_2Cl_2) of [HOZr(MesNP^{i}Pr$_2$)$_3$CoCN^{t}Bu][BAr$^F_4$] (**[9][BAr$^F_4$]**). 30

Figure S28. Solid state (ATR) IR spectrum of [HOZr(MesNP^{i}Pr$_2$)$_3$CoCN^{t}Bu][BAr$^F_4$] (**[9][BAr$^F_4$]**). 30

Figure S29. Positive ion ESI-MS spectrum of **[9][BAr$^F_4$]** in PhF. Two predominant species containing Zr/Co were observed in the full spectrum: [HOZr(MesNP^{i}Pr$_2$)$_3$CoCN^{t}Bu]$^+$ (*m/z* = 999.4539) and [HOZr(MesNP^{i}Pr$_2$)$_3$Co]$^+$ (*m/z* = 916.3691). Isotopic peaks are consistent between predicted (bottom left) and experimental (bottom right) spectra for Zr/Co species. 31

Figure S30. Negative ion ESI-MS spectrum of **[9][BAr^F$_4$]** in PhF. One predominant species was observed in the full spectrum: [BAr^F$_4$]$^-$ (*m/z* = 863.0556). Isotopic peaks are consistent between predicted (bottom left) and experimental (bottom right) spectra. 31

Table S1. X-ray diffraction experimental details for [(μ-Na)OZr(MesNP^{i}Pr$_2$)$_3$CoCN^{t}Bu]$_2$ (**3**), Ph$_3$COZr(MesNP^{i}Pr$_2$)$_3$CoCN^{t}Bu (**4**), and [Ph$_3$COZr(MesNP^{i}Pr$_2$)$_3$CoCN^{t}Bu][BPh$_4$] (**[5][BPh$_4$]**). 32

Table S2. X-ray diffraction experimental details for Ph$_3$COZr(MesNP^{i}Pr$_2$)$_3$CoCl (**6**), [FZr(MesNP^{i}Pr$_2$)$_3$CoCN^{t}Bu][PF$_6$] (**[7][PF$_6$]**), and HOZr(MesNP^{i}Pr$_2$)$_3$CoCN^{t}Bu (**8**). 33

Table S3. X-ray diffraction experimental details for [HOZr(MesNP^{i}Pr$_2$)$_3$CoCN^{t}Bu][BAr^F$_4$] (**[9][BAr^F$_4$]**). 34

Figure S31. Fully labelled displacement ellipsoid (50%) diagram of **3**•2C$_5$H$_{12}$. The sodium was disordered across three positions with combined occupancy that converged at 81%, 12% and 7 %. Hydrogens have been omitted for clarity. 35

Figure S32. Fully labelled displacement ellipsoid (50%) diagram of **4**•3C$_6$H$_5$F. The whole molecule and the fluorobenzene solvent molecule were found to be fully disordered; disordered fluorobenzene solvate molecules, and hydrogens are omitted for clarity. 36

Figure S33. Fully labelled displacement ellipsoid (50%) diagram of **[5][BPh$_4$]**. The ^{t}BuNC group on **[5]**$^+$ and a phenyl group on [BPh$_4$]$^-$ were found to be disordered. Hydrogens have been omitted for clarity. 37

Figure S34. Fully labelled displacement ellipsoid (50%) diagram of **6**•2.5C$_6$H$_5$F. Hydrogens have been omitted for clarity. 38

Figure S35. Fully labelled displacement ellipsoid (50%) diagram of **[7][PF$_6$]**•2C$_6$H$_5$F. [PF$_6$] was found to be disordered. Hydrogens have been omitted for clarity. 39

Figure S36. Fully labelled displacement ellipsoid diagram of **8**. Hydrogens except for the hydroxide H have been omitted for clarity. 40

Figure S37. Fully labelled displacement ellipsoid diagram of **[9][BAr^F$_4$]**, with occupational disorder modeled of hydroxide trace iodide (~1%). Hydrogens except for the hydroxide H have been omitted for clarity. 41

Computational Details 42

Table S4. Calculated XYZ coordinates for all atoms in [**2**]$^+$ in the S = 3/2 state. 42

Table S5. Calculated XYZ coordinates for all atoms in [**2**]$^+$ in the S = 1/2 state. 46

Table S6. Calculated XYZ coordinates for all atoms in **2**... 50

Figure S38. ^{19}F and $^{31}P\{^1H\}$ NMR spectra of the reaction mixture between **2** and $FcPF_6$........54

References...55

General consideration

All manipulations were carried out under an inert atmosphere using a nitrogen-filled glovebox or standard Schlenk techniques unless otherwise noted. All glassware was oven-dried prior to use. Diethyl ether was obtained as HPLC grade without inhibitors. All protio solvents were degassed by sparging with ultra-high purity argon and dried *via* passage through columns of drying agents using a Seca solvent purification system from Pure Process Technologies. Benzene-d_6 and dichloromethane-d_2 were dried with CaH_2 overnight, vacuum transferred to an oven-dried vessel, and freeze-pump-thawed, then stored over 4 Å molecular sieves. $(THF)Zr(MesNP^iPr_2)_3CoN_2$,[1] $(THF)Zr(MesNP^iPr_2)_3CoCN^tBu$,[2] $FcBAr^F_4$ (BAr^F_4 = tetrakis[(3,5-trifluoromethyl)phenyl]borate),[3,4] and Gomberg's dimer[5,6] were synthesized following literature procedures. All other chemicals were purchased from commercial vendors and used without further purification. NMR spectra were recorded on a Varian Inova 400 MHz, a Bruker DPX 400 MHz (^{19}F: 376 MHz, ^{11}B: 128 MHz, ^{31}P: 162 MHz), and a Bruker 600 Avance III HD (^{19}F: 565 MHz, ^{11}B: 192 MHz, ^{31}P: 243 MHz) instrument. For ^1H NMR spectra, the solvent resonance was referenced as an internal standard. For ^{11}B NMR spectra, boron trifluoride etherate ($BF_3{\cdot}Et_2O$) was used as the external standard (0 ppm). For ^{19}F NMR spectra, trifluoroacetic acid (CF_3COOH) was used as the external standard (-76.55 ppm). Solution infrared spectra were recorded on a Bruker TENSOR II spectrometer controlled by OPUS software. Solid-state attenuated total reflection (ATR) infrared spectra were recorded on a Bruker ALPHA II spectrometer controlled by OPUS software. Room-temperature UV-vis spectra were recorded on

a Cary 5000 UV-vis-NIR spectrophotometer using Cary WinUV software. GC-MS data were collected on an Agilent 7890B/5977B GC/MSD with a split to both FID and mass spectrometer detectors using He as the carrier gas. Solution magnetic moments were measured using Evans' method and are reported without taking into account any diamagnetic contributions (Pascal's constants were not used).[7,8] EPR spectra were collected at 30 K on a Bruker EMXplus spectrometer that operates at X-band frequencies of 9.4 GHz, and simulated using EasySpin for MATLAB.[9] High-resolution ESI-MS data were analyzed on a Bruker micrOTOF (Bruker, Billerica, MA) with an ESI source. Cyclic voltammetry experiments were carried out in a nitrogen-filled glovebox using a CHI 620E potentiostat (CH instruments Inc., Austin TX), using 0.3 M [$^{n}Bu_4N$][PF_6] electrolyte solution in THF. A glassy carbon electrode and platinum wire were used as the working and auxiliary electrodes, respectively. The reference electrode was $Ag/AgNO_3$ in THF.

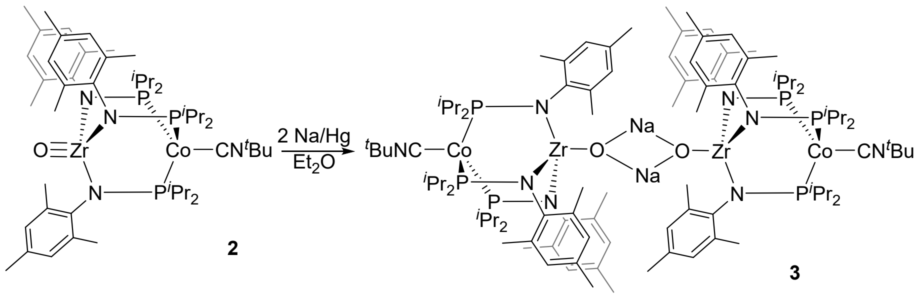

Synthesis of [(μ-Na)OZr(MesNP^{i}Pr$_2$)$_3$CoCN^{t}Bu]$_2$ (**3**)

Oxo compound **2** (91 mg, 0.091 mmol) was suspended in THF (2 mL) and briefly stirred. The resulting bright green suspension was added into a stirring THF (2 mL) suspension of 0.5% Na/Hg amalgam (4.2 mg Na, 0.183 mmol, 0.84 g Hg). The reaction mixture gradually became homogeneous and turned yellow and then orange in the first hour. The reaction mixture was allowed to stir at room temperature for 16 h. The clear orange supernatant was filtered through a

plug of Celite and concentrated under vacuum. Pentane was diffused into this concentrated solution at room temperature to afford yellow crystalline product. Yield: 27.5 mg, 29.6 %. Single crystals suitable for X-ray diffraction were chosen from those grown in this manner. ^{1}H NMR (400 MHz, C_6D_6): δ 7.73, 6.45, 5.10, 3.40 (br), 2.52, -2.10 (br). The P-C*H*$(CH_3)_2$ signal was not observed owing to its close proximity to the paramagnetic Co ion. Evans' method (μ_{eff}, C_6D_6): 1.88 B.M. UV-vis (C_6H_6, λ(nm) (ε, $M^{-1}cm^{-1}$)): 372 (4.8×10^3, sh). ATR IR: 1930 cm^{-1} ($\nu_{C\equiv N}$), 1952 cm^{-1} ($\nu_{C\equiv N}$). Owing to reactivity of **3** with air, moisture, and protic solvents, satisfactory elemental analysis and/or high-resolution ESI-MS data could not be obtained.

Figure S1. ^{1}H NMR spectrum (400 MHz, C_6D_6) of [(μ-Na)OZr(MesNP^{i}Pr$_2$)$_3$CoCN^{t}Bu]$_2$ (**3**). Peaks corresponding to residual solvents, in this case C_6D_5H and Et_2O, are labelled.

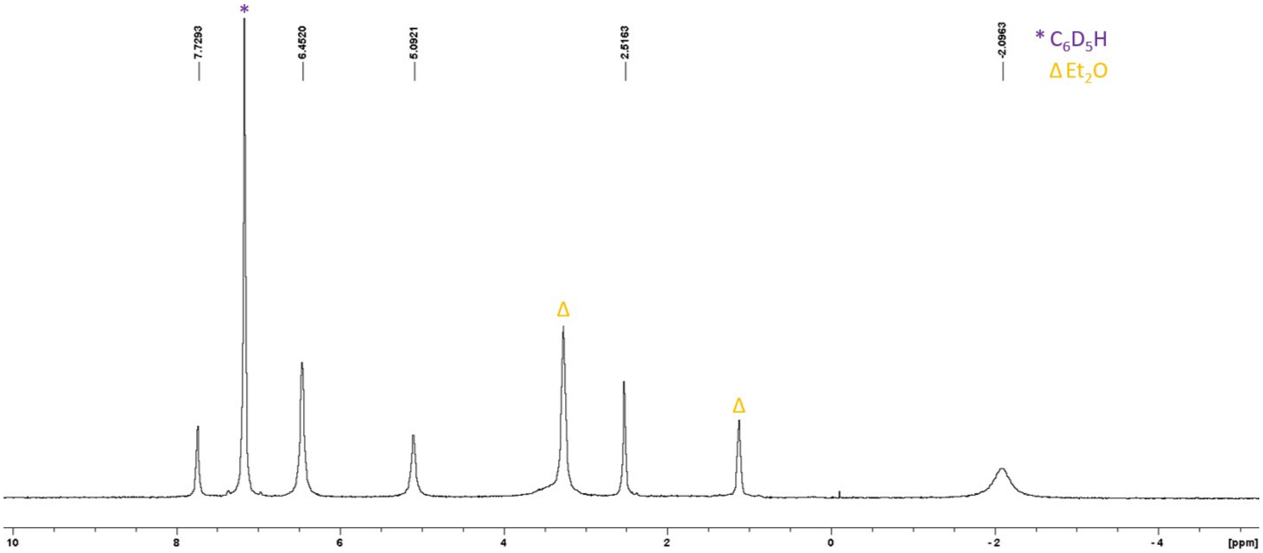

Figure S2. Solid state (ATR) IR spectrum of [(μ-Na)OZr(MesNP^{i}Pr$_2$)$_3$CoCN^{t}Bu]$_2$ (**3**).

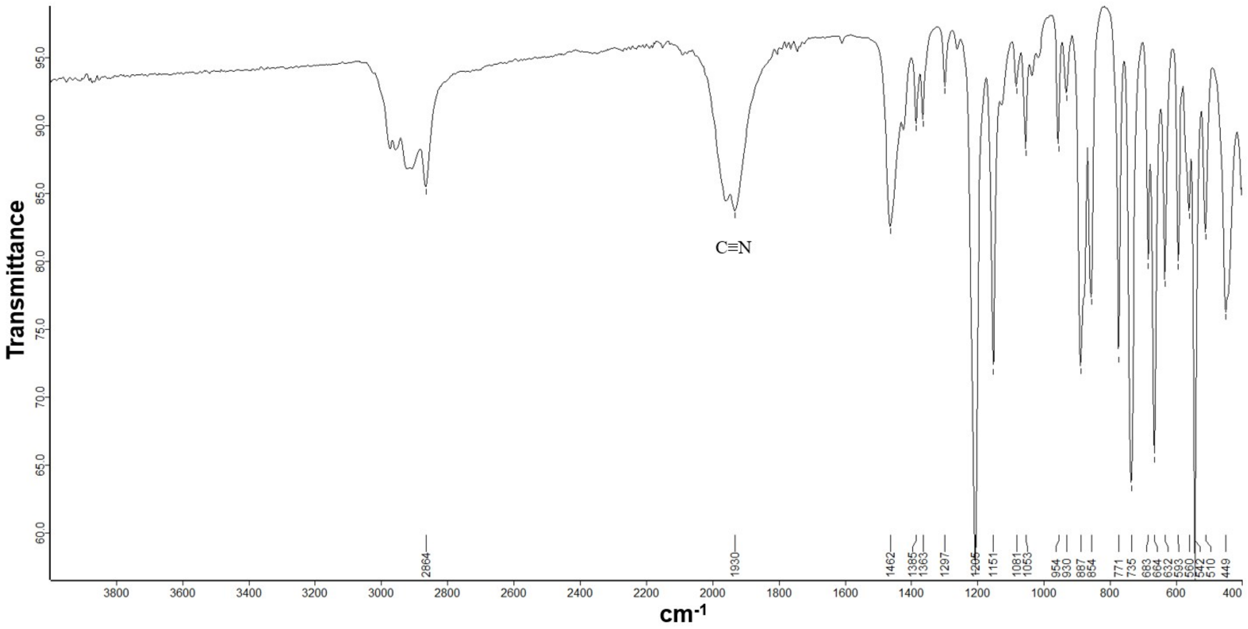

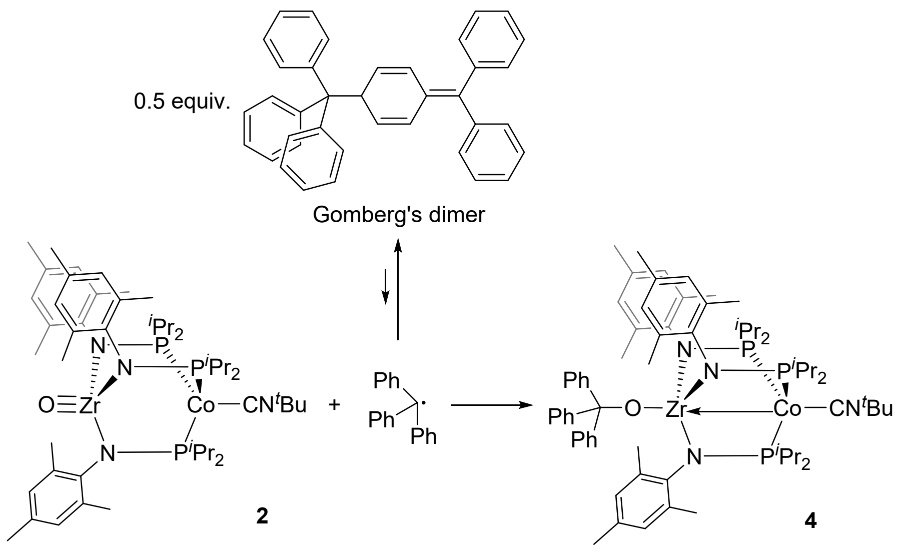

2 **4**

Synthesis of $Ph_3COZr(MesNP^{i}Pr_2)_3CoCN^{t}Bu$ (**4**)

Oxo compound **2** (74.5 mg, 0.0745 mmol) was added to fluorobenzene (1 mL) and stirred for 2 minutes to form a cloudy bright green suspension. Gomberg's dimer (18.1 mg, 0.0372 mmol) was dissolved in fluorobenzene (1 mL) to produce a light-yellow solution that was added into the stirring suspension of **2**. After approximately 15 min of stirring, the suspension turned from green to yellow, and the reaction was allowed to stir for 2 h to ensure reaction completion. The cloudy fluorobenzene suspension was filtered to collect the yellow solid product, which was washed with fluorobenzene (0.5 mL). The product was then extracted into diethyl ether (6 mL) and filtered through a plug of glass microfiber filter paper, and the solvent was evaporated from the filtrate under vacuum to obtain pure solid product. Yield: 59.9 mg, 64.7%. A single crystal suitable for X-ray diffraction was grown from a saturated solution of **4** in 2:1 diethyl ether:fluorobenzene stored at -30 °C. ^{1}H NMR (400 MHz, C_6D_6): δ 7.63, 7.52, 7.37, 6.71, 4.89, 2.57 (br), 2.42, 2.07, -1.70 (br). Evans' method (μ_{eff}, C_6D_6): 1.73 B.M. UV-vis (C_6H_5F, λ(nm) (ε, $M^{-1}cm^{-1}$)): 423 (1.2×10^{3}, sh), 360 (4.1×10^{3}, sh). ATR IR: 2009 cm^{-1} ($\nu_{C\equiv N}$). ESI-HRMS (C_6H_5F, positive mode): Calcd *m/z* for $[\mathbf{4}]^{+}$: 1241.5411. Found: 1241.5404.

Figure S3. ^{1}H NMR spectrum (400 MHz, C_6D_6) of $Ph_3COZr(MesNP^iPr_2)_3CoCN^tBu$ (**4**). Peaks corresponding to residual solvents, in this case C_6D_5H, THF, Et_2O, and pentane, are labelled.

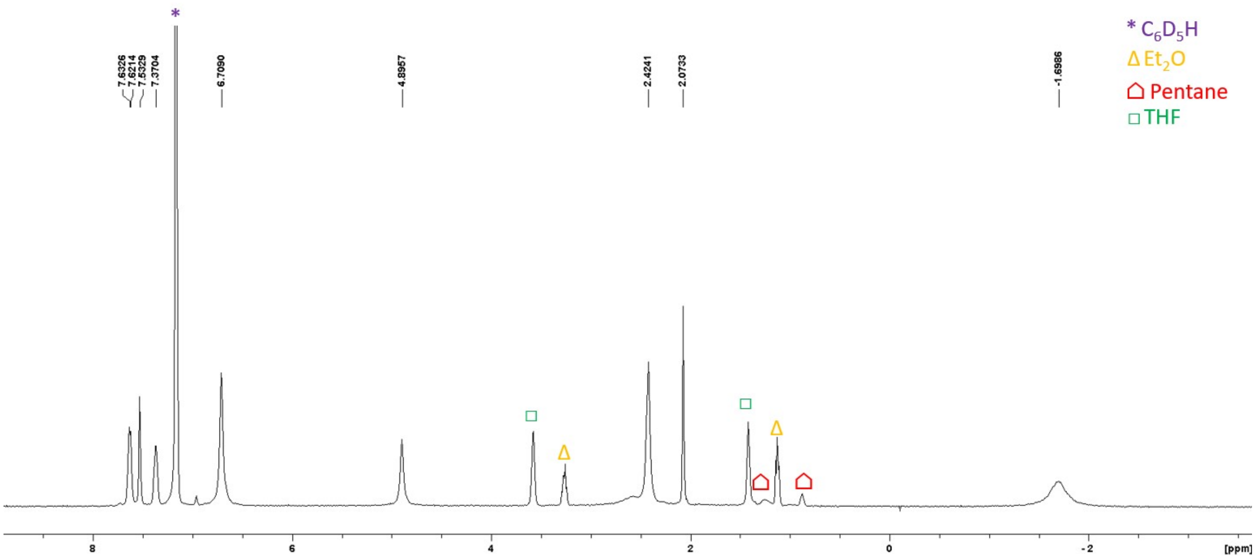

Figure S4. Solid state (ATR) IR spectrum of $Ph_3COZr(MesNP^iPr_2)_3CoCN^tBu$ (**4**).

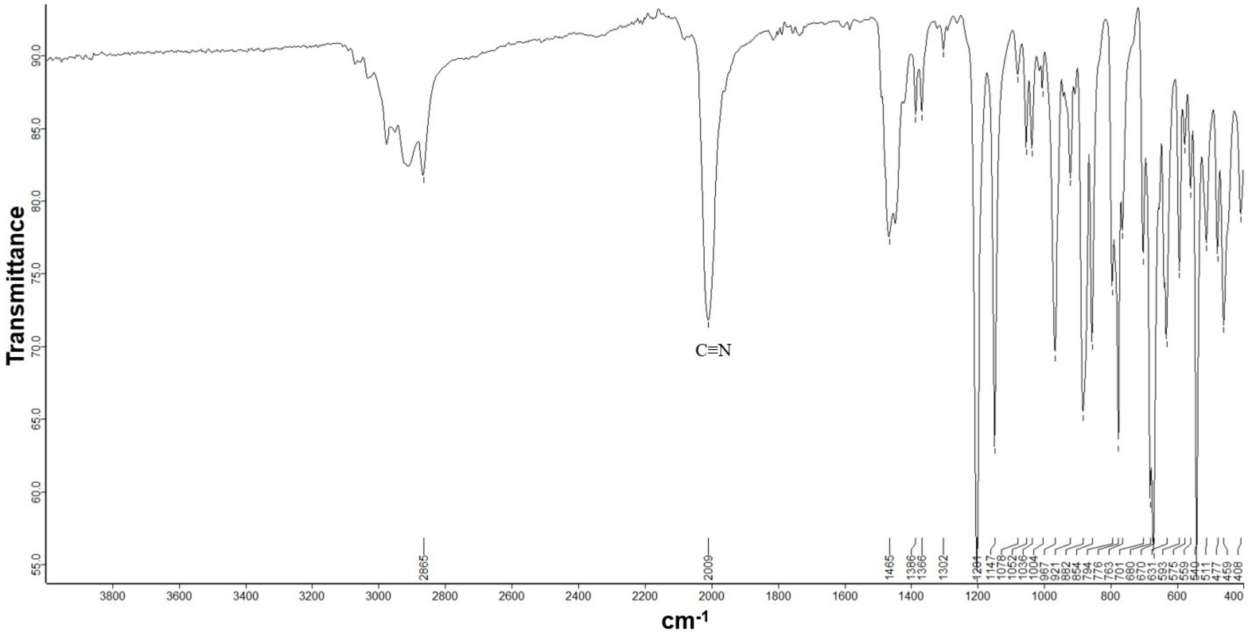

Figure S5. Positive ion ESI-MS spectrum of **4** in PhF. One predominant Zr/Co species was observed in full spectrum: $[Ph_3COZr(MesNP^iPr_2)_3CoCN^tBu]^+$ (*m/z* = 1241.5404). Isotopic peaks are consistent between predicted (bottom left) and experimental (bottom right) spectra for Zr/Co species.

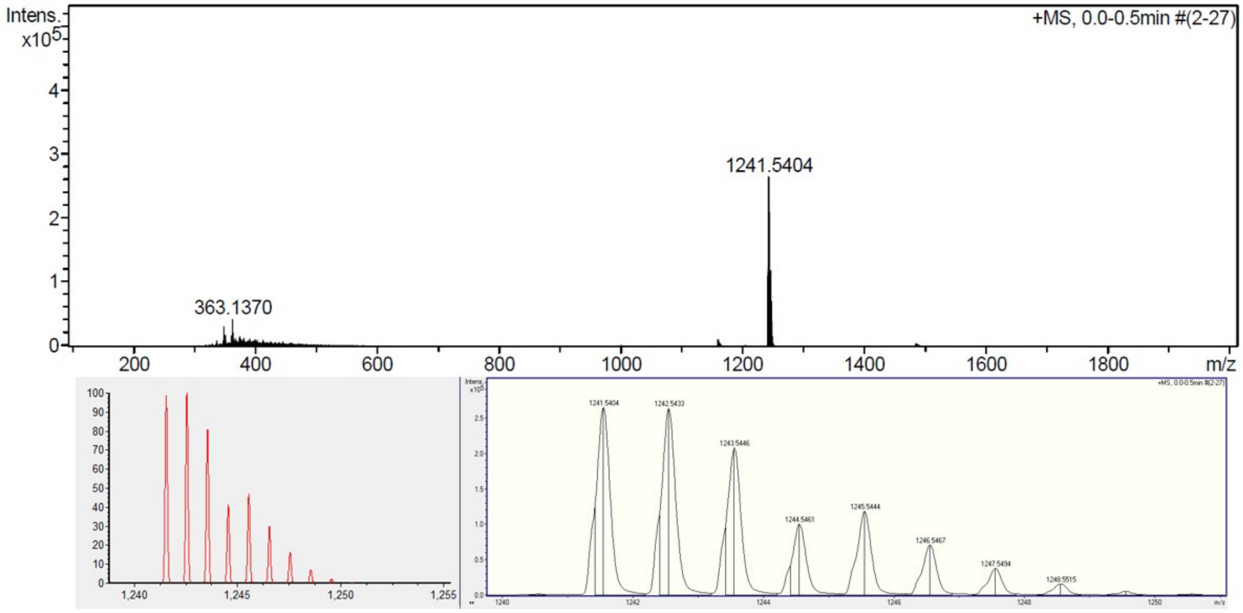

Figure S6. Experimental (black) and simulated (red) X-band EPR spectra of compound **4** showing spin localization on ^{59}Co (I = 7/2). Spectrum was obtained in frozen fluorobenzene at 30 K with two scans (power attenuation = 30 dB, modulation amplitude = 10 G, modulation frequency = 100 kHz). Simulation parameters: g = 2.00, 2.11, and 2.33; A = 130, 109, and 82 MHz.

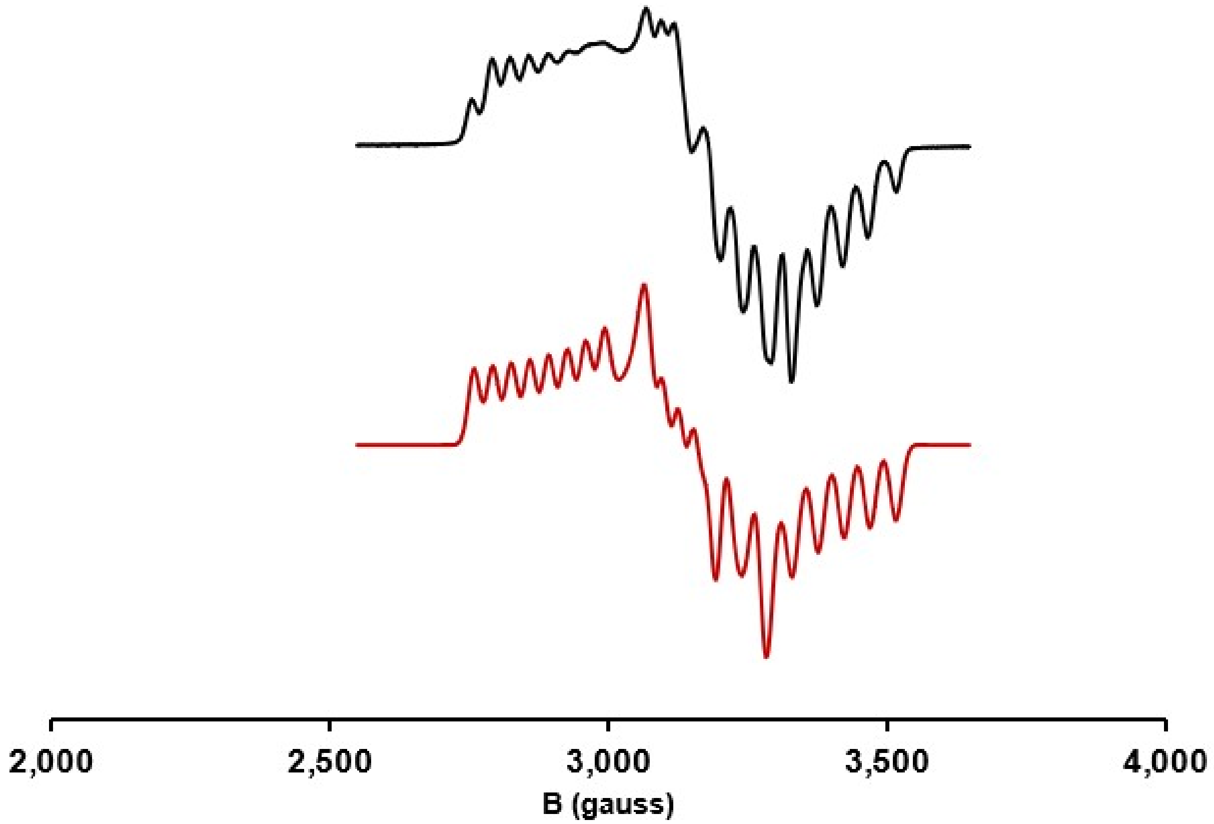

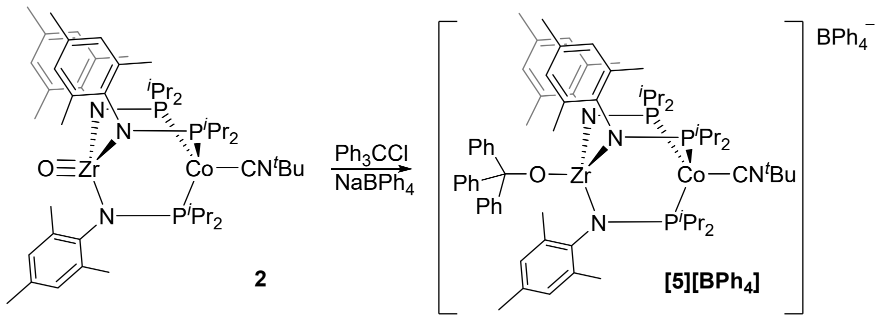

Synthesis of [Ph$_3$COZr(MesNP^{i}Pr$_2$)$_3$CoCN^{t}Bu][BPh$_4$] (**[5][BPh$_4$]**).

Oxo compound **2** (66.0 mg, 0.066 mmol) was suspended in fluorobenzene (1 mL) and stirred for ~ 2 min to form a bright green suspension. Trityl chloride (20.9 mg, 0.075 mmol) was dissolved in fluorobenzene (1 mL) and added into the stirring suspension of **2,** and the resulting mixture was stirred for ~ 1 min. The resulting cloudy bright green mixture was then added into a stirring suspension of NaBPh$_4$ (24.2 mg, 0.071 mmol) in fluorobenzene (1 mL). The reaction became a bright green clear solution and a white precipitate formed (NaCl). This reaction mixture was for stirred 16 h at room temperature to ensure reaction completion. The solution was filtered through a plug of glass microfiber filter paper to remove the white solid, and the green-yellow filtrate was dried in vacuo to afford a pale green solid. The solid residue was first triturated with fluorobenzene (2 mL), and then washed with fluorobenzene (1 mL) to afford the product as a bright green solid. Yield: 80.4 mg, 80.0%. A single crystal suitable for X-ray diffraction was obtained by slow diffusion of diethyl ether into a concentrated solution of **[5][BPh$_4$]** in CH$_2$Cl$_2$ at room temperature. ^{1}H NMR (400 MHz, CD$_2$Cl$_2$): δ 24.38 (br), 10.00 (br), 7.80, 7.54, 7.43, 7.25, 6.97, 6.81, 2.22 (br), 2.15, -3.95 (br), -10.66 (br). ^{11}B NMR (192 MHz, CD$_2$Cl$_2$): δ -6.70. Evans' method (μ_{eff}, CD$_2$Cl$_2$): 2.95 B.M. UV-vis (CH$_2$Cl$_2$, λ(nm) (ε, M^{-1}cm^{-1})): 894 (420), 655 (10), 353 (925, sh). ATR IR: 2148 cm^{-1} ($\nu_{C\equiv N}$). ESI-HRMS (C$_6$H$_5$F, positive mode): Calcd *m/z* for

[$Ph_3COZr(MesNP^iPr_2)_3CoCN^tBu]^+$: 1241.5411. Found: 1241.5271. ESI-HRMS (C_6H_5F, negative mode): Calcd *m/z* for $[BPh_4]^-$: 319.1658. Found: 319.1667.

Figure S7. ^1H NMR spectrum (400 MHz, C_6D_6) of [$Ph_3COZr(MesNP^iPr_2)_3CoCN^tBu$][$BPh_4$] (**[5][BPh₄]**). Peaks corresponding to residual solvents, in this case $CDHCl_2$ and Et_2O, are labelled.

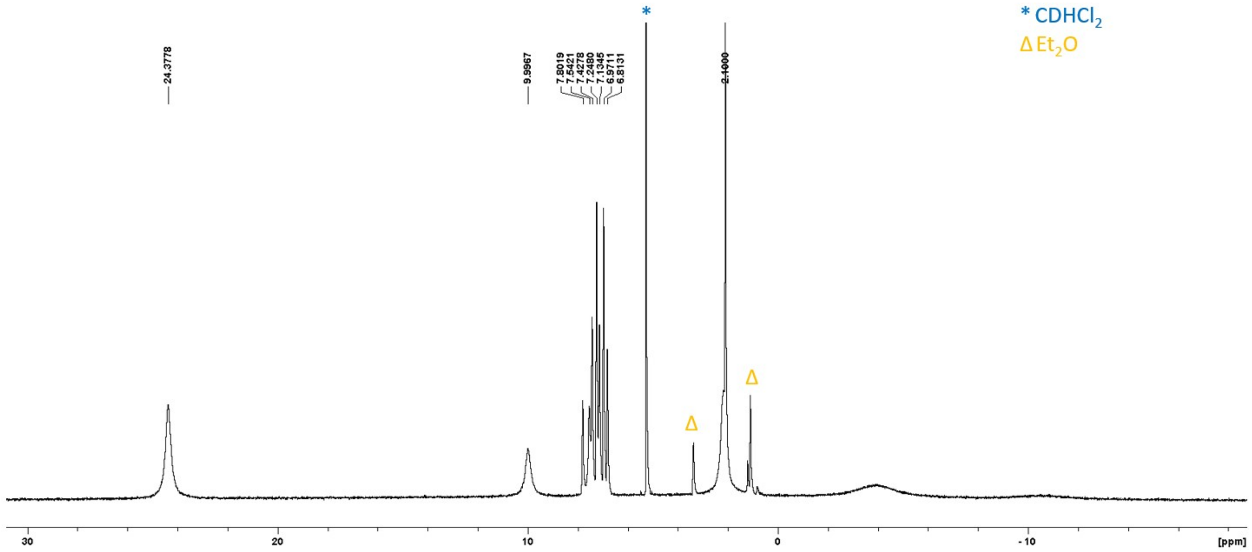

Figure S8. ^{11}B NMR spectrum (192 MHz, C_6D_6) of [$Ph_3COZr(MesNP^iPr_2)_3CoCN^tBu$][$BPh_4$] (**[5][BPh₄]**).

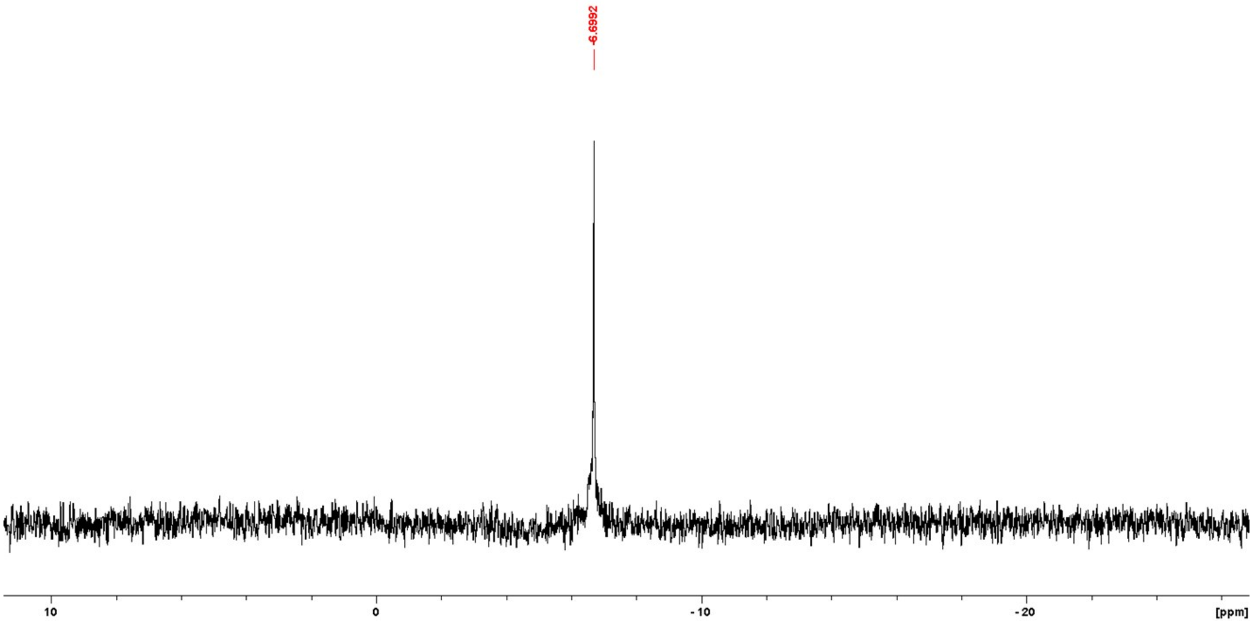

Figure S9. Solid state (ATR) IR spectrum of [Ph_3COZr(MesNP^{i}Pr$_2$)$_3$CoCN^{t}Bu][BPh_4] (**[5][BPh$_4$]**).

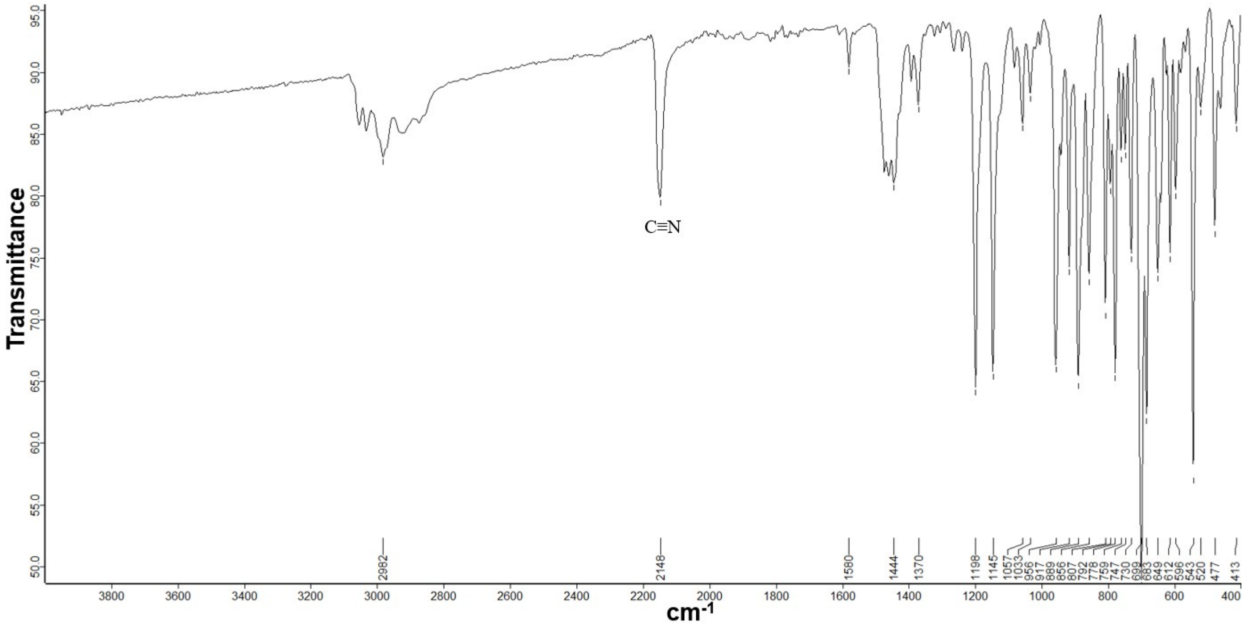

Figure S10. Positive ion ESI-MS spectrum of **[5][BPh$_4$]** in PhF. Two predominant Zr/Co species were observed in full spectrum: [Ph_3COZr(MesNP^{i}Pr$_2$)$_3$CoCN^{t}Bu]$^+$ (m/z = 1241.5271) and [Ph_3COZr(MesNP^{i}Pr$_2$)$_3$Co]$^+$ (m/z = 1158.4552). Isotopic peaks are consistent between predicted (bottom left) and experimental (bottom right) spectra for Zr/Co species.

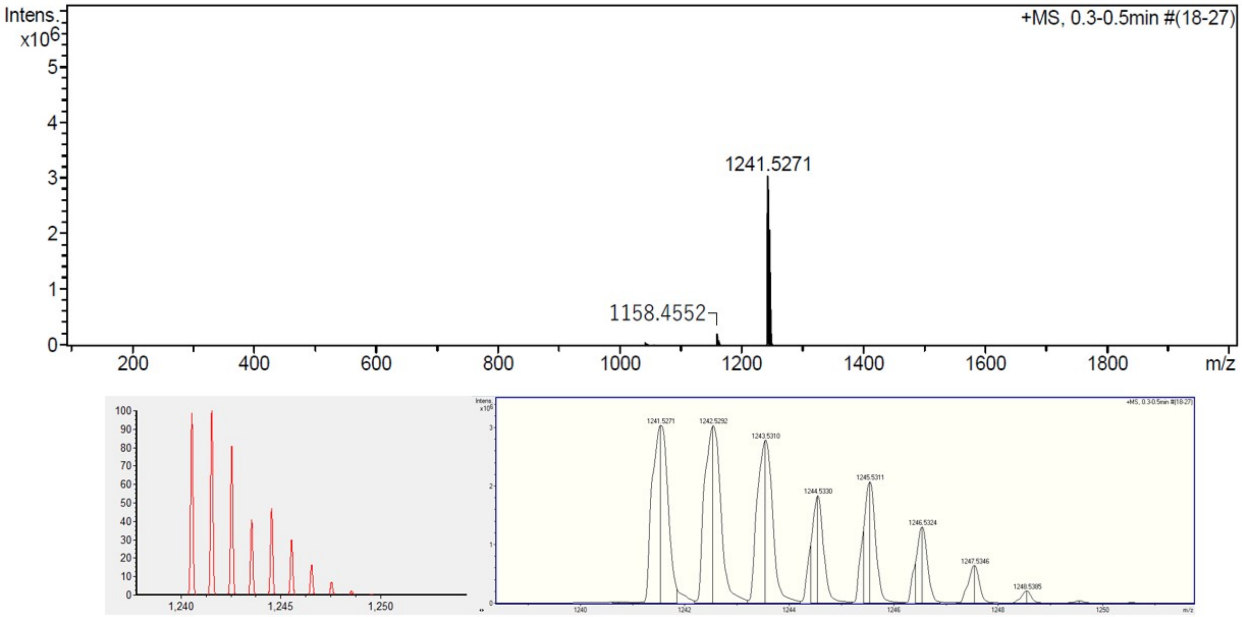

Figure S11. Negative ion ESI-MS spectrum of **[5][BPh$_4$]** in PhF. One predominant species was observed in full spectrum: $[BPh_4]^-$ (*m/z* = 319.1667). Isotopic peaks are consistent between predicted (bottom left) and experimental (bottom right) spectra.

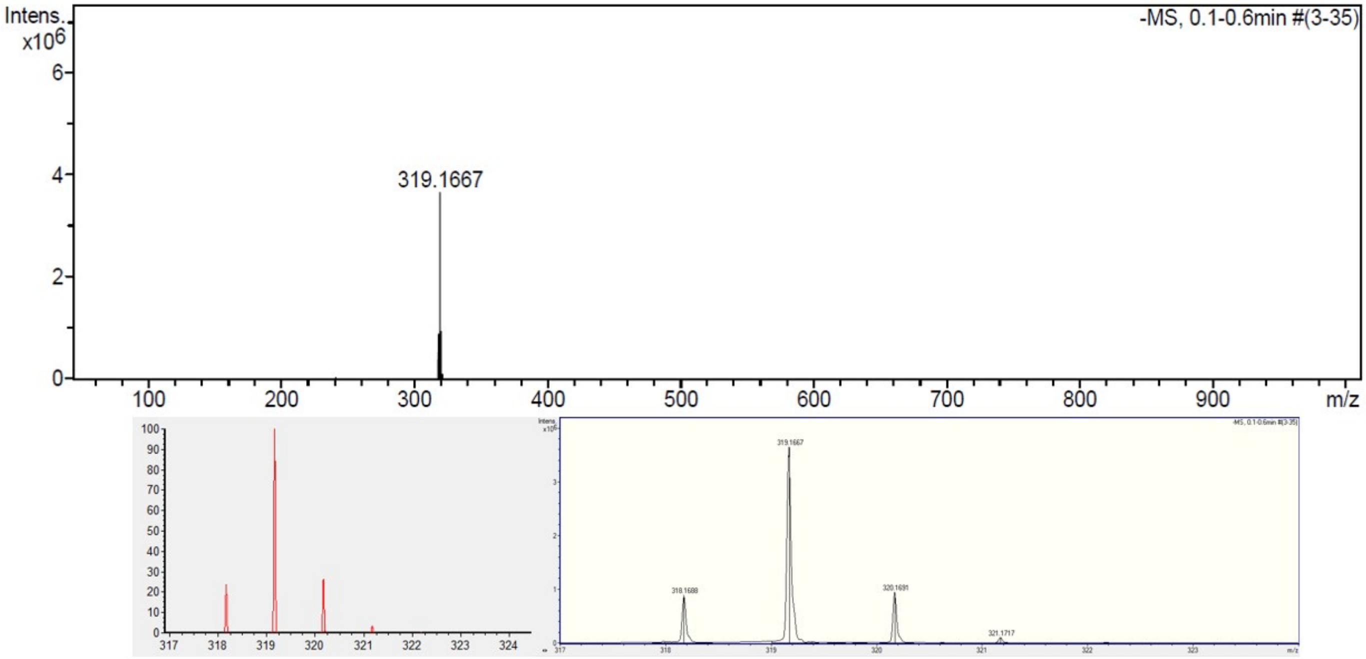

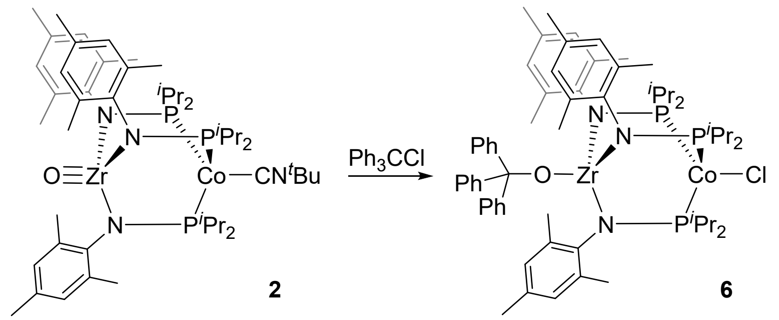

2 **6**

Synthesis of $Ph_3COZr(MesNP^iPr_2)_3CoCl$ (**6**)

Oxo compound **2** (79.4 mg, 0.0794 mmol) was suspended in fluorobenzene (1.5 mL) and stirred for ~ 2 min to form a cloudy bright green suspension. Trityl chloride (23.3 mg, 0.0837 mmol) was dissolved in fluorobenzene (1.5 mL) and added into the stirring suspension of **2**. The reaction became homogeneous and light green after ~25 min, and was allowed to stir for 20 h. The volatile components were removed under vacuum to afford a green-blue residue, which was washed with diethyl ether (3 x 3 mL) to precipitate a light blue solid product. The product was purified through recrystallization by extracting the solids into fluorobenzene and allowing pentane vapor to slowly diffuse into the solution at room temperature. Yield: 53.1 mg, 55.9%. Single crystals suitable for X-ray diffraction were grown via diffusion of pentane into a concentrated fluorobenzene solution of **6** at -30 °C. ^1H NMR (400 MHz, C_6D_6): δ 15.94 (br), 7.56, 6.94, 6.66 (br), 6.09, 1.96, 1.81 (br), -1.69 (br). Evans' method (μ_{eff}, CD_2Cl_2): 2.81 B.M. UV-vis (C_6H_5F, λ(nm) (ε, $M^{-1}cm^{-1}$)): 876 (170), 627 (40), 564 (30). ESI-HRMS (C_6H_5F, positive mode): Calcd *m/z* for $[Ph_3COZr(MesNP^iPr_2)_3CoCl]^+$: 1193.4364. Found: 1193.4315.

Figure S12. ^{1}H NMR spectrum (400 MHz, C_6D_6) of $Ph_3COZr(MesNP^{i}Pr_2)_3CoCl$ (**6**). Peaks corresponding to residual solvents, in this case $CDHCl_2$, and Et_2O, are labelled.

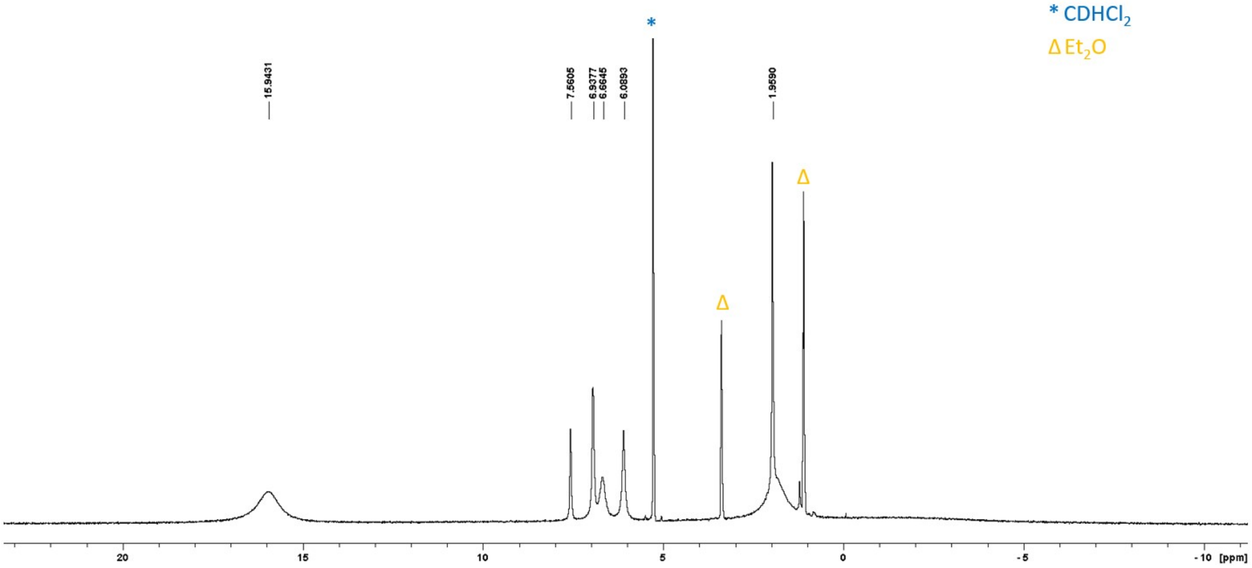

Figure S13. Solid state (ATR) IR spectrum of $Ph_3COZr(MesNP^{i}Pr_2)_3CoCl$ (**6**).

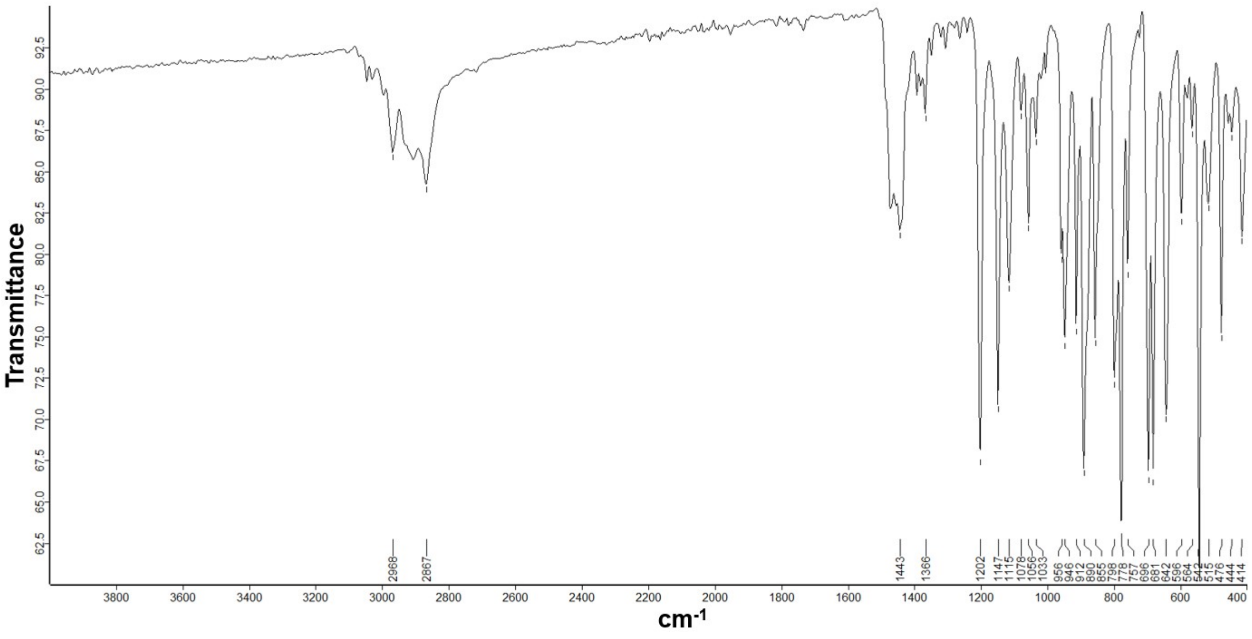

Figure S14. Positive ion ESI-MS spectrum of **6** in PhF. One predominant Zr/Co species was observed in the full spectrum: $[Ph_3COZr(MesNP^iPr_2)_3CoCl]^+$ (*m/z* = 1193.4315). Isotopic peaks are consistent between predicted (bottom left) and experimental (bottom right) spectra for Zr/Co species.

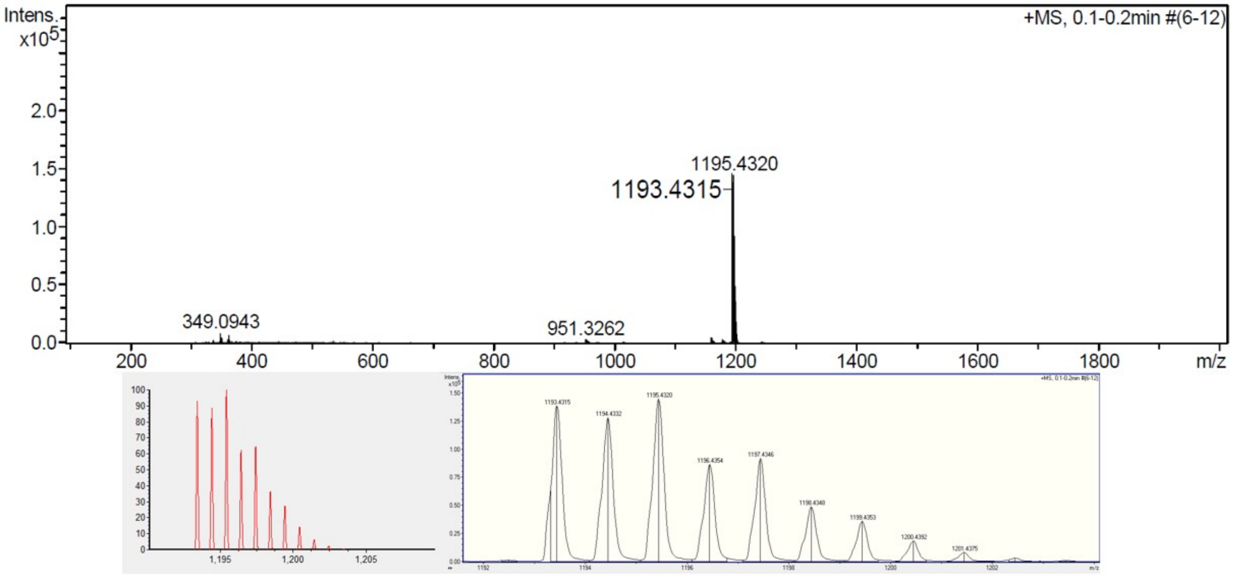

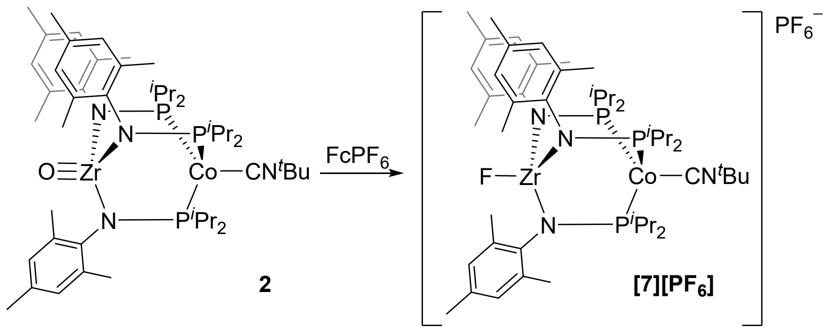

Synthesis of [FZr(MesNP^{i}Pr$_2$)$_3$CoCN^{t}Bu][PF$_6$] (**[7][PF$_6$]**).

Compound **2** (110.4 mg, 0.11 mmol) was suspended in fluorobenzene (~1 mL) and stirred for 1 min. FcPF$_6$ (36.5 mg, 0.11 mmol) was suspended in fluorobenzene (~1 mL) and stirred for 1 min. The suspension of FcPF$_6$ suspension was added into the stirring suspension of **2**, and the mixture quickly changed color from a light green to light brown, and then to dark green within one minute. The mixture became homogeneous after stirring for a few minutes, and was allowed to stir for 20 min. The volatile components were removed in vacuo and the remaining dark residue was washed with pentane (4 mL) to remove ferrocene and afford a powder. The residue was then extracted into THF (1 mL) and pentane vapor was allowed to slowly diffuse into the solution to crystallize the product. After decanting the supernatant, the light yellow solid product was washed with THF (1 mL) to obtain pure product. Yield: 30.4 mg, 24.1%. Crystals suitable for X-ray diffraction were grown via diffusion of pentane vapor into a concentrated fluorobenzene solution of **[7][PF$_6$]** at room temperature. ^{1}H NMR (400 MHz, CD_2Cl_2): δ 23.31 (br), 9.64 (br), 7.57, 2.76 (br), 2.25, -4.11. ^{31}P NMR (162 MHz, CD_2Cl_2): δ -145.65 (m, $J_{F\text{-}P}$ = 709.8 Hz). ^{19}F NMR (376 MHz, CD_2Cl_2): δ -74.47 (d, $J_{F\text{-}P}$ = 711.2 Hz). Evans' method (μ_{eff}, CD_2Cl_2): 2.95 B.M. UV-vis (C_6H_5F, λ(nm) (ε, M^{-1}cm^{-1})): 880 (260), 619 (30). ATR IR: 2164 cm^{-1} ($\nu_{C\equiv N}$). ESI-HRMS (C_6H_5F, positive mode): Calcd *m/z* for [FZr(MesNP^{i}Pr$_2$)$_3$CoCN^{t}Bu]$^+$: 1001.4272. Found: 1001.4374.

Figure S15. ^{1}H NMR spectrum (400 MHz, CD_2Cl_2) of [FZr(MesNP^{i}Pr$_2$)$_3$CoCN^{t}Bu][PF_6] (**[7][PF$_6$]**). Peaks corresponding to residual solvents, in this case $CDHCl_2$, Et_2O, THF, and PhF, are labelled.

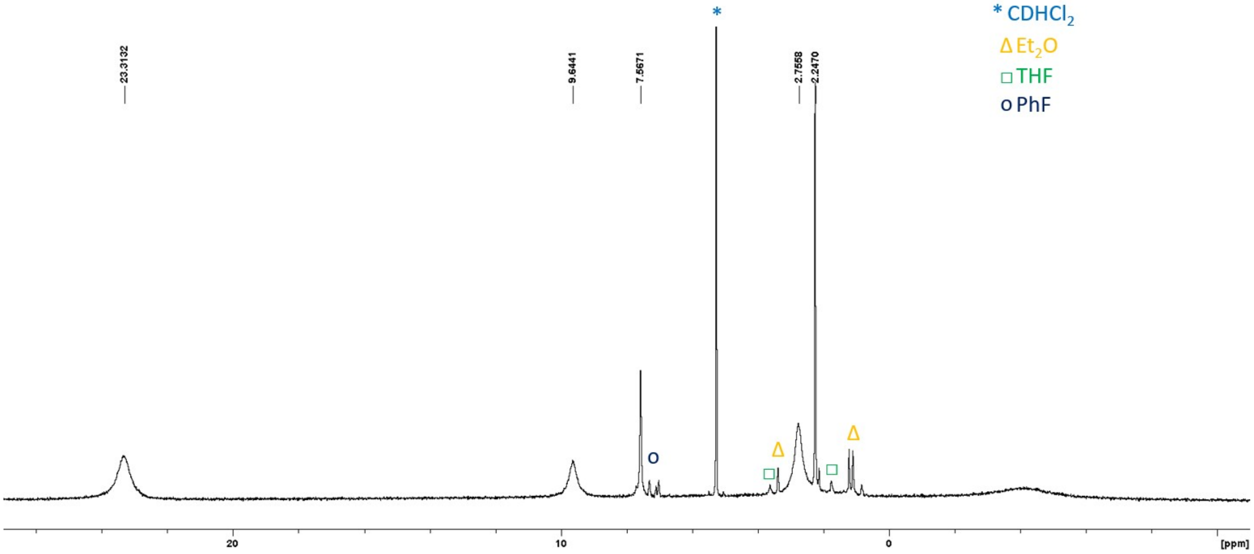

Figure S16. ^{31}P{^{1}H} NMR spectrum (162 MHz, CD_2Cl_2) of [FZr(MesNP^{i}Pr$_2$)$_3$CoCN^{t}Bu][PF_6] (**[7][PF$_6$]**).

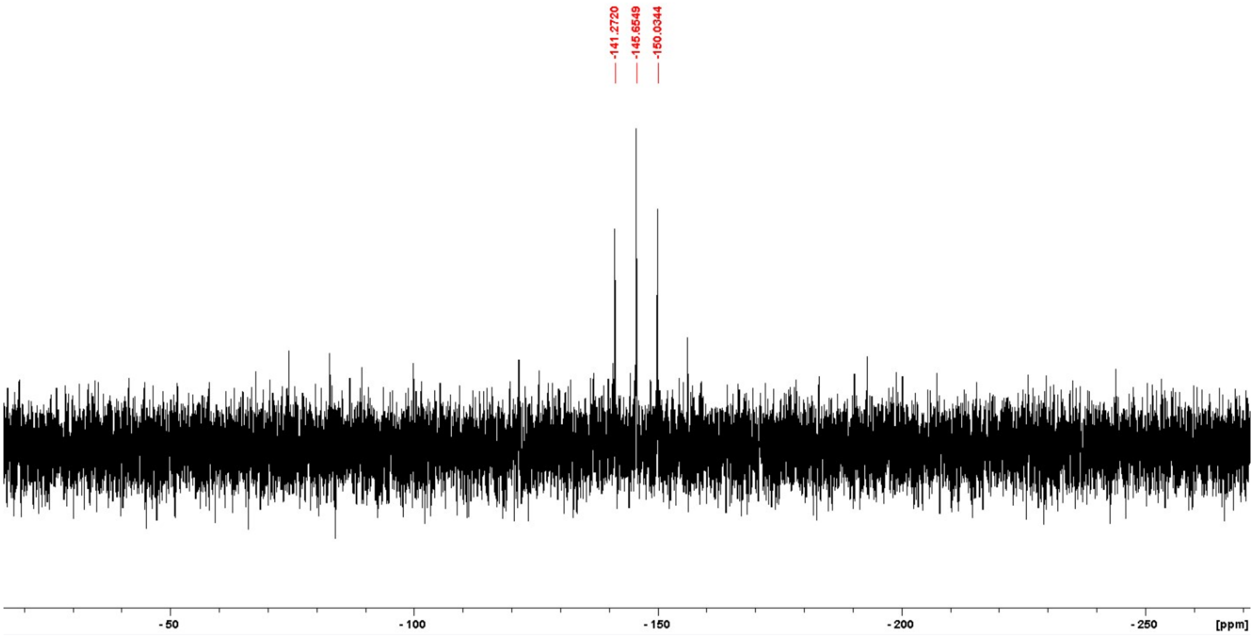

Figure S17. ^{19}F NMR spectrum (376 MHz, CD_2Cl_2) of [FZr(MesNP^{i}Pr$_2$)$_3$CoCN^{t}Bu][PF$_6$] (**[7][PF$_6$]**).

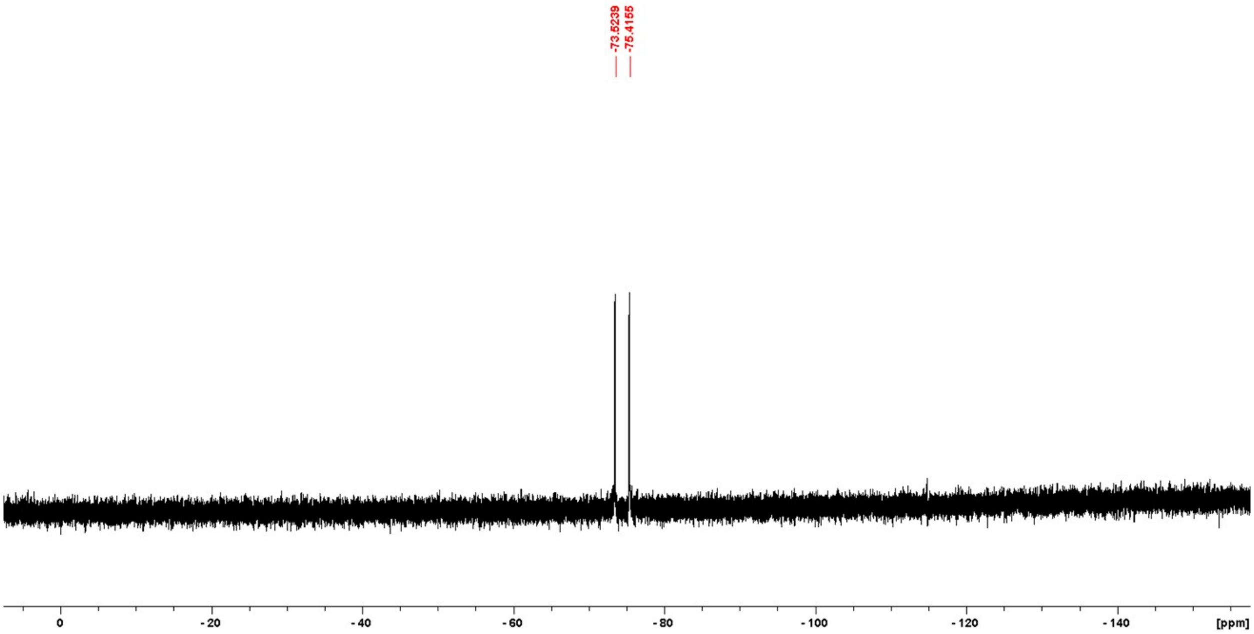

Figure S18. Solid state (ATR) IR spectrum of [FZr(MesNP^{i}Pr$_2$)$_3$CoCN^{t}Bu][PF$_6$] (**[7][PF$_6$]**).

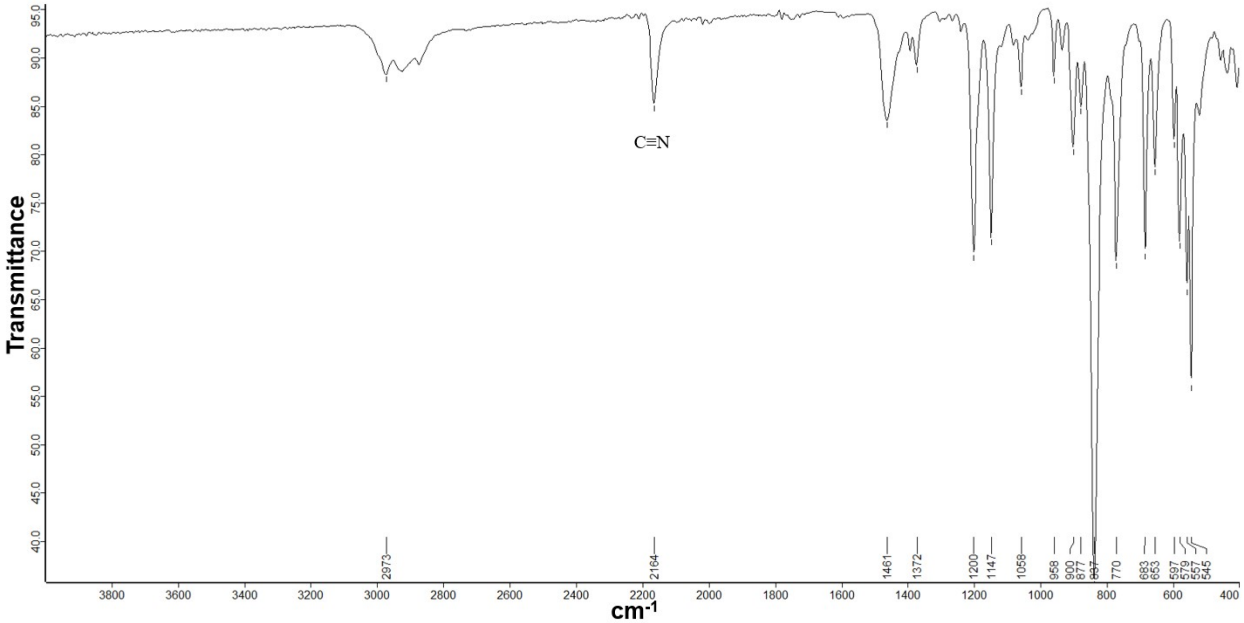

Figure S19. Positive ion ESI-MS spectrum of **[7][PF$_6$]** in PhF. Two predominant species containing Zr/Co were observed in the full spectrum: [FZr(MesNP^{i}Pr$_2$)$_3$CoCN^{t}Bu]$^+$ (*m/z* = 1001.4374) and [FZr(MesNP^{i}Pr$_2$)$_3$Co]$^+$ (*m/z* = 918.3540). Isotopic peaks are consistent between predicted (bottom left) and experimental (bottom right) spectra for Zr/Co species.

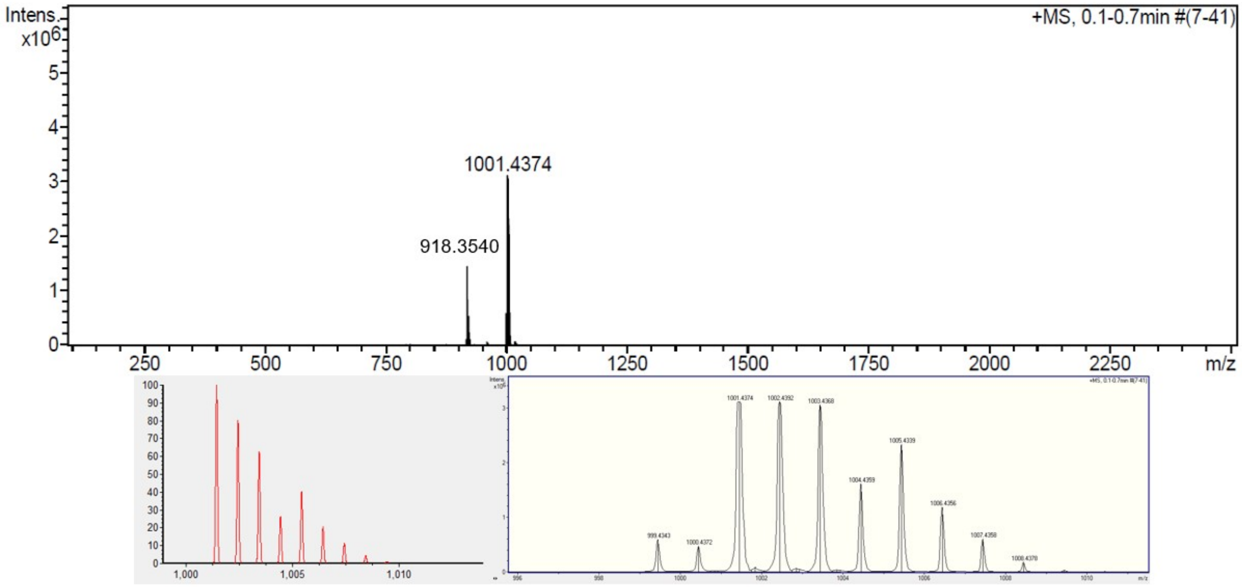

Figure S20. Full cyclic voltammogram (CV) of **[7][PF$_6$]** vs. Fc/Fc$^+$. CV was collected in 0.3 M [^{n}Bu$_4$N][PF$_6$] THF solution, scanning cathodically starting from the open circuit potential with a scan rate of 100 mV/s.

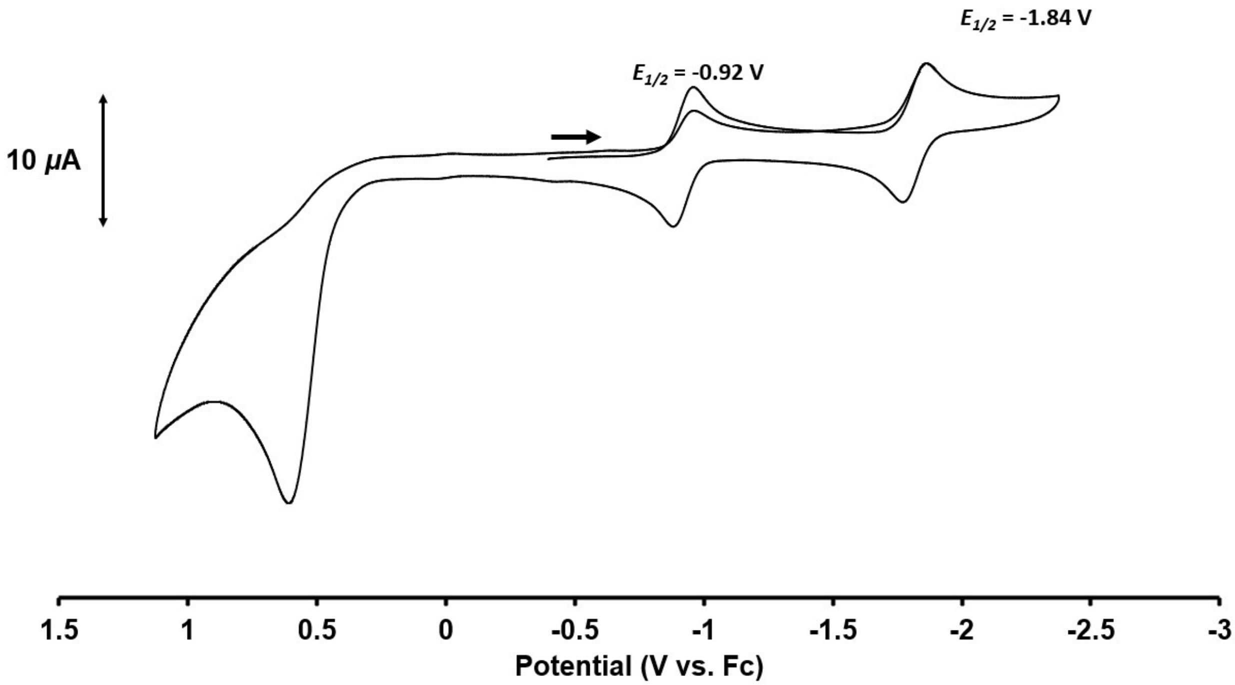

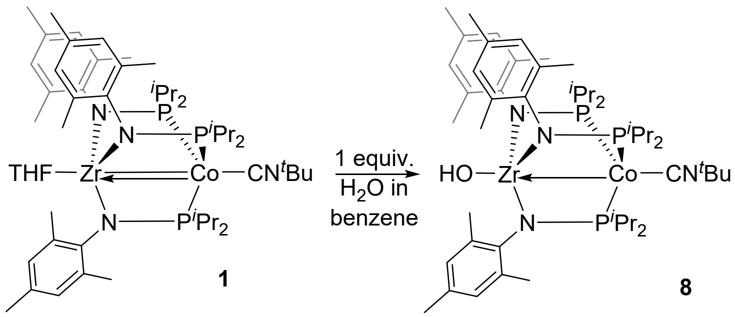

Synthesis of HOZr(MesNP^{i}Pr$_2$)$_3$CoCN^{t}Bu (**8**)

(THF)Zr(MesNP^{i}Pr$_2$)$_3$CoCN^{t}Bu (**1**) was generated *in situ* from THFZr(MesNP^{i}Pr$_2$)$_3$CoN$_2$ (92.4 mg, 0.0924 mmol) following the previously reported procedure[1] and was used without further purification. To the dark brown solution of **1** (assuming 100% conversion, 97.5 mg, 0.0924 mmol) was added a saturated solution of water in benzene (10.51 g, 686.7 ppm H_2O at 23 °C/1 atm).[10] The color of the solution quickly changed from dark brown to light yellow. The reaction was stirred for 5 min. The solvent was evaporated in vacuo, and the brown-yellow residue was extracted into pentane. The resulting cloudy light yellow suspension was filtered through a plug of glass microfiber filter paper and the filtrate was then concentrated in vacuo and stored at -30 °C to afford yellow crystalline product. Crystals suitable for X-ray diffraction were grown in the same fashion using slightly more pentane solvent to slow the crystal growth rate. Yield: 51.5 mg, 55.7% with respect to THFZr(MesNP^{i}Pr$_2$)$_3$CoN$_2$. ^{1}H NMR (400 MHz, C_6D_6): δ 7.59, 6.15, 5.41 (br), 4.83, 2.89, 2.17, -1.89 (br), -45.14 (br). Evans' method (μ_{eff}, C_6D_6): 1.64 B.M. UV-vis (C_6H_5F, λ(nm) (ε, M^{-1}cm^{-1})): 408 (2.27×10^3, sh). ATR IR: 2009 cm^{-1} ($\nu_{C\equiv N}$), 3701 cm^{-1} ($\nu_{O\text{-}H}$). Owing to the reactivity of **8** with air, moisture, and protic solvents, satisfactory elemental analysis and/or high-resolution ESI-MS data could not be obtained.

Figure S21. ^{1}H NMR spectrum (400 MHz, C_6D_6) of HOZr(MesNP^{i}Pr$_2$)$_3$CoCN^{t}Bu (**8**). Peaks corresponding to residual solvents, in this case C_6D_5H, THF, and pentane, are labelled.

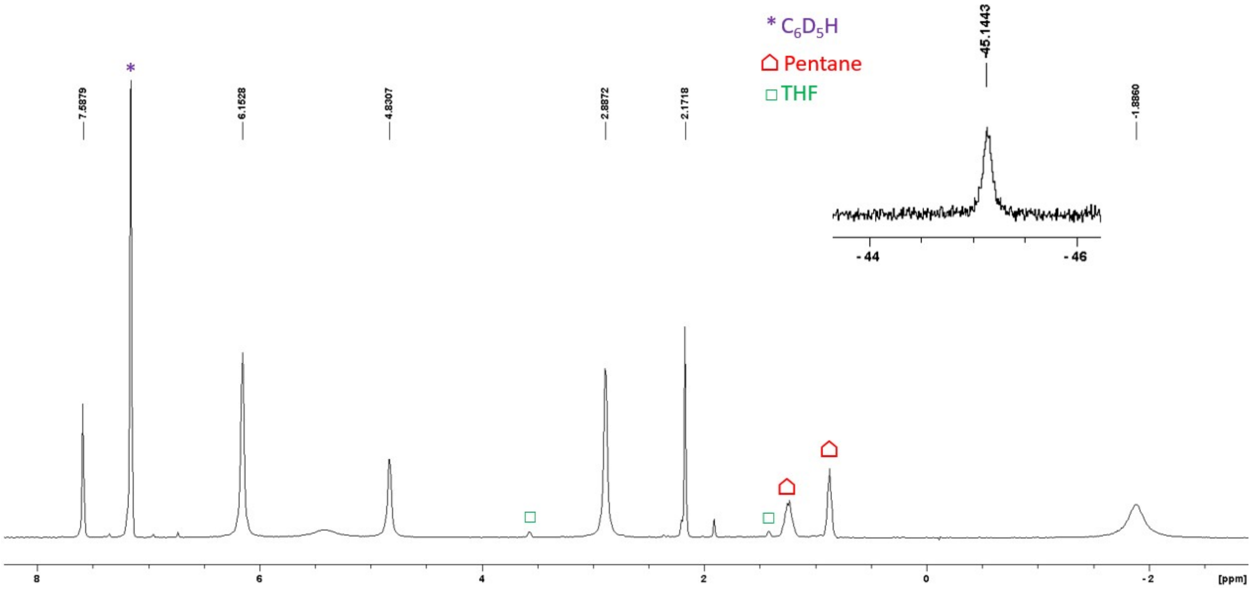

Figure S22. Solid state (ATR) IR spectrum of HOZr(MesNP^{i}Pr$_2$)$_3$CoCN^{t}Bu (**8**).

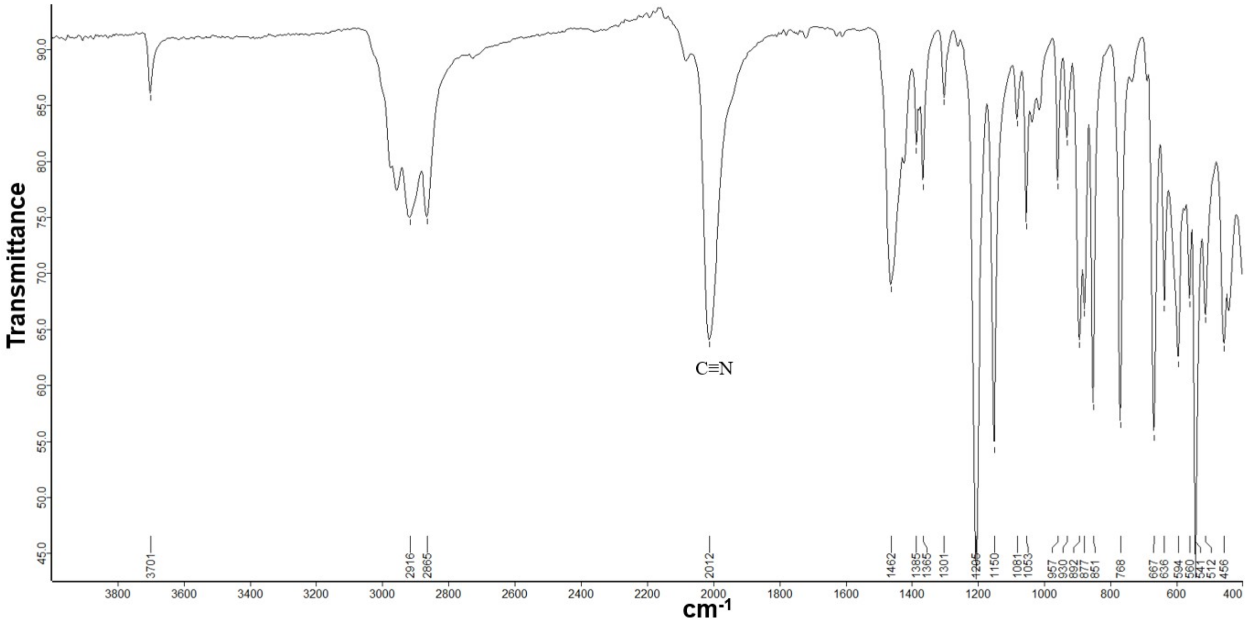

Figure S23. Experimental (black) and simulated (red) X-band EPR spectra of compound **8** showing spin localization on ^{59}Co (I = 7/2). Spectrum was obtained in frozen fluorobenzene at 30 K with two scans (power attenuation = 30 dB, modulation amplitude = 10 G, modulation frequency = 100 kHz.). Simulation parameters: g = 2.00, 2.10, and 2.33; A = 132, 110, and 100 MHz.

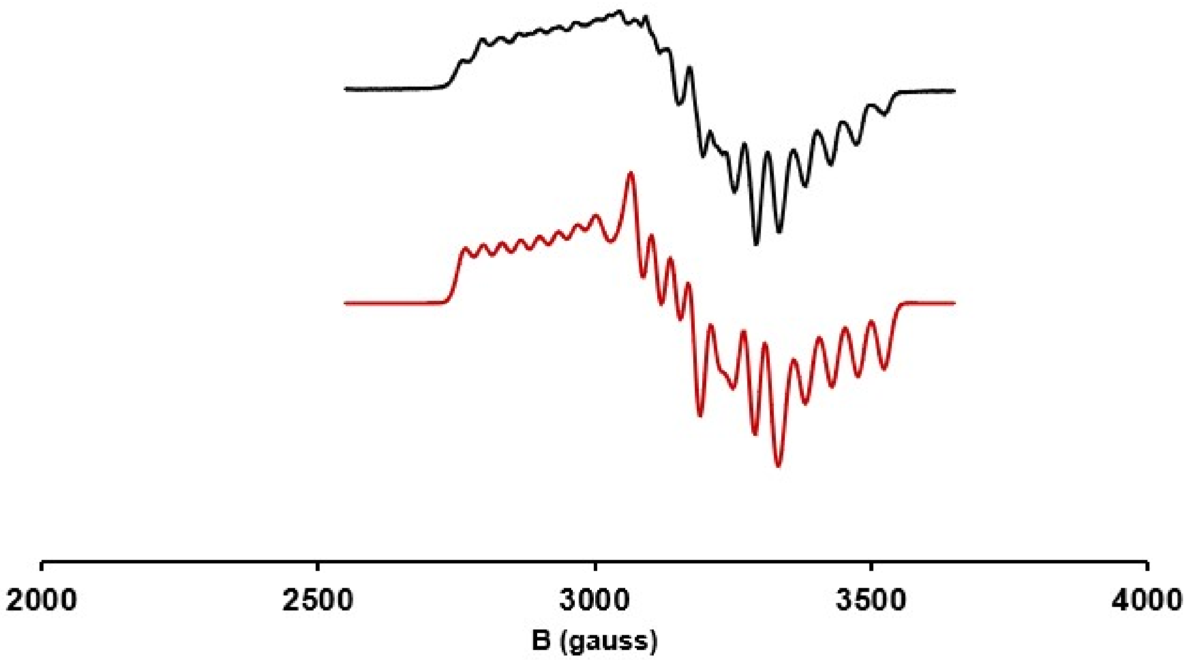

Figure S24. Full cyclic voltammogram (CV) of **8** vs. Fc/Fc$^+$. CV was collected in 0.3 M [^{n}Bu$_4$N][PF$_6$] THF solution, scanning cathodically starting from open circuit potential with a scan rate of 100 mV/s.

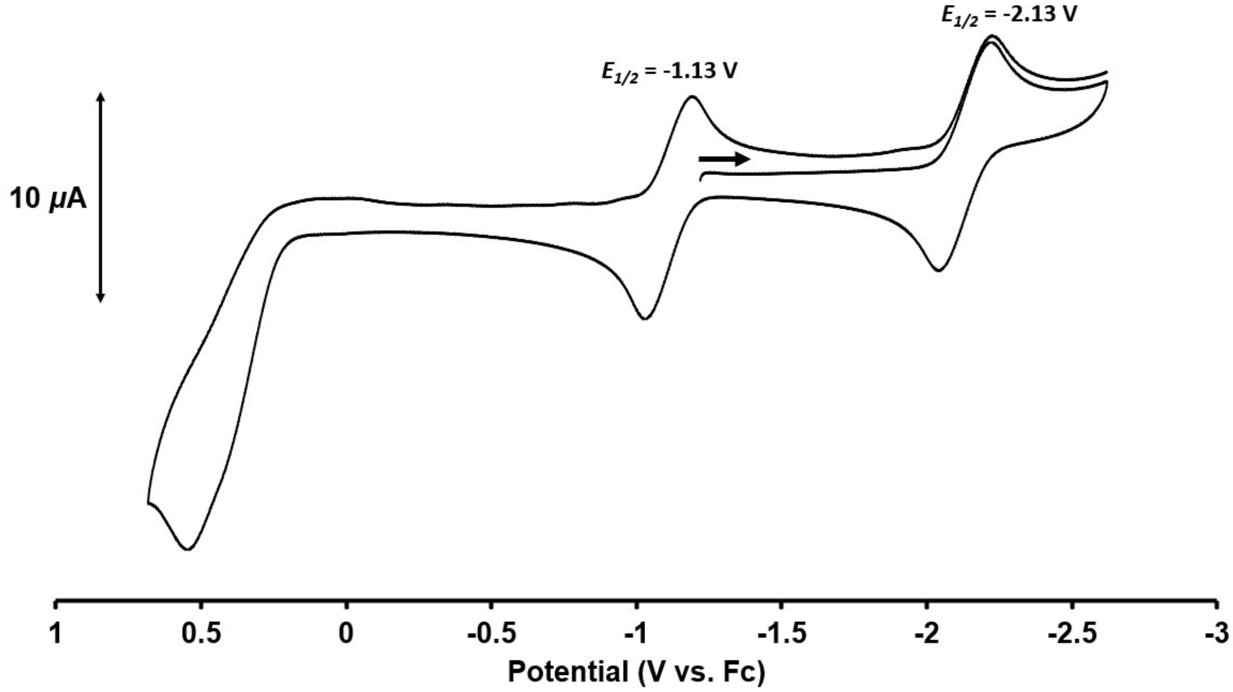

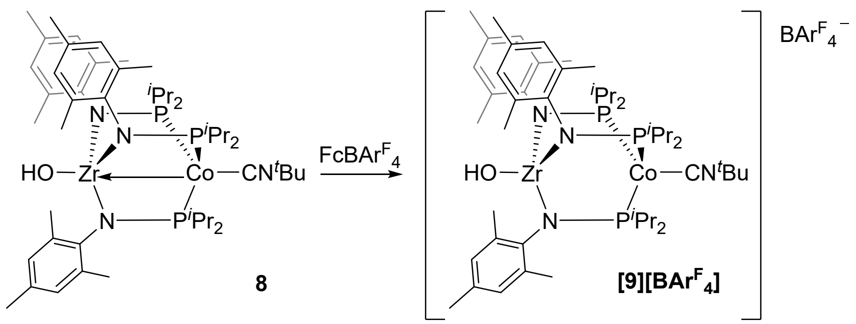

Synthesis of [HOZr(MesNP^{i}Pr$_2$)$_3$CoCN^{t}Bu][BAr$^F_4$] (**[9][BAr$^F_4$]**)

Compound **8** (24.0 mg, 0.024 mmol) was dissolved in fluorobenzene (2 mL) to form a yellow solution. FcBAr$^F_4$ (26.1 mg, 0.025 mmol) was suspended in fluorobenzene and was stirred briefly for ~ 1 min. The blue fluorobenzene suspension of FcBAr$^F_4$ was added into the stirring yellow solution of **8**. The supernatant color remained yellow and became darker as the reaction proceeded, while the undissolved FcBAr$^F_4$ became fully dissolved after stirring for 10 min. The reaction was allowed to stir 20 min to ensure reaction completion. The volatile components were removed from the dark brown-yellow solution in vacuo. The resulting residue was triturated with pentane (2 x 2 mL), and then washed with pentane (2 x 2 mL) to remove the ferrocene side product and afford a light yellow solid product. Yield: 34.1 mg, 76.3%. Single crystals suitable for X-ray diffraction were obtained by diffusion of pentane vapor into a saturated fluorobenzene solution of **[9][BAr$^F_4$]** at room temperature. ^{1}H NMR (400 MHz, CD_2Cl_2): δ 22.97 (br), 9.73 (br), 7.66 ([BAr$^F_4$]), 7.49 ([BAr$^F_4$]), 2.81 (br), 2.25, -4.53, -59.93 (br). ^{11}B NMR (192 MHz, CD_2Cl_2): δ -6.52. ^{19}F NMR (565 MHz, CD_2Cl_2): δ -61.8. Evans' method (μ_{eff}, C_6D_6): 2.59 B.M. UV-vis (C_6H_5F, λ(nm) (ε, $M^{-1}cm^{-1}$)): 895 (360), 669 (30), 626 (30), 424 (285, sh). ATR IR: 2152 cm^{-1} ($\nu_{C\equiv N}$), 3703 cm^{-1} ($\nu_{O\text{-}H}$). ESI-HRMS (C_6H_5F, positive mode): Calcd *m/z* for [HOZr(MesNP^{i}Pr$_2$)$_3$CoCN^{t}Bu]$^+$: 999.4315. Found: 999.4539. ESI-HRMS (C_6H_5F, negative mode): Calcd *m/z* for [BAr$^F_4$]$^-$: 863.0649. Found: 863.0556.

Figure S25. ^{1}H NMR spectrum (400 MHz, CD_2Cl_2) of [HOZr(MesNP^{i}Pr$_2$)$_3$CoCN^{t}Bu][BAr$^F{}_4$] (**[9][BAr$^F{}_4$]**). Peaks corresponding to residual solvents, in this case $CDHCl_2$ and pentane, are labelled.

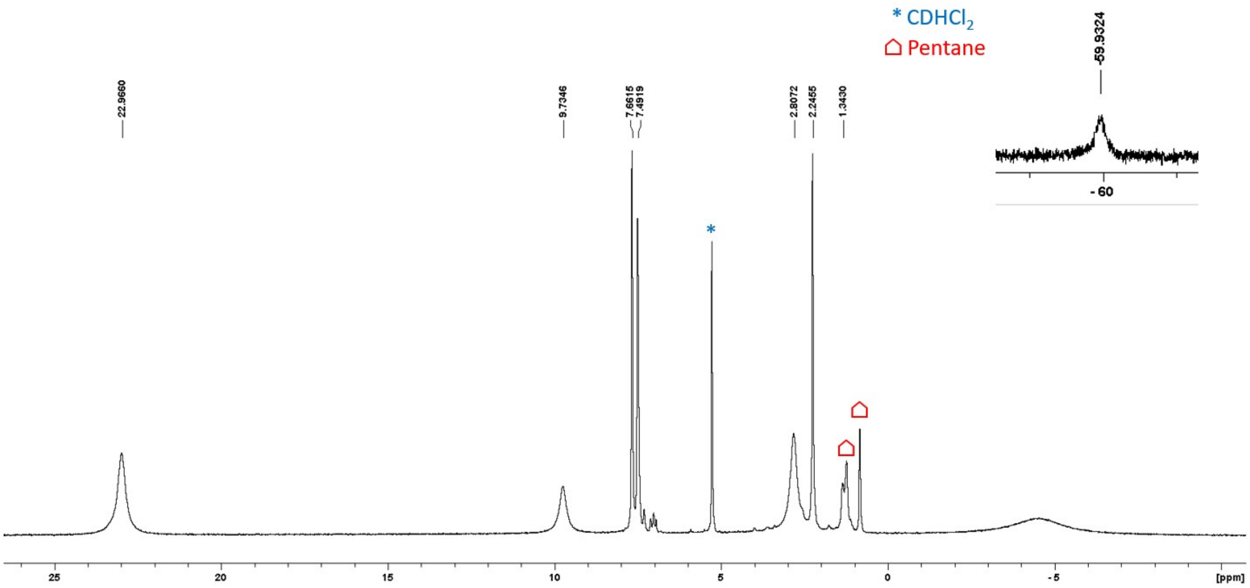

Figure S26. ^{11}B NMR spectrum (192 MHz, CD_2Cl_2) of [HOZr(MesNP^{i}Pr$_2$)$_3$CoCN^{t}Bu][BAr$^F{}_4$] (**[9][BAr$^F{}_4$]**).

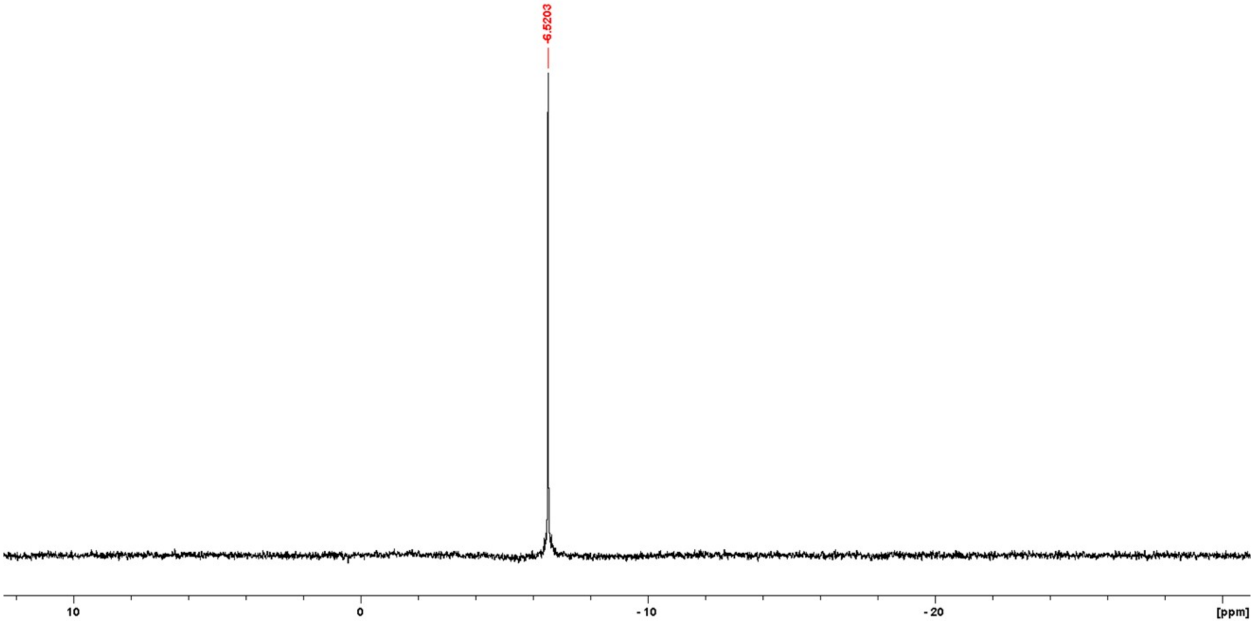

Figure S27. ^{19}F NMR spectrum (565 MHz, CD_2Cl_2) of [HOZr(MesNP^{i}Pr$_2$)$_3$CoCN^{t}Bu][BAr$^F{}_4$] (**[9][BAr$^F{}_4$]**).

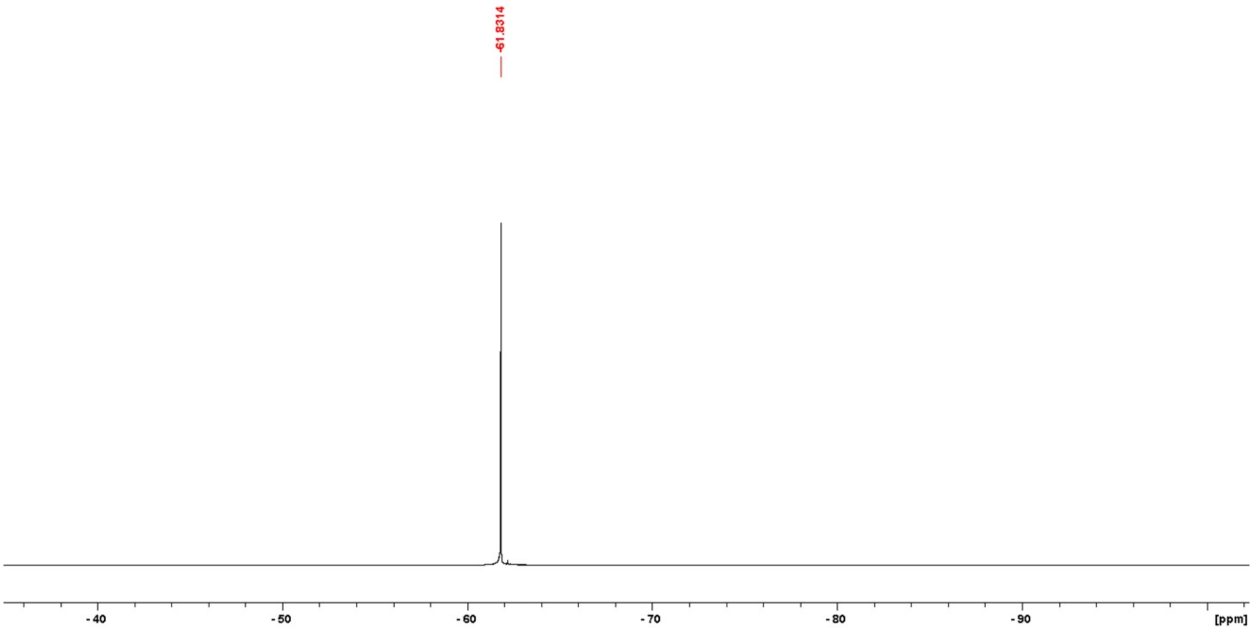

Figure S28. Solid state (ATR) IR spectrum of [HOZr(MesNP^{i}Pr$_2$)$_3$CoCN^{t}Bu][BAr$^F{}_4$] (**[9][BAr$^F{}_4$]**).

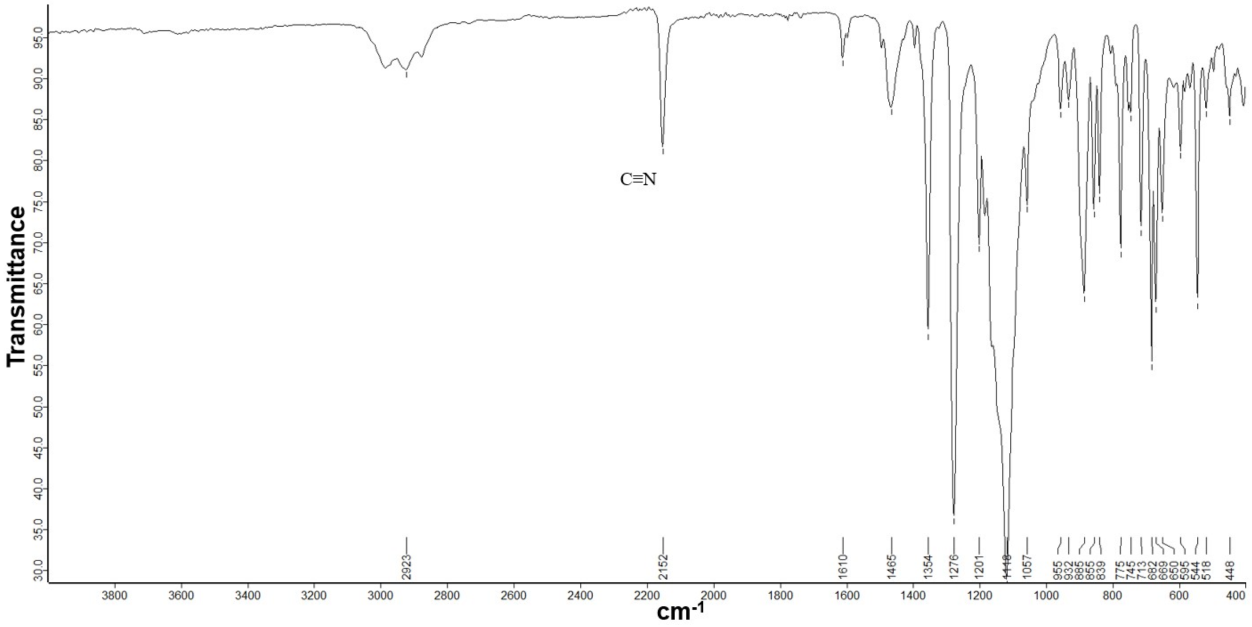

Figure S29. Positive ion ESI-MS spectrum of **[9][BAr$^{F}_{4}$]** in PhF. Two predominant species containing Zr/Co were observed in the full spectrum: [HOZr(MesNP^{i}Pr$_2$)$_3$CoCN^{t}Bu]$^+$ (*m/z* = 999.4539) and [HOZr(MesNP^{i}Pr$_2$)$_3$Co]$^+$ (*m/z* = 916.3691). Isotopic peaks are consistent between predicted (bottom left) and experimental (bottom right) spectra for Zr/Co species.

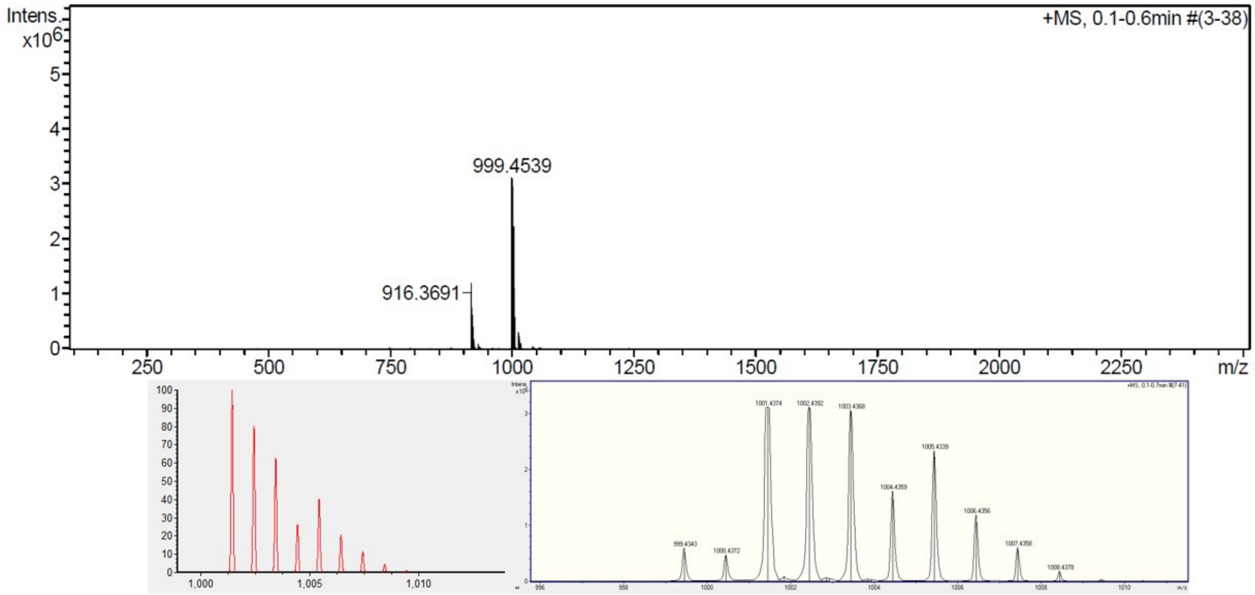

Figure S30. Negative ion ESI-MS spectrum of **[9][BAr$^{F}_{4}$]** in PhF. One predominant species was observed in the full spectrum: [BAr$^{F}_{4}$]$^-$ (*m/z* = 863.0556). Isotopic peaks are consistent between predicted (bottom left) and experimental (bottom right) spectra.

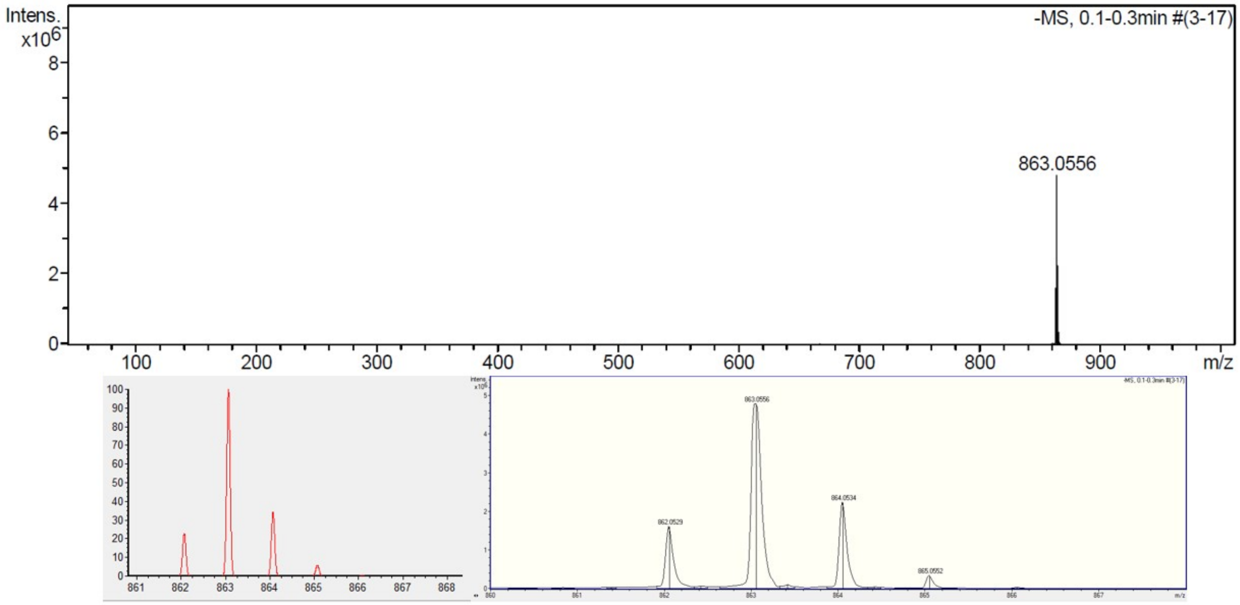

Table S1. X-ray diffraction experimental details for [(μ-Na)OZr(MesNP^{i}Pr$_2$)$_3$CoCN^{t}Bu]$_2$ (**3**), Ph$_3$COZr(MesNP^{i}Pr$_2$)$_3$CoCN^{t}Bu (**4**), and [Ph$_3$COZr(MesNP^{i}Pr$_2$)$_3$CoCN^{t}Bu][BPh$_4$] (**[5][BPh$_4$]**).

	**3**•2C_5H_{12}	**4**•3C_6H_5F	**[5][BPh$_4$]**
chemical formula	$C_{110}H_{192}Co_2N_8Na_2O_2P_6Zr_2$	$C_{87}H_{112.6}CoF_3N_4OP_3Zr$	$C_{93}H_{119}BCoN_4OP_3Zr$
fw (g/mol)	2190.80	1530.48	1562.79
T (K)	120	150	200
λ (Å)	0.71073	0.71073	0.71073
a (Å)	15.7918(6)	20.3878(8)	12.7732(8)
b (Å)	16.3859(6)	20.3878(8)	12.9749(8)
c (Å)	24.0430(9)	16.4288(7)	28.770(2)
α (°)	90	90	87.197(4)
β (°)	103.390(2)	90	83.485(3)
γ (°)	90	120	77.866(3)
V (Å^3)	6052.3(4)	5913.9(5)	4629.9(5)
space group	*P 21/c*	*R 3*	*P -1*
Z	2	3	2
D_{calc} (g/cm^3)	1.202	1.289	1.121
μ (mm^{-1})	0.570	0.459	0.388
R_1 ($I > 2\sigma(I)$), wR_2^{a} (all)	0.0407, 0.1058	0.0206, 0.0522	0.0418, 0.1121

^{a} $R_1 = \Sigma||F_o| - |F_c||/\Sigma|F_o|$, $wR_2 = \{\Sigma[w(F_o^2 - F_c^2)^2]/\Sigma[w(F_o^2)^2]\}^{1/2}$

Table S2. X-ray diffraction experimental details for $Ph_3COZr(MesNP^iPr_2)_3CoCl$ (**6**), $[FZr(MesNP^iPr_2)_3CoCN^tBu][PF_6]$ (**[7][PF$_6$]**), and $HOZr(MesNP^iPr_2)_3CoCN^tBu$ (**8**).

	**6**•2.5C_6H_5F	**[7][PF$_6$]**•2C_6H_5F	**8**
chemical formula	$C_{79}H_{102.5}ClCoF_{2.5}N_3OP_3Zr$	$C_{62}H_{94}CoF_9N_4P_4Zr$	$C_{50}H_{85}CoN_4OP_3Zr$
fw (g/mol)	1436.14	1340.44	1001.27
T (K)	150	150	120
λ (Å)	0.71073	0.71073	0.71073
a (Å)	12.8096(9)	15.3643(8)	36.531(2)
b (Å)	17.9356(12)	19.0177(10)	17.8638(12)
c (Å)	18.3046(11)	22.7503(11)	23.9107(14)
α (°)	86.395(2)	90	90
β (°)	72.314(2)	95.377(2)	108.239(4)
γ (°)	75.548(2)	90	90
V (Å^3)	3879.5(4)	6618.2(6)	14819.8(16)
space group	*P -1*	$P\ 1\ 2_1/n\ 1$	*C2/c*
Z	2	4	8
D_{calc} (g/cm^3)	1.229	1.345	0.898
μ (mm^{-1})	0.494	0.570	0.456
R_1 ($I > 2\sigma(I)$), wR_2[a] (all)	0.0264, 0.0670	0.0259, 0.0710	0.0963, 0.2879

[a] $R_1 = \Sigma||F_o| - |F_c||/\Sigma|F_o|$, $wR_2 = \{\Sigma[w(F_o^2 - F_c^2)^2]/\Sigma[w(F_o^2)^2]\}^{1/2}$

Table S3. X-ray diffraction experimental details for [HOZr(MesNP^{i}Pr$_2$)$_3$CoCN^{t}Bu][BAr$^F{}_4$] (**[9][BAr$^F{}_4$]**).

	**[9][BAr$^F{}_4$]**
chemical formula	$C_{82}H_{97}BCoF_{24}N_4O$ P_3Zr
fw (g/mol)	1865.05
T (K)	150
λ (Å)	0.71073
a (Å)	13.4865(8)
b (Å)	15.0867(9)
c (Å)	24.2764(14)
α (°)	73.132(2)
β (°)	81.938(2)
γ (°)	89.151(2)
V (Å^3)	4678.5(5)
space group	P-1
Z	2
D_{calc} (g/cm^3)	1.324
μ (mm^{-1})	0.432
R_1 ($I > 2\sigma(I)$), wR_2[a] (all)	0.0335, 0.0908

[a] $R_1 = \Sigma||F_o| - |F_c||/\Sigma|F_o|$, $wR_2 = \{\Sigma[w(F_o^2 - F_c^2)^2]/\Sigma[w(F_o^2)^2]\}^{1/2}$

Figure S31. Fully labelled displacement ellipsoid (50%) diagram of **3**•$2C_5H_{12}$. The sodium was disordered across three positions with combined occupancy that converged at 81%, 12% and 7 %. Hydrogens have been omitted for clarity.

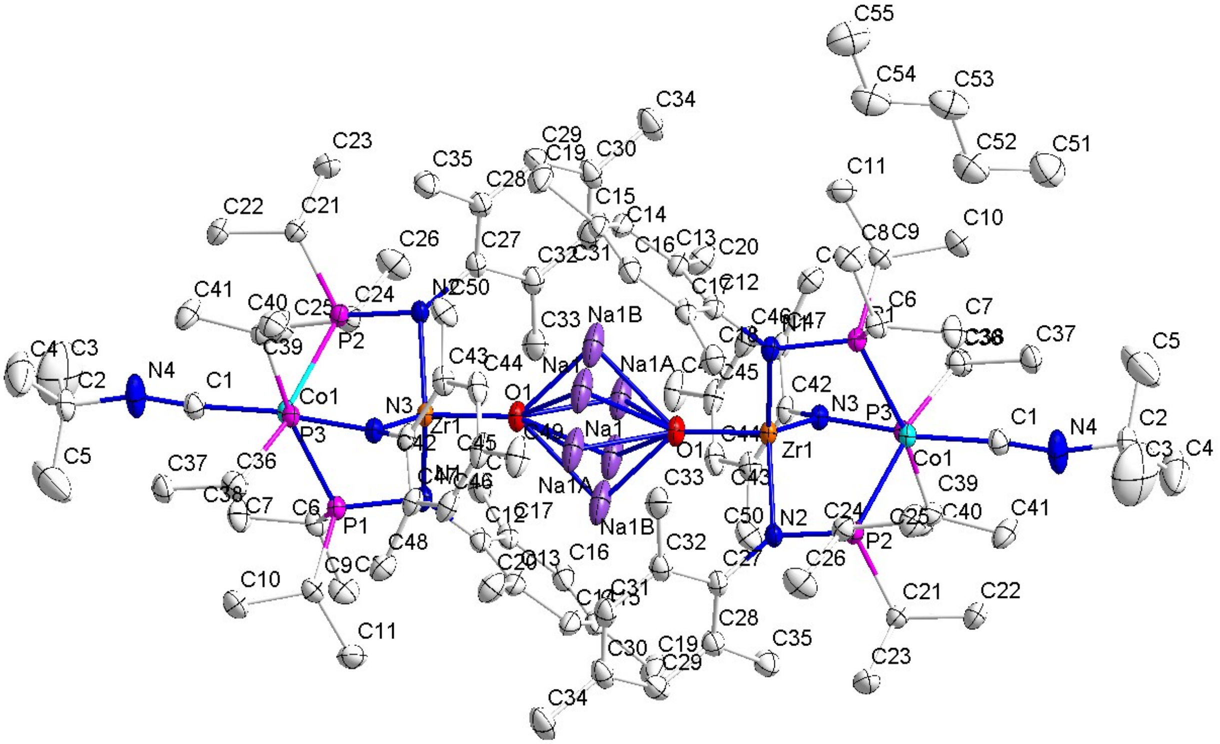

Additional X-ray data collection, solution, and refinement details for 3

The sodium atom was disordered across three positions. The relative occupancy was refined freely, with an EADP constraint on the thermal parameters and a SUMP instruction for their combined occupancy that converged at 81%, 12% and 7%. During the refinement, electron density difference maps revealed disordered solvent that could not be successfully modeled with or without restraints. Thus, the structure factors were modified using the PLATON SQUEEZE[11] technique, in order to produce a "solvate-free" structure factor set. PLATON reported a total electron density of 98 e^- and total solvent accessible volume of 510 $Å^3$, representing a mixture of two THF and pentane molecules.

Figure S32. Fully labelled displacement ellipsoid (50%) diagram of **4**•$3C_6H_5F$. The whole molecule and the fluorobenzene solvent molecule were found to be fully disordered; disordered fluorobenzene solvate molecules, and hydrogens are omitted for clarity.

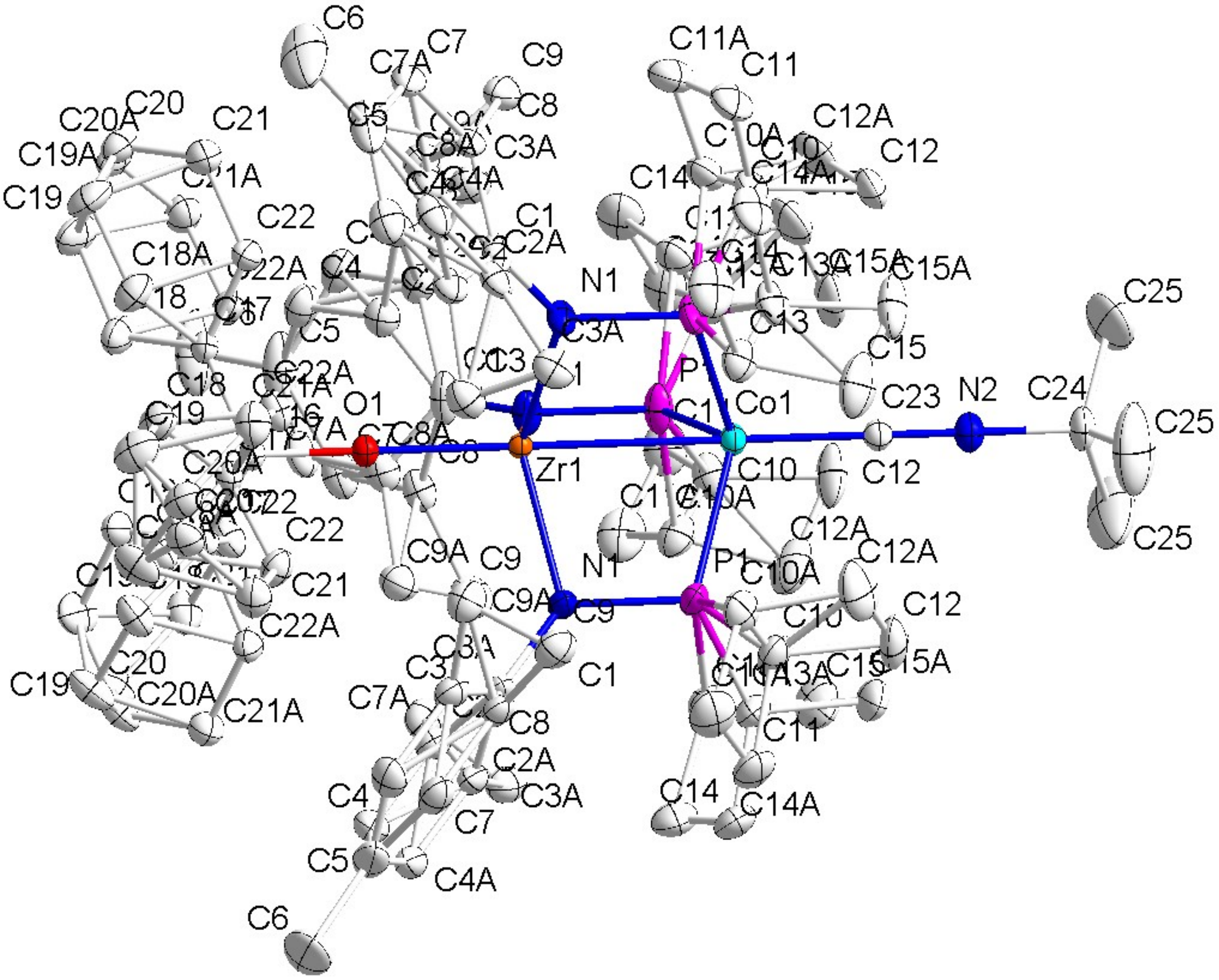

Additional X-ray data collection, solution, and refinement details for 4

During the refinement, it became clear that the reflections corresponding to the Miller indices (0,2,-2), (-2,2,2), (-3,2,1), (-2,3,1), (1,2,-1), and (-1,3,-1) were affected by the shadow of the beamstop (error = >10), and have been omitted. The whole molecule and the fluorobenzene solvent molecule were found to be fully disordered and were modeled with two-component disorder, where the sum of the major and minor components was constrained to be one.

Figure S33. Fully labelled displacement ellipsoid (50%) diagram of **[5][BPh$_4$]**. The ^{t}BuNC group on **[5]**$^+$ and a phenyl group on [BPh$_4$]$^-$ were found to be disordered. Hydrogens have been omitted for clarity.

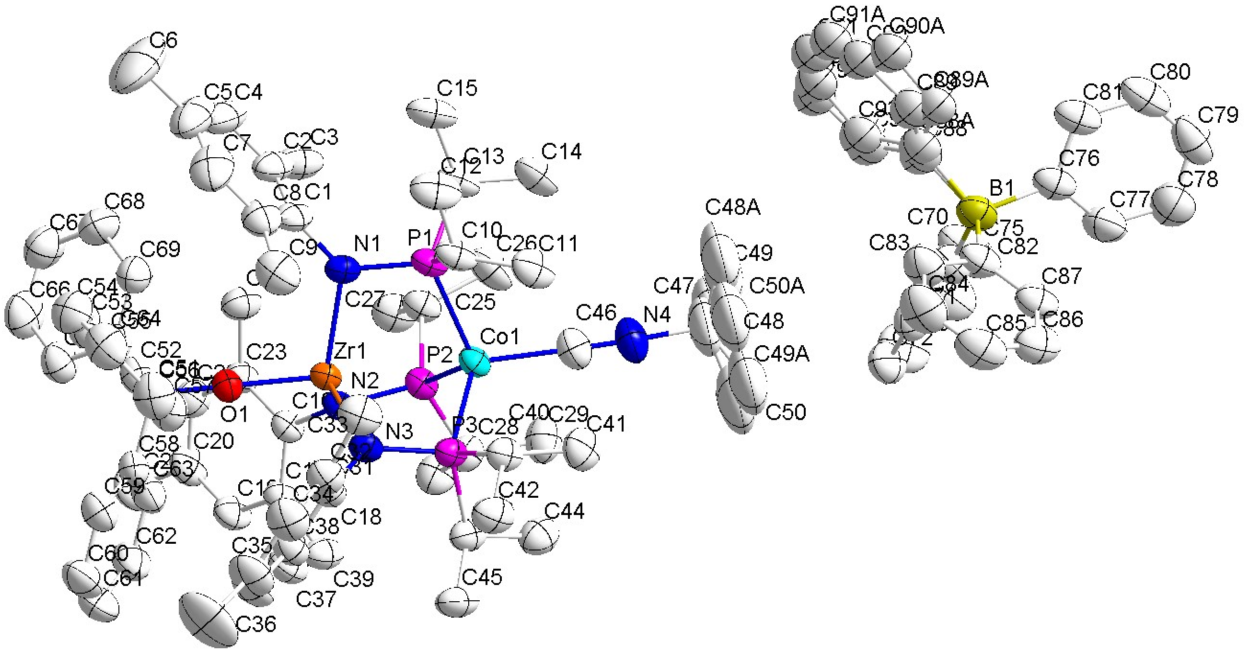

Additional X-ray data collection, solution, and refinement details for [5][BPh$_4$]

One of the phenyl rings of the tetraphenylborate counteranion and the *tert*-butylisocyanide –CH_3 groups were disordered and were modeled with two-component disorder, where the sum of the major and minor components was constrained to be one. EADP constraints were also added to both. During the refinement, it became clear that the reflections corresponding to the Miller indices (0,-2,1), (-1,0,1), (0,2,2), (-2,1,1), (-2,0,3), (2,1,0), (1,-1,1), (0,2,0), (-2,-2,1), (-1,-1,3), (-1,-2,2), (2,0,0), (0,2,3), (0,0,1), (0,-1,1), (-3,0,2) and (-1,2,3) were affected by the shadow of the beamstop (error = >10), and have been omitted. Originally, the Checkcif contained an A-level alert about a "VERY LARGE" solvent accessible void, which contained only one q peak that corresponded to 1.2 e^-, so the structure factors were modified using the PLATON SQUEEZE[11] technique. PLATON reported a total electron density of 31 e^- and total solvent accessible volume of 696 Å^3.

Figure S34. Fully labelled displacement ellipsoid (50%) diagram of **6**•2.5C_6H_5F. Hydrogens have been omitted for clarity.

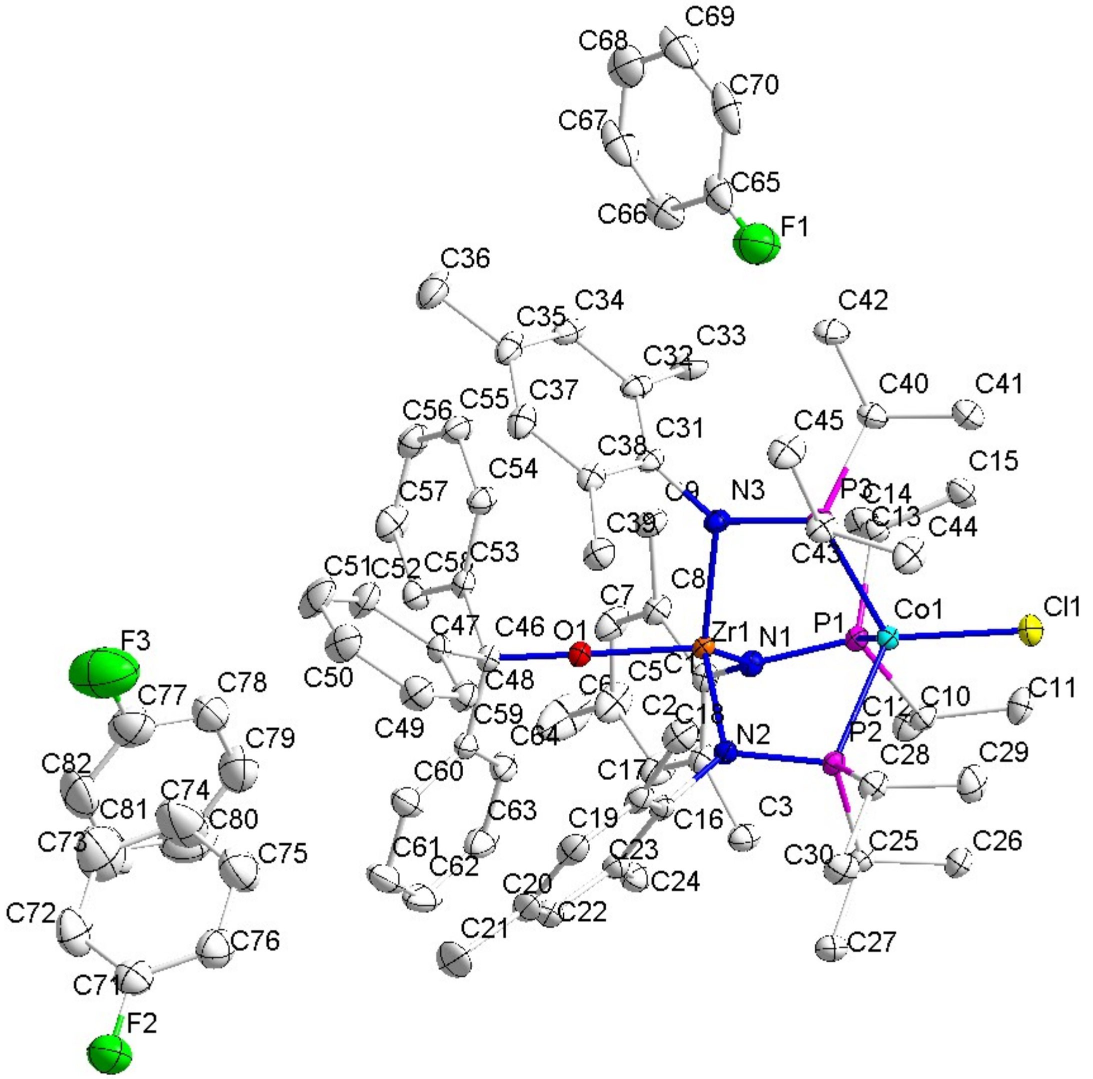

Additional X-ray data collection, solution, and refinement details for 6

There is one B-level alert in the Checkcif that corresponds to the number of reflections missing at low resolution (289/313 92.0 %). One of the fluorobenzene molecules present was located on a special position, so had to be modeled with the PART -1 line. This accounts for the one-half of hydrogen, and fluorine in the formula. Electron density difference maps revealed that there was disordered solvent that could not be successfully modeled, so the structure factors were modified using the PLATON SQUEEZE[11] technique. PLATON reported a total electron density of 92 e^- and total solvent accessible volume of 430 Å^3, likely representing 2 fluorobenzene molecules per unit cell.

Figure S35. Fully labelled displacement ellipsoid (50%) diagram of **[7][PF$_6$]**•2C_6H_5F. [PF$_6$] was found to be disordered. Hydrogens have been omitted for clarity.

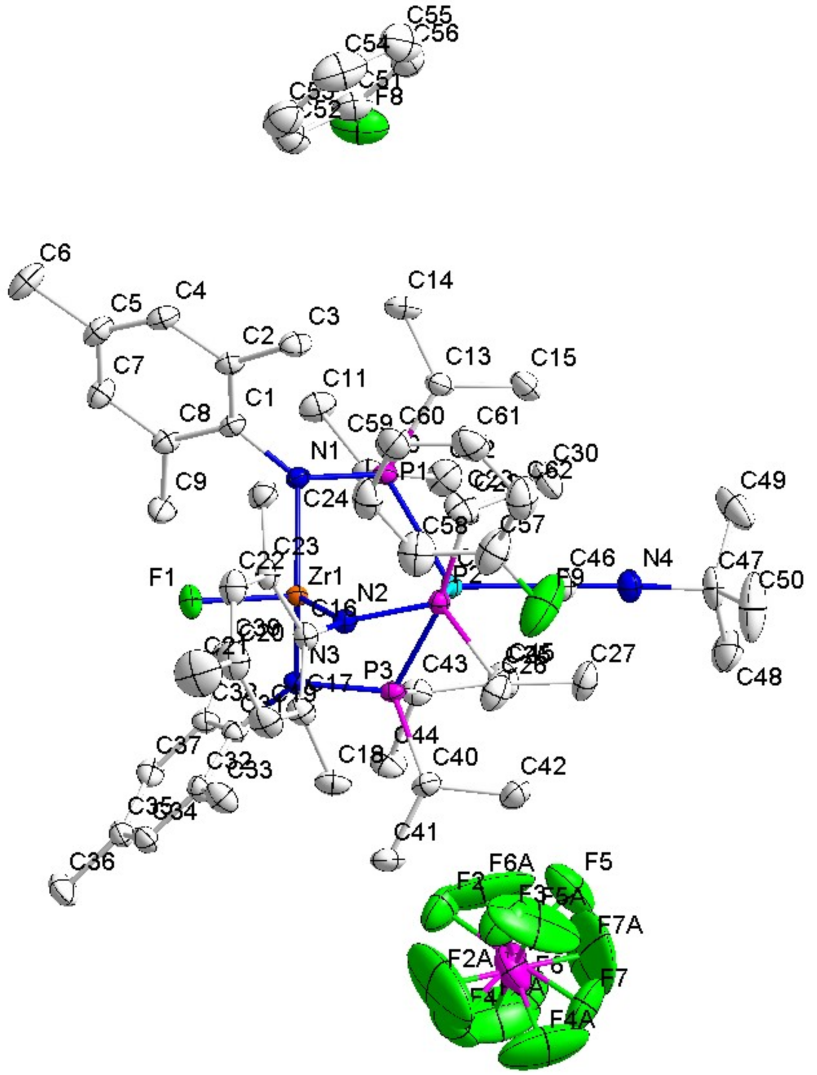

Additional X-ray data collection, solution, and refinement details for [7][PF$_6$]

There is one B-level alert in the Checkcif that corresponds to the number of reflections missing at low resolution (256/279 91.8 %). During the refinement, it became clear that the reflections corresponding to the Miller indices (2,3,2), (-1,0,3), (0,1,3), (-2,1,1), (1,2,1), and (-1,2,2) were affected by the shadow of the beamstop (error = >10), and have been omitted. The hexafluorophosphate counteranion was found to be fully disordered and was modeled with two-component disorder, where the sum of the major and minor components was constrained to be one.

Figure S36. Fully labelled displacement ellipsoid diagram of **8**. Hydrogens except for the hydroxide H have been omitted for clarity.

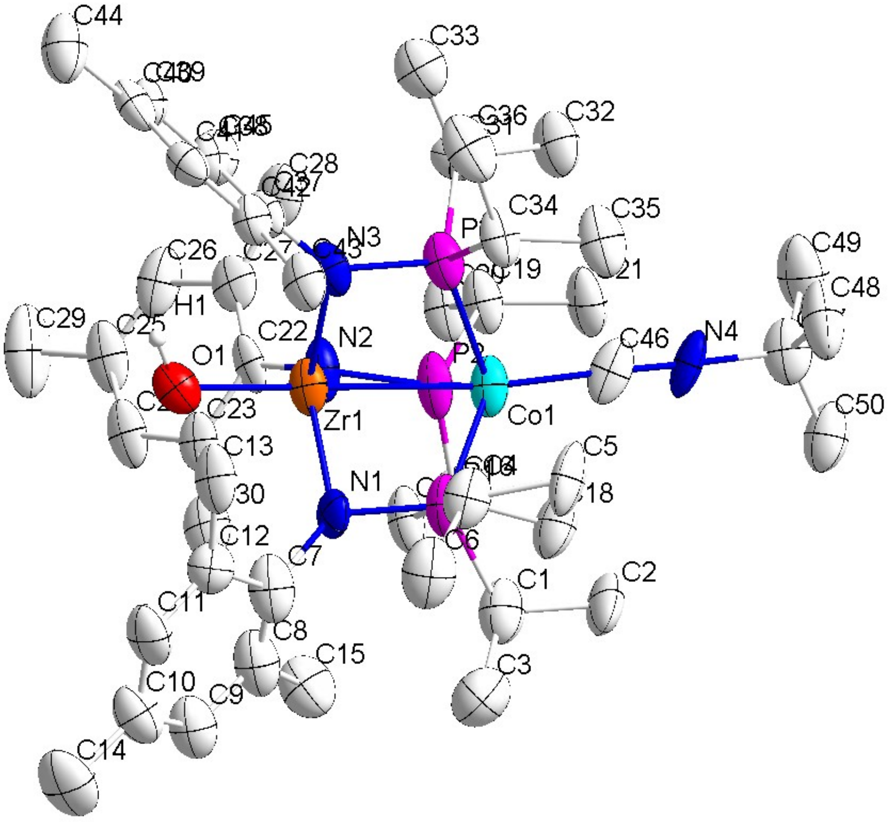

Additional X-ray data collection, solution, and refinement details for 8

Crystals of this compound exhibited very weak diffraction patterns, so a large number of reflections were unobserved. Nonetheless, the structure itself is unequivocally determined. Disordered solvent could not be adequately modeled with or without restraints. Thus, the structure factors were modified using the PLATON SQUEEZE technique[12] in order to produce a "solvate-free" structure factor set. PLATON reported a total electron density of 1708 e^- and total solvent accessible volume of 5637 $Å^3$, likely representing several molecules of pentane.

Figure S37. Fully labelled displacement ellipsoid diagram of **[9][BAr$^F_4$]**, with occupational disorder modeled of hydroxide trace iodide (~1%). Hydrogens except for the hydroxide H have been omitted for clarity.

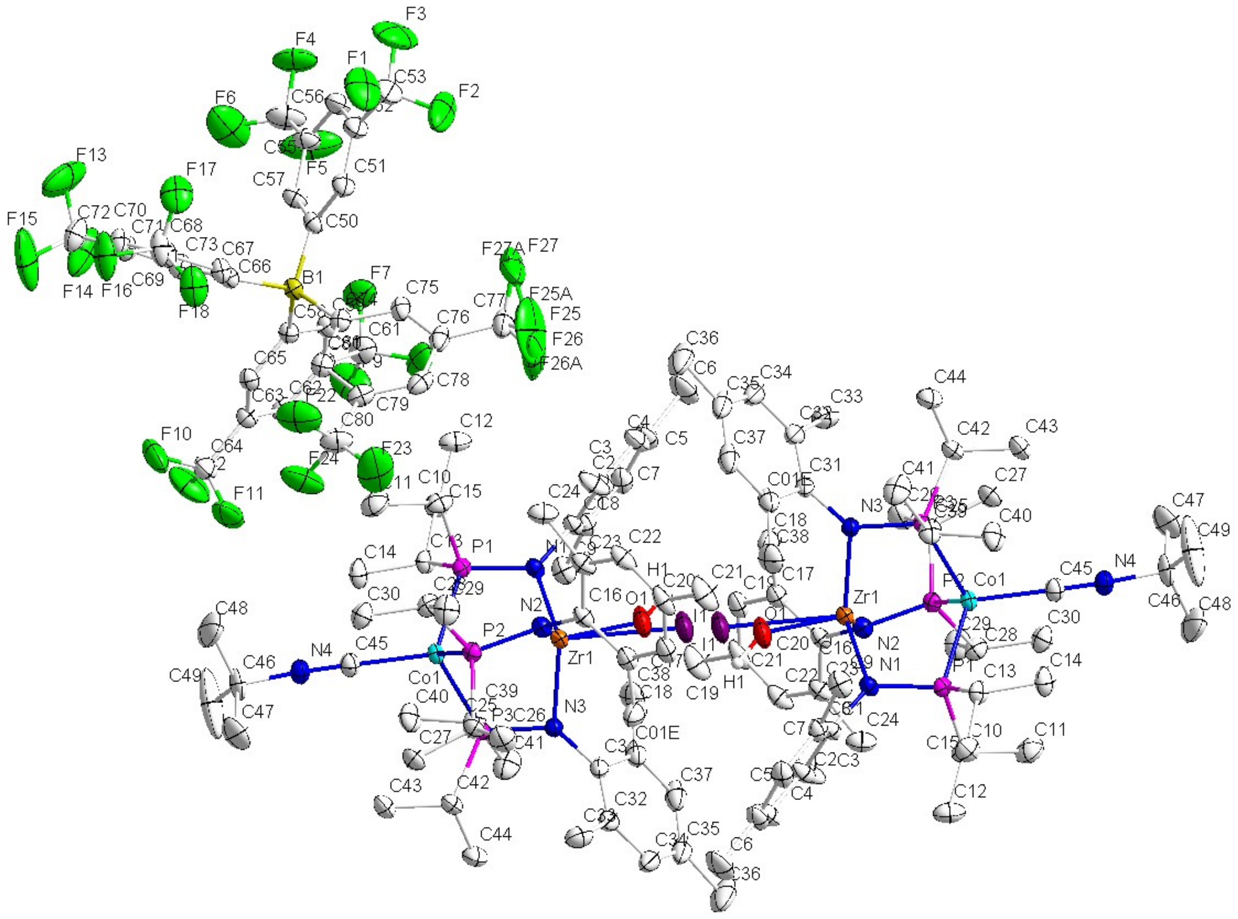

Additional X-ray data collection, solution, and refinement details for [9][BAr$^F_4$]

There is one B-level alert in the Checkcif that corresponds to the number of reflections missing at low resolution (239/366 92.6%). During the refinement, it became clear that the reflections corresponding to the Miller indices (0,2,0), (2,0,1), (0,2,2), (1,-1,2), (1,2,1), (-1,0,3) and (2,0,0) were affected by the shadow of the beamstop (error = >10), and have been omitted. There is occupational disorder of the hydroxide with iodide (~1%), and this was modeled with two-component disorder, where the sum of the major and minor components was constrained to be one. The hydrogen atom of the hydroxide was restrained with DFIX commands. One of the –CF_3 groups of the [BAr$^F_4$]$^-$ counteranion was found to be disordered and was modeled with two-component disorder, where the sum of the major and minor components was constrained to be one. Electron density difference maps revealed that there was disordered solvent that could not be successfully modeled, so the structure factors were modified using the PLATON SQUEEZE[11] technique. PLATON reported a total electron density of 92 e$^-$ and total solvent accessible volume of 430 Å^3, likely representing 2 fluorobenzene molecules per unit cell.

Computational Details

All calculations were performed using Gaussian16 for Linux operating system.[13] Density functional theory calculations were carried out using a combination of Becke's 1988 gradient-corrected exchange functional[14] and Perdew's 1986 electron correlation functional (BP86).[15] A mixed-basis set was employed, using the LANL2TZ(f) triple-ζ basis set with effective core potentials for cobalt and zirconium,[16–18] Gaussian16's internal 6-311+G(d) for heteroatoms (nitrogen, oxygen, and phosphorus), and Gaussian16's internal LANL2DZ basis set (equivalent to D95V)[19] for carbon and hydrogen. Using crystallographically determined geometries as a starting point and modifying as needed, the geometries were optimized to a minimum, followed by analytical frequency calculations to confirm that no imaginary frequencies were present. XYZ coordinates of optimized geometries are provided in Tables S4-S5.

Table S4. Calculated XYZ coordinates for all atoms in [**2**]$^+$ in the S = 3/2 state.
Energy = -3466.032541 Hartree

Symbol	X	Y	Z
Zr	-1.35553	-0.00032	-0.0016
Co	1.71912	0.000413	0.002475
P	0.918561	-1.6875	-1.52276
P	0.91158	-0.47651	2.224648
P	0.916112	2.163787	-0.69771
O	-3.135	-0.00066	-0.00371
N	-0.73206	-1.78335	-1.15316
N	-0.73858	-0.10761	2.119264
N	-0.73398	1.890042	-0.96739
N	4.821327	0.002646	0.008061
C	3.635085	0.001845	0.006297
C	6.287853	0.002472	0.008911
C	6.764141	-0.17396	-1.46091
H	6.404274	-1.12851	-1.88282
H	7.867796	-0.17933	-1.47927
H	6.41004	0.656657	-2.09614
C	6.762796	-1.18248	0.897038
H	6.402613	-1.07012	1.934492
H	7.866431	-1.19657	0.911335
H	6.408088	-2.1477	0.495347
C	6.763269	1.363725	0.591511
H	6.403568	2.206422	-0.02423
H	7.866916	1.382687	0.596736
H	6.408522	1.498276	1.628278

C	1.174076	-1.09532	-3.30903
H	0.667524	-0.10885	-3.26281
C	0.483716	-1.92712	-4.42714
H	1.07611	-2.82059	-4.68654
H	0.408094	-1.30529	-5.34055
H	-0.53134	-2.25609	-4.15677
C	2.665918	-0.84921	-3.66149
H	3.216584	-0.33514	-2.85317
H	2.732056	-0.22198	-4.57137
H	3.184273	-1.7989	-3.88491
C	1.786242	-3.36718	-1.33271
H	1.401292	-3.71067	-0.35359
C	3.331097	-3.26876	-1.21665
H	3.796458	-2.95163	-2.16577
H	3.73448	-4.26988	-0.96881
H	3.648136	-2.57055	-0.42463
C	1.405139	-4.42687	-2.40587
H	0.325525	-4.45548	-2.62468
H	1.707827	-5.4293	-2.04494
H	1.948083	-4.2446	-3.35005
C	-1.7167	-2.72382	-1.69638
C	-2.63378	-2.27193	-2.70854
C	-3.58555	-3.17257	-3.2397
H	-4.26855	-2.80999	-4.01979
C	-3.70481	-4.50907	-2.78289
C	-2.84635	-4.9122	-1.73707
H	-2.94137	-5.92699	-1.32691
C	-1.86741	-4.04867	-1.17524
C	-1.05956	-4.59456	-0.00741
H	-0.29912	-5.32902	-0.33694
H	-1.72454	-5.11808	0.703341
H	-0.54827	-3.78594	0.53754
C	-4.73739	-5.45362	-3.37783
H	-5.75919	-5.03826	-3.28923
H	-4.72755	-6.43546	-2.87332
H	-4.55033	-5.6263	-4.4552
C	-2.67072	-0.82972	-3.18161
H	-3.2822	-0.2209	-2.48736
H	-3.12413	-0.75271	-4.18593
H	-1.66793	-0.37474	-3.21952
C	1.164628	-2.3201	2.602656
H	0.65805	-2.77071	1.723893
C	0.47278	-2.87359	3.880711
H	1.066732	-2.65666	4.78441
H	0.392457	-3.97496	3.795752

H	-0.5405	-2.47107	4.032237
C	2.655922	-2.74997	2.565251
H	3.206261	-2.30654	1.71593
H	2.720951	-3.85153	2.475788
H	3.175017	-2.47009	3.499479
C	1.775988	0.526032	3.587912
H	1.392252	1.546178	3.39568
C	3.321238	0.576767	3.449888
H	3.785415	-0.40452	3.64873
H	3.722509	1.289728	4.196258
H	3.641695	0.916249	2.451231
C	1.38977	0.124901	5.04044
H	0.309476	-0.04879	5.171037
H	1.691568	0.937232	5.730222
H	1.930396	-0.7852	5.354974
C	-1.72587	-0.10669	3.202866
C	-1.87814	1.007935	4.088249
C	-2.86019	0.95498	5.114134
H	-2.95641	1.818281	5.786828
C	-3.72006	-0.15141	5.286408
C	-3.59866	-1.21646	4.358954
H	-4.28239	-2.07284	4.434023
C	-2.64379	-1.20867	3.316223
C	-2.67824	-2.34091	2.305211
H	-3.28062	-2.04242	1.425061
H	-3.14062	-3.24573	2.738404
H	-1.67387	-2.60799	1.939269
C	-4.75646	-0.19222	6.398417
H	-4.57637	-1.04208	7.084563
H	-5.77762	-0.31627	5.990564
H	-4.74359	0.733718	6.999328
C	-1.06832	2.291139	3.978617
H	-0.31113	2.373032	4.782539
H	-1.73251	3.169458	4.072569
H	-0.55279	2.356591	3.007959
C	1.784544	2.840854	-2.24648
H	1.400149	2.16521	-3.03425
C	1.402576	4.300123	-2.62624
H	0.322678	4.50256	-2.54236
H	1.705963	4.490184	-3.67438
H	1.944217	5.026544	-1.99505
C	3.329364	2.691722	-2.21915
H	3.794346	3.352034	-1.46701
H	3.733017	2.981956	-3.20872
H	3.646603	1.655903	-2.015

C	1.167825	3.414585	0.709017
H	0.659319	2.881064	1.538892
C	0.477844	4.79882	0.54604
H	1.072555	5.470724	-0.0954
H	0.398401	5.278256	1.541288
H	-0.53565	4.729228	0.122096
C	2.6587	3.59656	1.101822
H	3.20829	2.639022	1.14567
H	2.722727	4.072248	2.099469
H	3.179347	4.263521	0.391434
C	-1.71838	2.82949	-1.51268
C	-2.63742	3.480027	-0.61719
C	-3.58836	4.390011	-1.13365
H	-4.2726	4.884618	-0.43102
C	-3.70513	4.662083	-2.51984
C	-2.84467	3.95811	-3.39
H	-2.93735	4.110298	-4.47412
C	-1.86659	3.03996	-2.92093
C	-1.05667	2.301243	-3.9758
H	-0.29609	2.954043	-4.44664
H	-1.72025	1.946183	-4.78513
H	-0.54539	1.425786	-3.54635
C	-4.73927	5.646666	-3.04286
H	-5.7645	5.336513	-2.76437
H	-4.70008	5.730328	-4.1429
H	-4.5812	6.65782	-2.62129
C	-2.67699	3.168647	0.868216
H	-3.28476	2.260153	1.046677
H	-3.136	3.997762	1.435689
H	-1.67454	2.97863	1.284055

Table S5. Calculated XYZ coordinates for all atoms in [**2**]$^+$ in the S = 1/2 state.

Energy = -3466.023205 Hartree

Symbol	X	Y	Z
Zr	-1.28495	-0.03758	0.421803
Co	1.641446	0.012905	-0.70115
P	1.231888	2.003647	0.511963
P	-0.0711	-0.06	-2.36144
P	1.347835	-2.00099	0.598904
O	-2.90928	-0.0485	1.155703
N	-0.43529	1.991571	0.770792
N	-1.56993	-0.46502	-1.70157
N	-0.10768	-1.64724	1.388717
N	4.625305	0.154087	-0.90103
C	3.468518	0.09908	-0.61781
C	6.038156	0.190058	-1.28913
C	6.864516	0.663704	-0.05937
H	6.557708	1.674713	0.260097
H	7.932443	0.695612	-0.33741
H	6.748046	-0.03093	0.790836
C	6.188963	1.18691	-2.47356
H	5.58422	0.866816	-3.34036
H	7.248609	1.218001	-2.78193
H	5.883397	2.205488	-2.17829
C	6.45643	-1.2447	-1.72046
H	6.330921	-1.96214	-0.8913
H	7.520819	-1.23445	-2.01356
H	5.861062	-1.58737	-2.58509
C	2.110594	1.934984	2.206466
H	1.707419	0.977279	2.592971
C	1.695678	3.061497	3.201242
H	2.302653	3.969455	3.046433
H	1.889918	2.707387	4.232299
H	0.634307	3.341077	3.136921
C	3.65524	1.821583	2.187695
H	4.015735	0.98015	1.577045
H	4.009501	1.664101	3.225391
H	4.127373	2.752449	1.824865
C	1.811897	3.572071	-0.38851
H	1.086998	3.640816	-1.22232
C	3.230824	3.453443	-1.00422
H	4.011065	3.391863	-0.22567
H	3.434951	4.358422	-1.60886

H	3.334363	2.576033	-1.66387
C	1.714601	4.878263	0.451859
H	0.777211	4.959673	1.024881
H	1.775415	5.748224	-0.23083
H	2.561526	4.95753	1.15585
C	-1.27371	3.012034	1.415105
C	-1.72733	2.805556	2.765538
C	-2.53904	3.782442	3.387207
H	-2.86478	3.607021	4.421418
C	-2.9635	4.957342	2.71874
C	-2.56637	5.110546	1.372797
H	-2.91148	5.987876	0.808696
C	-1.74644	4.162225	0.703708
C	-1.45581	4.427612	-0.76513
H	-0.70498	5.229567	-0.90319
H	-2.37532	4.75545	-1.28294
H	-1.08731	3.520164	-1.26626
C	-3.82861	5.992734	3.419621
H	-4.7256	5.52828	3.870598
H	-4.16844	6.77737	2.721375
H	-3.27429	6.489873	4.239292
C	-1.42289	1.538871	3.54462
H	-2.19221	0.772602	3.328831
H	-1.42871	1.731189	4.632583
H	-0.44469	1.107968	3.276324
C	-0.15952	1.602744	-3.27314
H	-0.41016	2.274507	-2.42799
C	-1.28412	1.750439	-4.33721
H	-1.00706	1.267177	-5.28876
H	-1.43409	2.827909	-4.54608
H	-2.2479	1.332673	-4.00954
C	1.199677	2.065192	-3.86276
H	2.049479	1.883601	-3.17989
H	1.160334	3.151641	-4.07238
H	1.412503	1.559291	-4.82135
C	0.577774	-1.3349	-3.61569
H	0.379847	-2.29441	-3.10195
C	2.112682	-1.21986	-3.83685
H	2.370697	-0.34784	-4.46074
H	2.478491	-2.12473	-4.35946
H	2.668866	-1.13121	-2.88371
C	-0.16285	-1.35356	-4.98088
H	-1.25928	-1.30192	-4.87764
H	0.087044	-2.29138	-5.51509
H	0.165206	-0.51672	-5.62243

C	-2.83629	-0.7062	-2.38604
C	-3.14221	-1.97774	-2.96644
C	-4.39504	-2.1651	-3.61044
H	-4.6107	-3.14638	-4.05494
C	-5.37244	-1.14823	-3.67861
C	-5.07811	0.07991	-3.0336
H	-5.83594	0.874875	-3.02427
C	-3.84844	0.314911	-2.37648
C	-3.66774	1.620403	-1.62216
H	-3.83076	1.459102	-0.53873
H	-4.39429	2.378328	-1.96465
H	-2.65394	2.038775	-1.74125
C	-6.70076	-1.36422	-4.38697
H	-6.80502	-0.68947	-5.25864
H	-7.55383	-1.15719	-3.71355
H	-6.80208	-2.4012	-4.7518
C	-2.21539	-3.18197	-2.88269
H	-1.70104	-3.38451	-3.842
H	-2.79425	-4.08941	-2.63171
H	-1.45086	-3.03975	-2.1028
C	2.685528	-2.44035	1.882415
H	2.612109	-1.58672	2.582164
C	2.403338	-3.75158	2.676812
H	1.34807	-3.88148	2.960382
H	3.004916	-3.73776	3.606589
H	2.723318	-4.63549	2.097748
C	4.145463	-2.48305	1.35971
H	4.292364	-3.26282	0.593092
H	4.810853	-2.73093	2.209855
H	4.473106	-1.5183	0.947696
C	1.119747	-3.51949	-0.5212
H	0.330208	-3.14458	-1.20374
C	0.578851	-4.81346	0.151128
H	1.390866	-5.38637	0.629113
H	0.134205	-5.46123	-0.62949
H	-0.19519	-4.61864	0.908626
C	2.384017	-3.84184	-1.36319
H	2.891058	-2.93959	-1.74736
H	2.102719	-4.46819	-2.23169
H	3.114082	-4.42117	-0.77059
C	-0.74719	-2.35238	2.505532
C	-1.88869	-3.18484	2.221546
C	-2.51694	-3.88585	3.276148
H	-3.37518	-4.52768	3.035368
C	-2.1024	-3.76384	4.625797

C	-1.03231	-2.88258	4.894081
H	-0.71117	-2.73074	5.933705
C	-0.3555	-2.16757	3.87027
C	0.745716	-1.21849	4.315161
H	1.650842	-1.76529	4.644458
H	0.404725	-0.61412	5.175329
H	1.032956	-0.52922	3.506363
C	-2.81061	-4.52223	5.736816
H	-3.86667	-4.20289	5.827882
H	-2.32496	-4.35821	6.714447
H	-2.81797	-5.61107	5.539916
C	-2.50978	-3.28053	0.838316
H	-3.25743	-2.47288	0.713025
H	-3.02961	-4.24577	0.701837
H	-1.76958	-3.16901	0.030377

Table S6. Calculated XYZ coordinates for all atoms in **2**.

Symbol	X	Y	Z
Zr	1.299216	0.049944	0.098253
Co	-1.67371	-0.11255	-0.26237
P	-1.16531	0.235299	2.036054
P	-0.74158	-2.23693	-0.87177
P	-0.88442	1.817606	-1.42235
O	3.081215	0.116696	0.263073
N	0.531562	-0.02021	2.154107
N	0.918974	-1.83951	-1.01139
N	0.756391	1.936358	-0.94813
N	-4.71766	-0.06317	-0.3397
C	-3.5179	-0.06593	-0.28119
C	-6.16957	-0.09861	-0.37994
C	-6.71563	0.760819	0.796789
H	-6.37305	0.357052	1.765639
H	-7.82045	0.750341	0.780487
H	-6.37166	1.806423	0.708808
C	-6.63094	-1.57812	-0.23665
H	-6.2225	-2.19371	-1.05768
H	-7.73446	-1.62797	-0.27287
H	-6.29087	-2.00089	0.725196
C	-6.63413	0.48666	-1.74496
H	-6.28773	1.5286	-1.86121
H	-7.73778	0.4728	-1.79603
H	-6.23492	-0.11273	-2.58231
C	-1.54941	2.011849	2.616998
H	-0.99318	2.581195	1.842888
C	-1.02408	2.467245	4.007168
H	-1.70413	2.149547	4.816952
H	-0.97606	3.574731	4.035686
H	-0.01987	2.07852	4.234793
C	-3.05088	2.375206	2.468893
H	-3.47043	2.018762	1.512408
H	-3.17753	3.475819	2.514867
H	-3.64621	1.944197	3.295198
C	-2.06991	-0.92983	3.249948
H	-1.59977	-1.90179	3.005731
C	-3.58851	-1.07601	2.964518
H	-4.14044	-0.15151	3.210987
H	-4.00054	-1.88819	3.597668
H	-3.78902	-1.32401	1.910366
C	-1.8573	-0.64821	4.763627

H	-0.81187	-0.40959	5.017108
H	-2.16023	-1.53956	5.34987
H	-2.49462	0.190913	5.097679
C	1.382624	-0.04467	3.332474
C	2.222529	1.087776	3.630579
C	3.059162	1.067373	4.770443
H	3.681154	1.949649	4.976999
C	3.141015	-0.05457	5.632201
C	2.378119	-1.18938	5.283108
H	2.45752	-2.0988	5.896335
C	1.519154	-1.21433	4.150862
C	0.824032	-2.53132	3.843087
H	-0.01347	-2.73297	4.538829
H	1.537604	-3.37073	3.939325
H	0.427422	-2.53138	2.816494
C	4.031081	-0.03829	6.866922
H	5.039866	0.351245	6.63249
H	4.148713	-1.0513	7.29251
H	3.607989	0.60997	7.660374
C	2.292931	2.288472	2.706993
H	2.909283	2.044952	1.819256
H	2.753735	3.15495	3.215446
H	1.298554	2.586366	2.339783
C	-0.97149	-3.69057	0.336464
H	-0.53847	-3.24599	1.256043
C	-0.20992	-5.01613	0.058721
H	-0.72651	-5.62502	-0.70334
H	-0.17254	-5.61779	0.989615
H	0.825019	-4.85042	-0.27961
C	-2.47194	-3.97412	0.620109
H	-3.05947	-3.04323	0.713809
H	-2.57538	-4.54457	1.564225
H	-2.92035	-4.58824	-0.18248
C	-1.44009	-2.81077	-2.55108
H	-1.05903	-2.01542	-3.22098
C	-2.99154	-2.74765	-2.60782
H	-3.4544	-3.55141	-2.00718
H	-3.32478	-2.88237	-3.65696
H	-3.37884	-1.78177	-2.2418
C	-0.94873	-4.18061	-3.095
H	0.135212	-4.3297	-2.96298
H	-1.17692	-4.2509	-4.17813
H	-1.47647	-5.01498	-2.59856
C	1.984702	-2.67493	-1.54336

C	2.254608	-2.75115	-2.94853
C	3.312645	-3.57486	-3.42051
H	3.492921	-3.62235	-4.50409
C	4.142364	-4.31348	-2.54992
C	3.912114	-4.17006	-1.15837
H	4.571148	-4.69013	-0.44901
C	2.875373	-3.35888	-0.64154
C	2.777997	-3.17652	0.861613
H	3.188925	-2.18815	1.147329
H	3.354939	-3.95439	1.394326
H	1.73593	-3.20828	1.217335
C	5.255467	-5.20926	-3.07516
H	5.051059	-6.27626	-2.85654
H	6.227466	-4.96209	-2.60649
H	5.371935	-5.10798	-4.16912
C	1.485007	-1.94416	-3.98328
H	0.802626	-2.57852	-4.58213
H	2.185058	-1.46145	-4.69037
H	0.887113	-1.15852	-3.49603
C	-1.79923	3.479803	-1.21181
H	-1.55871	3.729064	-0.15987
C	-1.34005	4.661971	-2.11081
H	-0.24504	4.727025	-2.21572
H	-1.70211	5.616281	-1.67695
H	-1.77918	4.579173	-3.12125
C	-3.34171	3.325761	-1.30485
H	-3.67088	3.160653	-2.3471
H	-3.82335	4.259412	-0.95008
H	-3.71182	2.489112	-0.69039
C	-0.94305	1.399359	-3.28417
H	-0.3231	0.478074	-3.27804
C	-0.29147	2.393652	-4.28411
H	-0.97247	3.231512	-4.51478
H	-0.08104	1.869381	-5.23826
H	0.656647	2.814471	-3.91166
C	-2.36358	0.994795	-3.76363
H	-2.87557	0.340917	-3.0342
H	-2.295	0.451536	-4.7275
H	-2.99982	1.883107	-3.93413
C	1.73582	2.933712	-1.34729
C	2.738965	2.601152	-2.32568
C	3.706693	3.562266	-2.69975
H	4.454769	3.283962	-3.45531
C	3.760008	4.85241	-2.11509

C	2.811929	5.144416	-1.11119
H	2.847439	6.120487	-0.60597
C	1.815958	4.214177	-0.70656
C	0.902015	4.631645	0.435727
H	0.153223	5.381403	0.113661
H	1.492625	5.090738	1.250126
H	0.365174	3.762934	0.845893
C	4.828592	5.857488	-2.522
H	5.829578	5.54471	-2.16342
H	4.62184	6.85856	-2.10238
H	4.894316	5.956746	-3.62263
C	2.833586	1.210295	-2.92424
H	3.268677	0.509544	-2.18447
H	3.485015	1.205628	-3.81695
H	1.847307	0.812787	-3.21227

Figure S38. ^{19}F and $^{31}P\{^1H\}$ NMR spectra of the reaction mixture between **2** and $FcPF_6$, revealing multiple PF_6^- derived byproducts that contain both ^{31}P and ^{19}F nuclei. The doublet centered at -56 ppm in the ^{19}F NMR spectrum (J_{PF} = 900 Hz) and the quintet centered at -68 ppm in the ^{31}P NMR spectrum (J_{PF} =900 Hz) are tentatively assigned to a species of the general form PF_4OR (where R could potentially = H) based on several literature reports *(Makromol. Chem.* **1979**, *180,* 1509-1519*; J. Am. Chem. Soc.* **1997**, *119,* 3918-3928*)*.

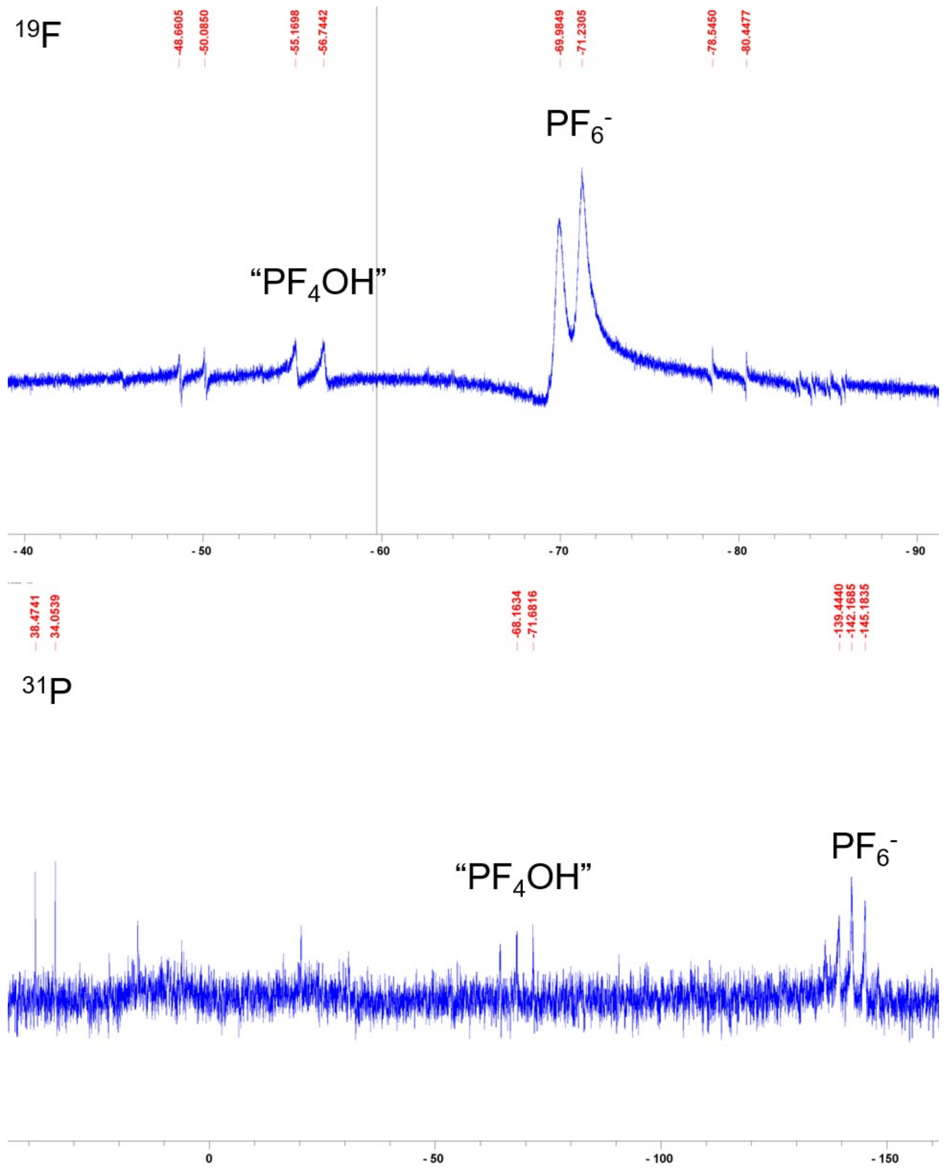

References

1 B. P. Greenwood, G. T. Rowe, C.-H. Chen, B. M. Foxman and C. M. Thomas, *J. Am. Chem. Soc.*, 2010, **132**, 44–45.

2 J. P. Krogman, M. W. Bezpalko, B. M. Foxman and C. M. Thomas, *Dalton Trans.*, 2016, **45**, 11182–11190.

3 N. A. Yakelis and R. G. Bergman, *Organometallics*, 2005, **24**, 3579–3581.

4 F. Calderazzo, G. Pampaloni, L. Rocchi and U. Englert, *Organometallics*, 1994, **13**, 2592–2601.

5 M. Gomberg, *J. Am. Chem. Soc.*, 1900, **22**, 752–757.

6 E. S. Jang, C. L. McMullin, M. Käß, K. Meyer, T. R. Cundari and T. H. Warren, *J. Am. Chem. Soc.*, 2014, **136**, 10930–10940.

7 D. F. Evans, *J. Chem. Soc.*, 1959, 2003.

8 S. K. Sur, *J. Magn. Reson.*, 1989, **82**, 169–173.

9 S. Stoll and A. Schweiger, *J. Magn. Reson.*, 2006, **178**, 42–55.

10 R. Karlsson, *J. Chem. Eng. Data*, 1973, **18**, 290–292.

11 A. L. Spek, *Acta Crystallogr. Sect. C Struct. Chem.*, 2015, **71**, 9–18.

12 A. L. Spek, *Acta Crystallogr. Sect. C Struct. Chem.*, 2015, **71**, 9–18.

13 M. J. Frisch, G. W. Trucks, H. B. Schlegel, G. E. Scuseria, M. A. Robb, J. R. Cheeseman, V. Scalmani, G.; Barone, G. A. Petersson, H. Nakatsuji, X. Li, M. Caricato, A. V. Marenich, J. Bloino, B. G. Janesko, R. Gomperts, B. Mennucci, H. P. Hratchian, J. V. Ortiz, A. F. Izmaylov, J. L. Sonnenberg, D. Williams-Young, F. Ding, F. Lipparini, F. Egidi, J. Goings, B. Peng, A. Petrone, T. Henderson, D. Ranasinghe, V. G. Zakrzewski, J. Gao, N. Rega, G. Zheng, W. Liang, M. Hada, M. Ehara, K. Toyota, R. Fukuda, J. Hasegawa, M. Ishida, T. Nakajima, Y. Honda, O. Kitao, H. Nakai, T. Vreven, K. Throssell, J. Montgomery, J. A., J. E. Peralta, F. Ogliaro, M. J. Bearpark, J. J. Heyd, E. N. Brothers, K. N. Kudin, V. N. Staroverov, T. A. Keith, R. Kobayashi, J. Normand, K. Raghavachari, A. P. Rendell, J. C. Burant, S. S. Iyengar, J. Tomasi, M. Cossi, J. M. Millam, M. Klene, C. Adamo, R. Cammi, J. W. Ochterski, R. L. Martin, K. Morokuma, O. Farkas, J. B. Foresman and D. J. Fox, Gaussian Inc., 2016.

14 A. D. Becke, *Phys. Rev. A*, 1988, **38**, 3098–3100.

15 J. P. Perdew, *Phys. Rev. B*, 1986, **33**, 8822–8824.

16 P. J. Hay and W. R. Wadt, *J. Chem. Phys.*, 1985, **82**, 270–283.

17 P. J. Hay and W. R. Wadt, *J. Chem. Phys.*, 1985, **82**, 299–310.

18 W. R. Wadt and P. J. Hay, *J. Chem. Phys.*, 1985, **82**, 284–298.

19 T. H. Dunning and P. J. Hay, *Modern Theoretical Chemistry*, Plenum, New York, 3rd. ed., 1976.
